# A combined physicochemical approach towards human tenocyte phenotype maintenance

**DOI:** 10.1016/j.mtbio.2021.100130

**Published:** 2021-09-10

**Authors:** C.N.M. Ryan, E. Pugliese, N. Shologu, D. Gaspar, P. Rooney, Md N. Islam, A. O'Riordan, M.J. Biggs, M.D. Griffin, D.I. Zeugolis

**Affiliations:** a Regenerative, Modular & Developmental Engineering Laboratory (REMODEL), Biomedical Sciences Building, National University of Ireland Galway (NUI Galway), Galway, Ireland; b Science Foundation Ireland (SFI) Centre for Research in Medical Devices (CÚRAM), Biomedical Sciences Building, National University of Ireland Galway (NUI Galway), Galway, Ireland; c Regenerative Medicine Institute (REMEDI), School of Medicine, Biomedical Sciences Building, National University of Ireland Galway (NUI Galway), Galway, Ireland; d Discipline of Biochemistry, School of Natural Sciences, National University of Ireland Galway (NUI Galway), Galway, Ireland; e Tyndall National Institute, University College Cork (UCC), Cork, Ireland; f Regenerative, Modular & Developmental Engineering Laboratory (REMODEL), Charles Institute of Dermatology, Conway Institute of Biomolecular & Biomedical Research and School of Mechanical & Materials Engineering, University College Dublin (UCD), Dublin, Ireland

**Keywords:** Collagen type I coating, Surface topography, Substrate elasticity, Macromolecular crowding, Cell phenotype maintenance, *In vitro* microenvironment

## Abstract

During *in vitro* culture, bereft of their optimal tissue context, tenocytes lose their phenotype and function. Considering that tenocytes in their native tissue milieu are exposed simultaneously to manifold signals, combination approaches (e.g. growth factor supplementation and mechanical stimulation) are continuously gaining pace to control cell fate during *in vitro* expansion, albeit with limited success due to the literally infinite number of possible permutations. In this work, we assessed the potential of scalable and potent physicochemical approaches that control cell fate (substrate stiffness, anisotropic surface topography, collagen type I coating) and enhance extracellular matrix deposition (macromolecular crowding) in maintaining human tenocyte phenotype in culture. Cell morphology was primarily responsive to surface topography. The tissue culture plastic induced the largest nuclei area, the lowest aspect ratio, and the highest focal adhesion kinase. Collagen type I coating increased cell number and metabolic activity. Cell viability was not affected by any of the variables assessed. Macromolecular crowding intensely enhanced and accelerated native extracellular matrix deposition, albeit not in an aligned fashion, even on the grooved substrates. Gene analysis at day 14 revealed that the 130 kPa grooved substrate without collagen type I coating and under macromolecular crowding conditions positively regulated human tenocyte phenotype. Collectively, this work illustrates the beneficial effects of combined physicochemical approaches in controlling cell fate during *in vitro* expansion.

## Graphical abstract

**Figure:**
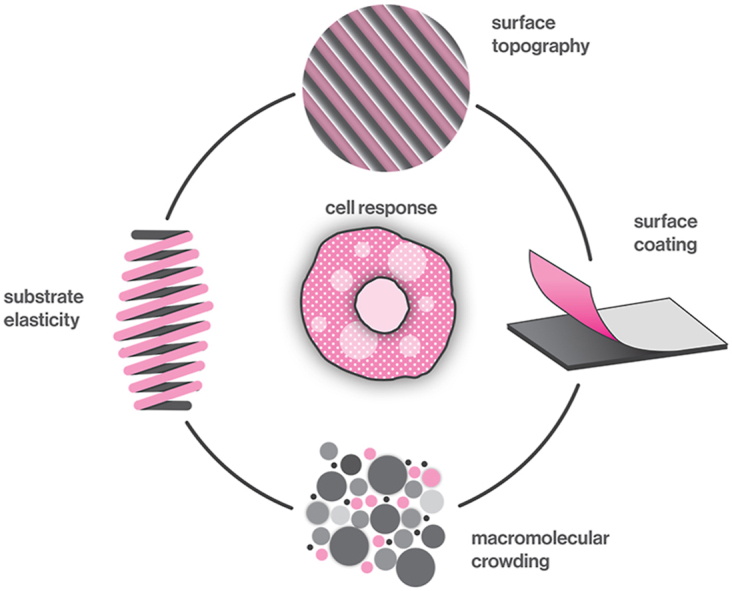

## 1 Introduction

During *in vitro* culture, bereft of their optimal tissue niche, cells age rapidly [1] and lose their phenotype, function and therapeutic potential [2]. This is a significant impediment in the development of cell therapies that require prolonged *ex vivo* culture periods to obtain sufficient number of viable and active cell populations [3]. Material design characteristics (e.g. mechanical properties [4], architectural features [5], surface chemistry [6]) and extracellular matrix (ECM, e.g. collagen, laminin, fibronectin presentation [7]) induced signals are favoured as a means to control cell fate *in vitro* for simplicity, scalability and acceptable levels of potency purposes. Considering, however, that cells reside in a complex *in vivo* milieu exposed simultaneously to numerous tissue-specific signals that collectively determine their phenotype and function, contemporary tissue engineering investigates combined stimuli strategies to control cell fate in culture [8,9]. In this frontier, the beneficial effects of combination approaches (e.g. surface topography and substrate rigidity [10], multiple ECM molecule presentation, either in the form of decellularised tissues [11] or cell layers [12]) in controlling *ex vivo* cell phenotype and function have been well-documented in the literature.

In tendon engineering, combination strategies continuously gaining pace [13], as tenocytes, the main resident cell population of tendon tissues and the preferred cell population for tendon engineering [14], readily lose their phenotype and function in traditional cell culture systems [15]. For example, substrate to tissue elasticity mismatch, even in the presence of topography, has been shown to result in tenocyte phenotype losses/*trans*-differentiation [16]. Tendon-derived matrices, as opposed to other tissue [17] or biomaterial [18] matrices, have been shown to maintain tenogenic phenotype, largely attributed to their tissue-specific multimolecular composition. Despite the relative simplicity of implementing material and ECM-based approaches in cell culture, in the quest of the optimal combination of *in vitro* microenvironment modulators to control tenogenic phenotype, a significant volume of work has been directed towards scaffolds with growth factors [19] or mechanical stimulation [20] and ECM-based approaches with growth factors [21] or mechanical stimulation [22]. Although data derived from such studies have significantly improved our knowledge in controlling tenocyte fate *ex vivo*, their inherent complexity (e.g. literally infinite number of growth factor and mechanical loading permutations) jeopardises scalability, commercialisation and, ultimately, clinical translation.

Discovery of appropriate combinations of scalable and potent in controlling cell fate *in vitro* microenvironment modulators may prove critical for the development of functional cell therapies. Thus, herein, we hypothesised that appropriate material (e.g. substrate rigidity, surface topography) and ECM (collagen type I coating, macromolecular crowding [MMC]) induced signals will maintain human tenocyte phenotype in culture. To validate our hypothesis, polydimethylsiloxane (PDMS) substrates with different rigidity, with/without anisotropic surface topography and with/without collagen type I coating were fabricated and used in human tenocyte cultures with/without MMC. Phenotype preservation was assessed via maintenance of physiological elongated cell morphology, basic cellular functions (i.e. viability, metabolic activity, cell number), protein synthesis and gene expression (i.e. maintenance and/or upregulation of most tenogenic genes and maintenance and/or downregulation of most chondrogenic and osteogenic genes).

## 2 Materials and methods

### 2.1 Materials

All chemicals, cell culture media and reagents were purchased from Sigma Aldrich (Ireland), unless otherwise stated. Tissue culture consumables were purchased from Sarstedt (Ireland) and Nunc (Denmark).

### 2.2 Substrate fabrication

A silicon wafer with groove dimensions of 2,000 nm (groove depth) × 2,000 nm (groove width) × 2,000 nm (line width) was fabricated by photolithography followed by reactive ion etching. Briefly, silicon wafers were spin-coated with a positive photoresist film (S1813 PR, Shipley) and then exposed with an OAI Mask Aligner (Model MBA800). Following photoresist development (MF 319 developer) at 20 °C chamber temperature for 8 min, the master mould was etched by reactive ion etching (Oxford ICP etcher) using CHF3 + SF6 ionised gas. Substrates of varying stiffness were prepared using commercially available PDMS elastomer kits Sylgard® 184 and Sylgard® 527 (Dow Corning, USA). Sylgard® 184 and Sylgard® 527 were prepared individually as per manufacturer's instructions and subsequently mixed at 1 to 0, 1 to 1, 1 to 5 and 0 to 1 ratios. A thermal imprinting technique was used to pattern the surface of the PDMS substrates. In brief, following the thorough mixing of the formulation ratios, the mixtures were poured over a silicon wafer to create grooved substrates and a glass petri dish to create planar substrates. The mixtures were degassed under vacuum for 20–40 min and then cured at 200 °C for 3–5 min, cooled and removed from template. Prior to collagen type I coating and cell culture, substrates were treated with oxygen plasma for 45 s at 100 W.

### 2.3 Dynamic mechanical analysis

The surface hardness of the various PDMS formulation ratios was determined using a Nanovea Nano Module nano-indenter with a stainless steel ball tip of 1.5 mm (Nanovea, USA). Measurements were taken at room temperature and testing was performed at a frequency of 2 Hz with maximum electromotive force of 1 mV, loading and unloading rate of 2 mV/min and creep time of 30 s. Following the ASTM E2546 (ISO 14577) instrumented indentation standards, the phase angle, storage modulus and loss modulus were determined using load/displacement curves. This measurement is based on the slope of the unloading curve, estimated contact area and indentation depth. Dynamic mechanical analysis (DMA) was performed in blind fashion by Nanovea®.

### 2.4 Atomic force microscopy analysis

Atomic force microscopy (AFM) analysis was performed with a commercial microscope (Veeco Dimension 3100 AFM, USA), using high aspect ratio silicon AR5-NCLR (force constant: 48 N/m; resonance frequency: 190 kHz; Nano Sensors™, Switzerland) cantilever. Imaging was performed with a scan rate of 0.5 Hz over an area of 10 μm^2^ in tapping mode. Images were processed using Gwyddion software (version 2.42, Czech Republic). The parameters groove depth, groove width and line width were used to describe the substrate surface topography of grooved samples and the parameter mean roughness (Ra) was used to describe the substrate surface topography of planar samples.

### 2.5 Brightfield microscopy and optical profilometry

Transfer of the imprint pattern from template to PDMS substrate was confirmed with bright-field microscopy (Leica DMIL LED inverted microscope, Germany) and a white-light interferometer (Zygo Newview 100 surface profiler, USA). The surface profile of the PDMS substrates was measured using optical profilometry (Zygo Newview 100 surface profiler white-light interferometer and Zygo Metropro® software, USA). A 20× objective was used to image the samples at a 2× zoom, 100 μm bipolar scans were taken with a measurement time of 54 s.

### 2.6 Cell culture

Human Achilles tendon tenocytes, from a healthy 29-year-old female donor, were purchased from DV Biologics (USA) and used experimentally at passages 2–5 (viability: passage 3; metabolic activity: passage 2; nuclear and cytoskeleton staining and analysis: passage 5; fibronectin staining: passage 2; all collagen staining: passage 3; focal adhesion kinase [FAK] analysis: passage 3; gene analysis: passage 3). The cells were cultured in Dulbecco's Modified Eagle's Medium with 4,500 mg/L glucose, l-glutamine, sodium pyruvate and sodium bicarbonate (D6429) supplemented with 10% foetal bovine serum (FBS) and 1% penicillin/streptomycin and were maintained at 37 °C in a humidified atmosphere of 5% CO_2_. About 0.25 mL of 0.5 mg/mL bovine collagen type I solution, which was extracted in-house [23,24], was used for the collagen type I coated groups. Following an overnight incubation with collagen type I, the groups were immediately washed with 70% ethanol and Hank's balanced salt solution (HBSS). Cells were seeded at a density of 25,000 cells/cm^2^. After 24 h, culture media were replaced with media containing 100 μM ascorbic acid phosphate (to induce collagen synthesis [[25], [26], [27], [28]]) and 100 μg/mL carrageenan (to induce ECM deposition [[29], [30], [31], [32], [33]]). The media were changed every 3–4 days. Biological analysis was conducted after 3, 7 and 14 days from the first MMC treatment.

### 2.7 Cell viability analysis

At each time point (3, 7 and 14 days), Live/Dead® assay (Invitrogen, Ireland) was performed to assess the influence of substrate stiffness, surface topography, collagen type I coating and MMC on cell viability, as per manufacturer's protocol. In brief, cells were washed with HBSS and a solution of 4 μM calcein AM and 2 μM ethidium homodimer I was added to each well. Cells were incubated at 37 °C and 5% CO_2_ for 30 min. Fluorescent images were captured using an Olympus IX-81 (Japan) inverted fluorescence microscope at 10× magnification.

### 2.8 Cell metabolic activity analysis

At each time point (3, 7 and 14 days), the alamarBlue® assay was used to evaluate the influence of substrate stiffness, surface topography, collagen type I coating and MMC on cell metabolic activity, as per manufacturer's instructions. In brief, cells were washed with HBSS and a 10% alamarBlue® solution in HBSS was added to each well. Cells were incubated in this solution at 37 °C and 5% CO_2_ for 4 h and absorbance was measured at 550 and 595 nm with a Varioskan Flash Spectral scanning multimode reader (Thermo Scientific, Ireland). Metabolic activity was expressed in terms of percentage reduction of the alamarBlue® dye and was normalised to the tissue culture plastic (TCP) control.

### 2.9 Cell number analysis

At days 3, 7 and 14, cells were fixed with 4% paraformaldehyde (PFA), permeabilised with 0.2% Triton X-100 and stained with 4′,6-diamidino-2-phenylindole (DAPI) to stain nuclei. Fluorescent images were captured using an Olympus IX-81 (Japan) inverted fluorescence microscope at 10× magnification. To assess cell number, one substrate per technical triplicate was imaged and five fields-of-view (FOVs) were taken from each substrate (in total, 15 images were analysed per experimental group) and the images were analysed using ImageJ software (NIH, USA).

### 2.10 Nuclear and cytoskeleton staining and nuclei morphometric analysis

At days 3, 7 and 14, cells were fixed with 4% PFA, permeabilised with 0.2% Triton X-100 and stained with DAPI to stain nuclei and rhodamine-labelled phalloidin to stain cell cytoskeleton. Fluorescent images were captured using an Olympus IX-81 (Japan) inverted fluorescence microscope at 10× magnification. To assess nuclear area and aspect ratio (major axis/minor axis), one substrate per technical triplicate was imaged and five FOVs were taken from each substrate (in total, 15 images analysed per experimental group) and the images were analysed using ImageJ software (NIH).

### 2.11 Immunocytochemistry analysis

At days 3, 7 and 14, cells were fixed in 4% PFA and blocked with 3% bovine serum albumin in phosphate buffered saline (PBS) for 30 min. Cells were then incubated with a primary antibody for 90 min, washed with PBS and subsequently incubated with secondary antibody for 30 min (Supplementary Table S1). Nuclei were counterstained with DAPI for 5 min. To determine deposited matrix area at each time point, fluorescent area per image was quantified using ImageJ software (NIH) and was normalised to cell number.

### 2.12 Fast Fourier transform analysis

To assess nuclei, cytoskeleton and deposited ECM orientation, one substrate per technical triplicate was imaged and five FOVs were taken from each substrate (in total, 15 images were analysed per experimental group). Eight-bit grey-scale images of stained nuclei, cytoskeleton and deposited ECM, captured using an Olympus IX-81 inverted fluorescent microscope (Japan), were processed with the fast Fourier transform (FFT) function of the ImageJ (NIH) software. FFT images were rotated 90° right and radial intensity summing was performed and plotted in relation to angle of acquisition using the ImageJ plug-in ‘Oval Profile’.

### 2.13 Focal adhesion kinase analysis

At day 3, FAK was measured by the *in vitro* SimpleStep ELISA® kit as per manufacturer's instructions (Abcam, UK). In brief, FAK standards (0–100 pg/mL) and samples were prepared and added to the appropriate wells of the pre-coated 96-well ELISA plate strips provided. The detection antibody was then added and incubated for 3 h at room temperature on a plate shaker at 400 rpm. Plates were washed and the 3,3′,5,5′-tetramethylbenzidine substrate solution was added to each well and incubated for 10 min in the dark on a plate shaker at 400 rpm. Stop solution was then added to each well and absorbance was measured at 450 nm with a Varioskan Flash Spectral scanning multimode reader (Thermo Scientific).

### 2.14 Gene expression analysis

Gene expression analysis was conducted using a TaqMan® RealTime ready Custom Panel for RT-qPCR (Roche, Ireland) to assess expression of tenogenic, chondrogenic and osteogenic markers (Supplementary Table S2). Briefly, total RNA was extracted at days 7 and 14 using TRI Reagent® (Roche, Ireland) for 5 min at room temperature to disrupt cell membranes. The TRI Reagent® was collected, chloroform was added and the solution was then vortexed for 15 s followed by incubation at room temperature for 5 min. The solution was centrifuged and the upper aqueous phase containing the RNA was collected and mixed with ethanol, after which the solution was purified using the High Pure RNA isolation kit (Roche). Total RNA concentration and quality were analysed using the NanoDrop 1,000 (Thermo Scientific) and the Agilent 2100 Bioanalyser (Agilent Technologies, Ireland). Samples with an RNA integrity value of 8 or above were used for further analysis. RNA was transcribed to cDNA using a Transcriptor First Strand cDNA synthesis kit (Roche) and 1 μg of RNA sample was used for all conditions. The thermal block cycle was set at 50 °C for 1 h and at 85 °C for 5 min for enzyme inactivation. In total, 1 μL of transcribed cDNA was added to 9 μL of probes master in a RealTime ready custom 384-well plate (Roche). Negative controls of empty wells and untranscribed RNA were utilised and plates were run in the LightCycler® 480 Instrument (Roche). For analysis, using the 2-ΔCt method, mean Ct values of each target gene were normalised to the housekeeping gene values. To analyse the changes in gene expression between the TCP control and all other conditions for each day, 2-ΔΔCt method was used. Z-scores of fold changes were calculated and relevant up- and down-regulations were accepted when the score was at least two standard deviations away from the mean value of fold-change for each gene.

### 2.15 Statistical analysis

Experiments were conducted in three technical replicates and data are expressed as mean ± standard deviation. Statistical analysis was performed using SPSS® (version 24, IBM Corp., USA). Multifactorial analysis of variance (*F*-test) was used for multiple comparisons and a Student–Newman–Keuls post hoc test was used for pairwise comparisons after confirming the samples followed a normal distribution (Kolmogorov–Smirnov test) and had equal variances (Bartlett's and Levene's test for homogeneity of variances). When any of these assumptions were violated, non-parametric tests were used for multiple comparisons (Kruskal–Wallis test/*K*-test) and pairwise comparisons (Mann–Whitney *U*-test/*U*-test). Statistical significance was accepted at *p*<0.05.

## 3 Results

### 3.1 Scaffold mechanical and surface characterisation

**Fig. 1:**
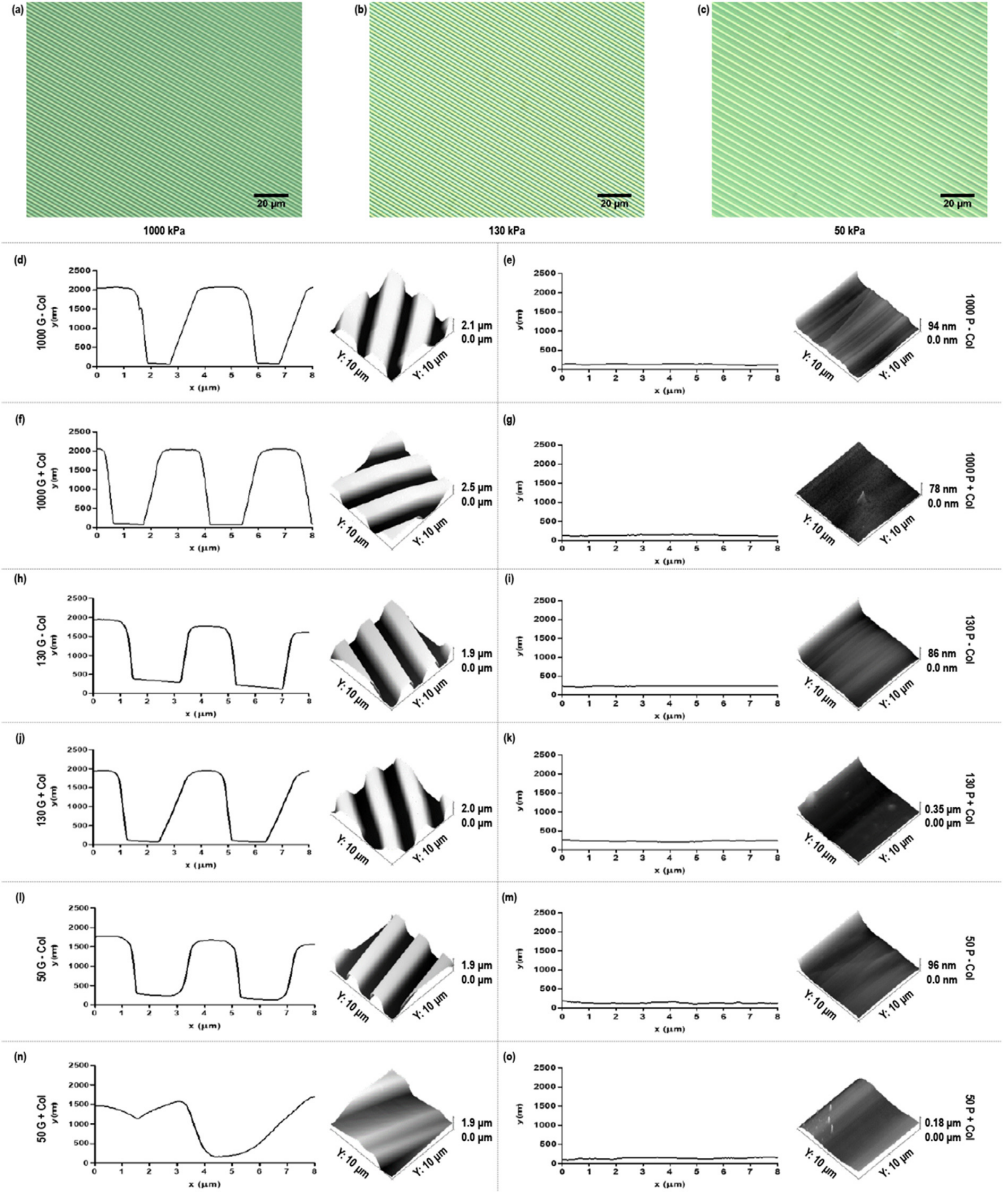
Brightfield micrographs of the 1,000 kPa (a), 130 kPa (b) and 50 kPa (c) grooved PDMS substrates (scale bar = 20 μm). AFM-derived line profiles of the 1,000 kPa grooved (d, f) and planar (e, g) substrates, 130 kPa grooved (h, j) and planar (i, k) substrates, 50 kPa grooved (l, n) and planar (m, o) substrates with (f, g, j, k, n, o) and without (d, e, h, i, l, m) collagen type I coating, generated using Gwyddion software.

DMA revealed that substrates with storage modulus of approximately 1,000 kPa (Sylgard® 184), 130 kPa (1:1 ratio of Sylgard® 184 to Sylgard® 527), 50 kPa (1:5 ratio of Sylgard® 184 to Sylgard® 527) and 4 kPa (0:1 ratio of Sylgard® 184 to Sylgard® 527) were produced (Supplementary Table S3). Brightfield microscopy and AFM analyses showed that all the substrates retained the topographical features of the silicon wafer, except those with 4 kPa storage modulus, and coating the substrates with collagen type I caused the grooves of 50 kPa substrates to stick together (Fig. 1). Groove depth was confirmed at ∼2,000, ∼1,900 and ∼1,850 nm for the 1,000, 130 and 50 kPa substrates, respectively (Supplementary Table S4). Groove width was significantly (*p*<0.05) higher and line width was significantly (*p*<0.05) lower on 1,000 kPa substrates than on 130 and 50 substrates (Supplementary Table S4). No statistically significant (*p*>0.05) difference in surface roughness of the planar 1,000, 130 and 50 kPa substrates was detected due to substrate stiffness or collagen coating (Supplementary Table S4). Following coating of the grooved PDMS substrates with 0.5 mg/mL collagen type I, AFM analysis showed that the 1,000 kPa substrate retained (*p*>0.05) the dimensionality of the grooves; the 130 kPa substrate retained (*p*>0.05) the groove depth, but the groove width was increased (*p*<0.05) and the line width was decreased (*p*<0.05); and the groove width was increased (*p*<0.05) and the groove depth and the line width were decreased (*p*<0.05) of the 50 kPa substrate (Supplementary Table S4).

### 3.2 Cell morphometric and FAK analyses

**Fig. 2:**
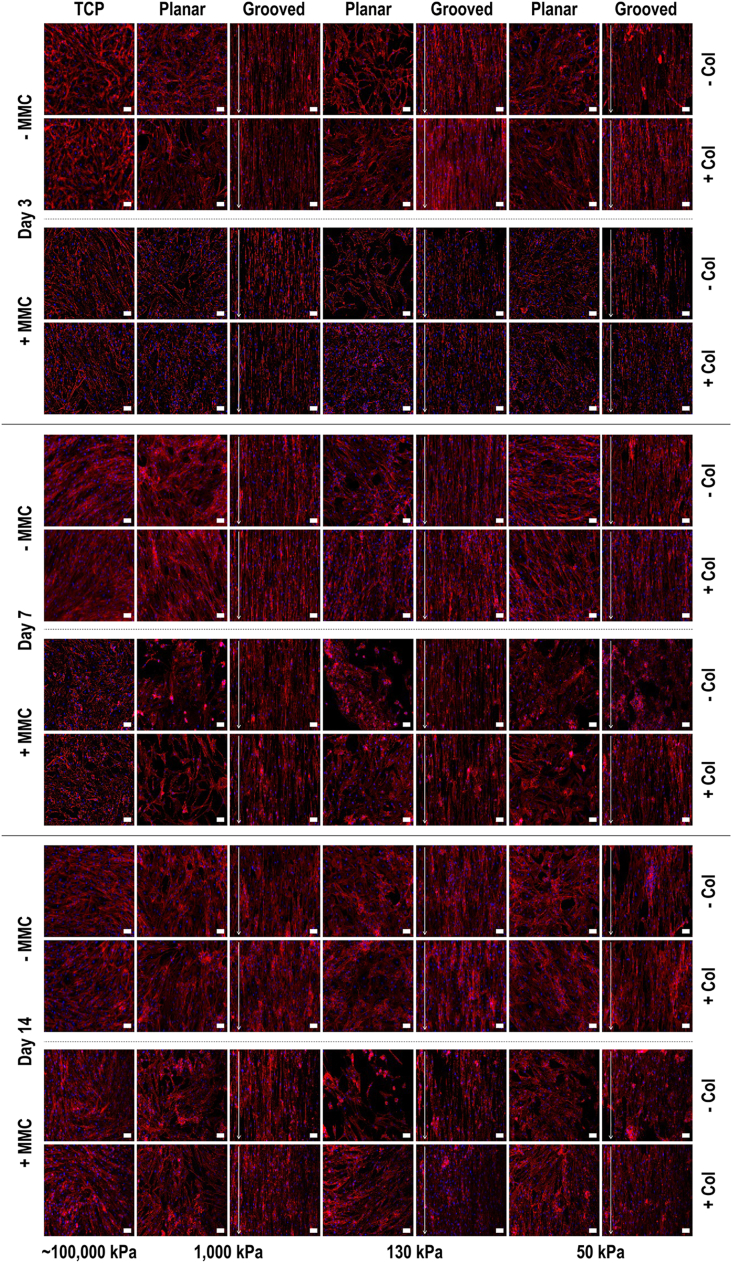
Human tenocyte morphology at days 3, 7 and 14 on tissue culture plastic (TCP) without and with collagen type I coating (− Col, + Col) and macromolecular crowding (− MMC, + MMC) and on substrates of varying stiffness (1,000 kPa, 130 kPa, 50 kPa), surface topography (planar, grooved), collagen type I coating (− Col, + Col) and macromolecular crowding (− MMC, + MMC), as shown using rhodamine phalloidin and DAPI staining. Human tenocytes oriented randomly on planar surfaces and aligned parallel to the grooves of the grooved surfaces. White arrow indicates direction of surface topography. Scale bar = 50 μm.

Qualitative rhodamine phalloidin and DAPI staining analysis (Fig. 2) revealed that the cells attached and grew homogeneously on the substrates, although clusters and cell-free areas were noted on the softest substrates. In general, quantitative cytoskeleton (Supplementary Figs. S1–S3) and nuclei (Supplementary Figs. S4–S6) orientation analyses at days 3, 7 and 14 revealed cellular orientation parallel to the orientation of the grooves. In general, cells seeded on TCP had larger (*p*<0.05) nuclei area than cells on any PDMS substrate; in comparison to smooth substrates, the cells exhibited smaller (*p*<0.05) nuclear area on grooved substrates at all time points; collagen type I coating resulted in larger (*p*<0.05) nuclear area at all time points; and MMC decreased (*p*<0.05) at day 3, increased (*p*<0.05) at day 7 and did not change (*p*>0.05) at day 14 nuclei area (Supplementary Fig. S7). In general, aspect ratio was higher (*p*<0.05) on cells cultured on PDMS than on TCP; in comparison to smooth substrates, the cells exhibited higher (*p*<0.05) aspect ratio on grooved substrates at all time points; collagen type I coating resulted in smaller (*p*<0.05) aspect ratio; and MMC increased (*p*<0.05) nuclei aspect ratio only at day 14 (Supplementary Fig. S8). FAK levels were higher (*p*<0.05) on TCP than on PDMS groups, higher (*p*<0.05) when cells were cultured in the presence of collagen type I coating and lower (*p*<0.05) in the presence of MMC (Supplementary Fig. S9).

### 3.3 Basic cellular function analysis

In general, at a given time point (day 3, 7 and 14), cell number (Supplementary Fig. S10) and metabolic activity (Supplementary Fig. S11) were increased (*p*<0.05) when cells were cultured on TCP than on PDMS substrates, were increased (*p*<0.05) when cells were cultured on collagen type I coated substrates and were decreased (*p*<0.05) when MMC was used. Cell viability (Supplementary Fig. S12) was not affected by substrate stiffness, surface topography, collagen type I coating, MMC or time in culture (>95% for all treatments).

### 3.4 Protein analysis

**Fig. 3:**
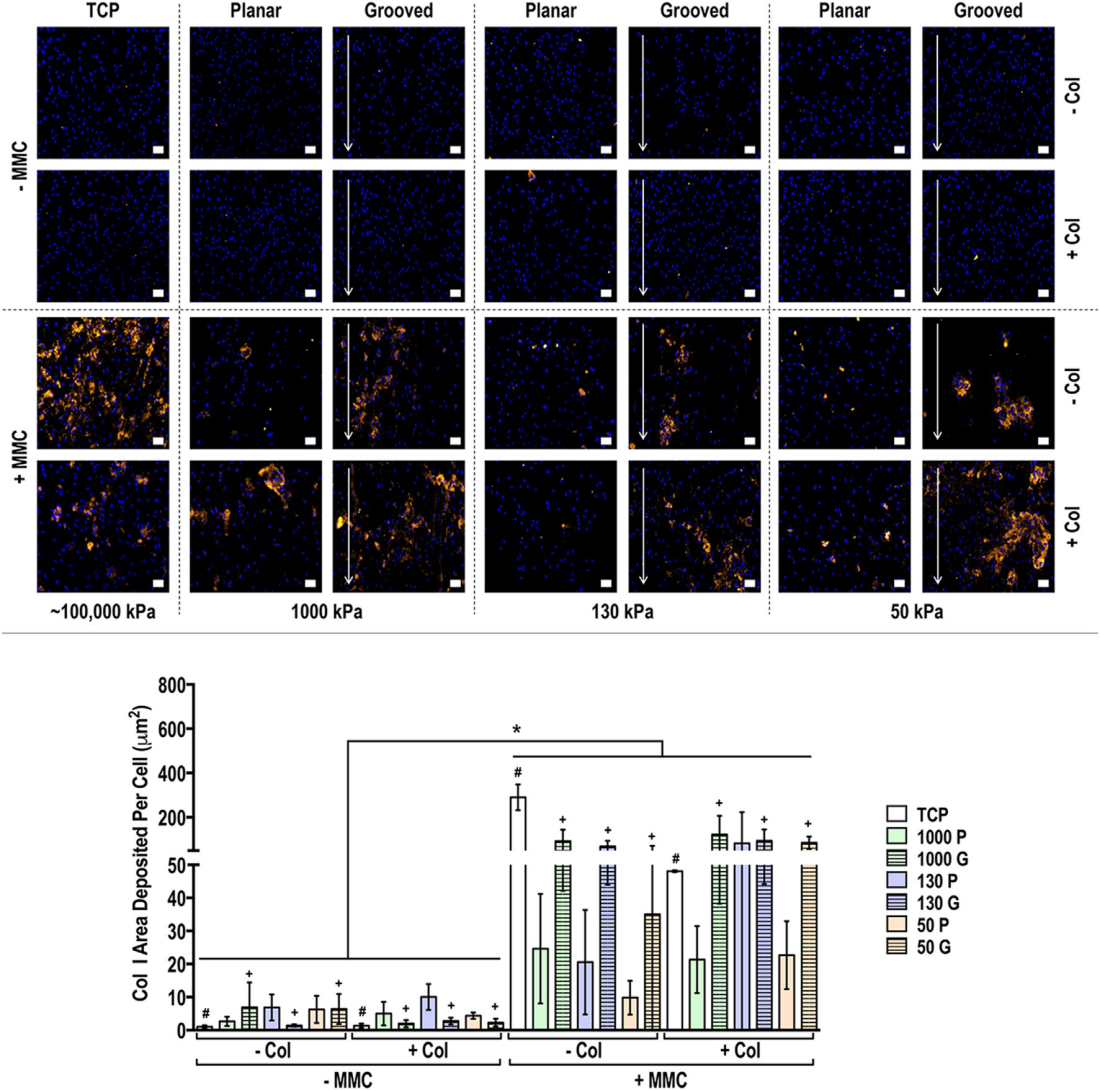
Human tenocyte deposited collagen type I matrix and quantification of collagen type I matrix area deposited per cell at day 14 on tissue culture plastic (TCP) without and with collagen type I coating (− Col, + Col) and macromolecular crowding (− MMC, + MMC) and on substrates of varying stiffness (1,000 kPa, 130 kPa, 50 kPa), surface topography [planar (P), grooved (G)], collagen type I coating (− Col, + Col) and macromolecular crowding (− MMC, + MMC). Collagen type I is represented in orange. DAPI is presented in blue. Scale bar = 50 μm. ∗ Indicates statistically significant difference (*p*<0.05) between without and with collagen type I coating and between without and with MMC, # indicates statistical difference (*p*<0.05) between TCP and PDMS substrates and + indicates statistical difference (*p*<0.05) between planar and grooved topography. The most striking result was that MMC significantly increased (*p*<0.05) collagen type I deposition in a globular fashion, independently of the substrate stiffness, surface topography and collagen type I coating.

**Fig. 4:**
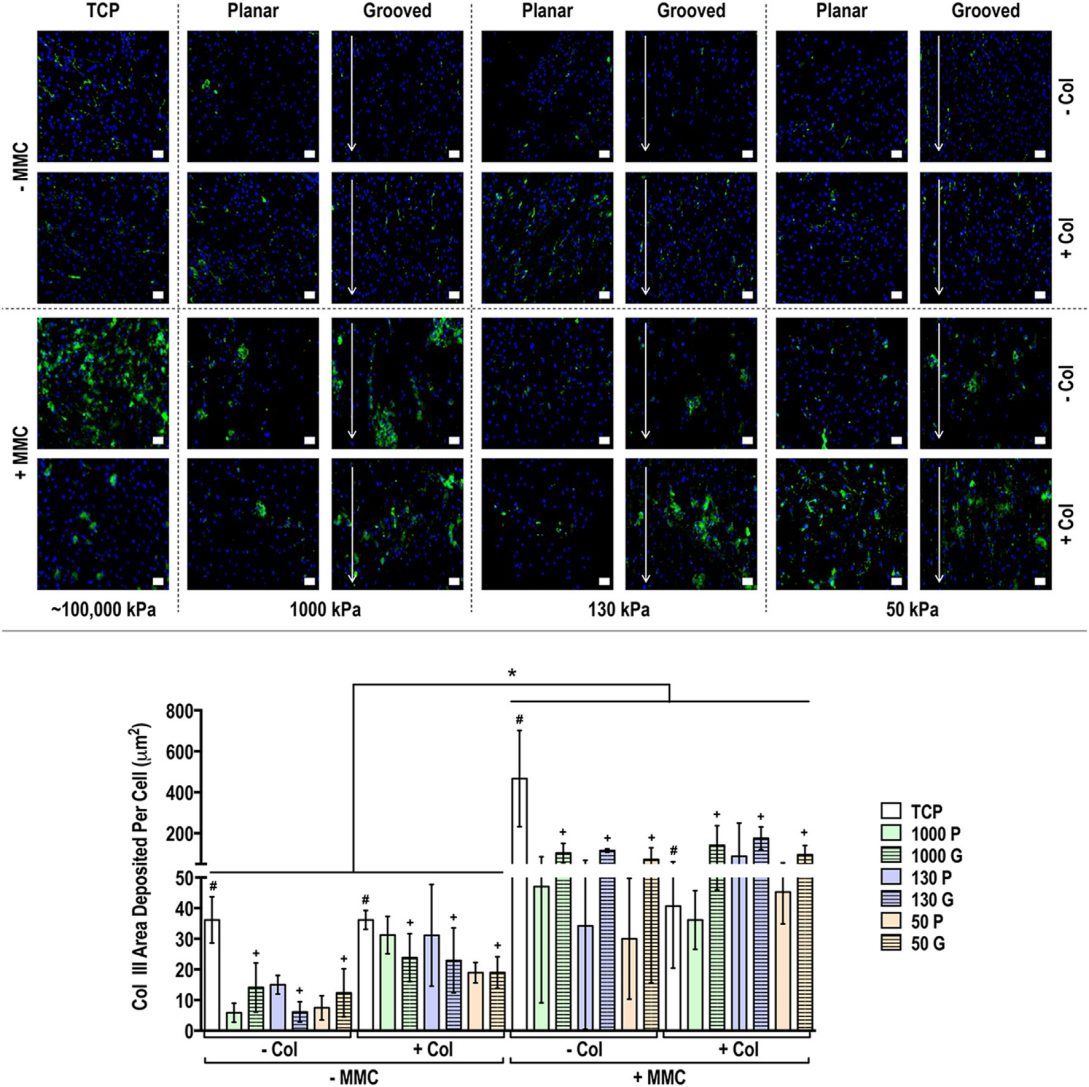
Human tenocyte deposited collagen type III matrix and quantification of collagen type III matrix area deposited per cell at day 14 on tissue culture plastic (TCP) without and with collagen type I coating (− Col, + Col) and macromolecular crowding (− MMC, + MMC) and on substrates of varying stiffness (1,000 kPa, 130 kPa, 50 kPa), surface topography [planar (P), grooved (G)], collagen type I coating (− Col, + Col) and macromolecular crowding (− MMC, + MMC). Collagen type III is represented in green. DAPI is represented in blue. Scale bar = 50 μm. ∗ Indicates statistically significant difference (*p*<0.05) between without and with collagen type I coating and between without and with MMC, # indicates statistical difference (*p*<0.05) between TCP and PDMS substrates and + indicates statistical difference (*p*<0.05) between planar and grooved topography. The most striking result was that MMC significantly increased (*p*<0.05) collagen type III deposition in a globular fashion, independently of the substrate stiffness, surface topography and collagen type I coating.

**Fig. 5:**
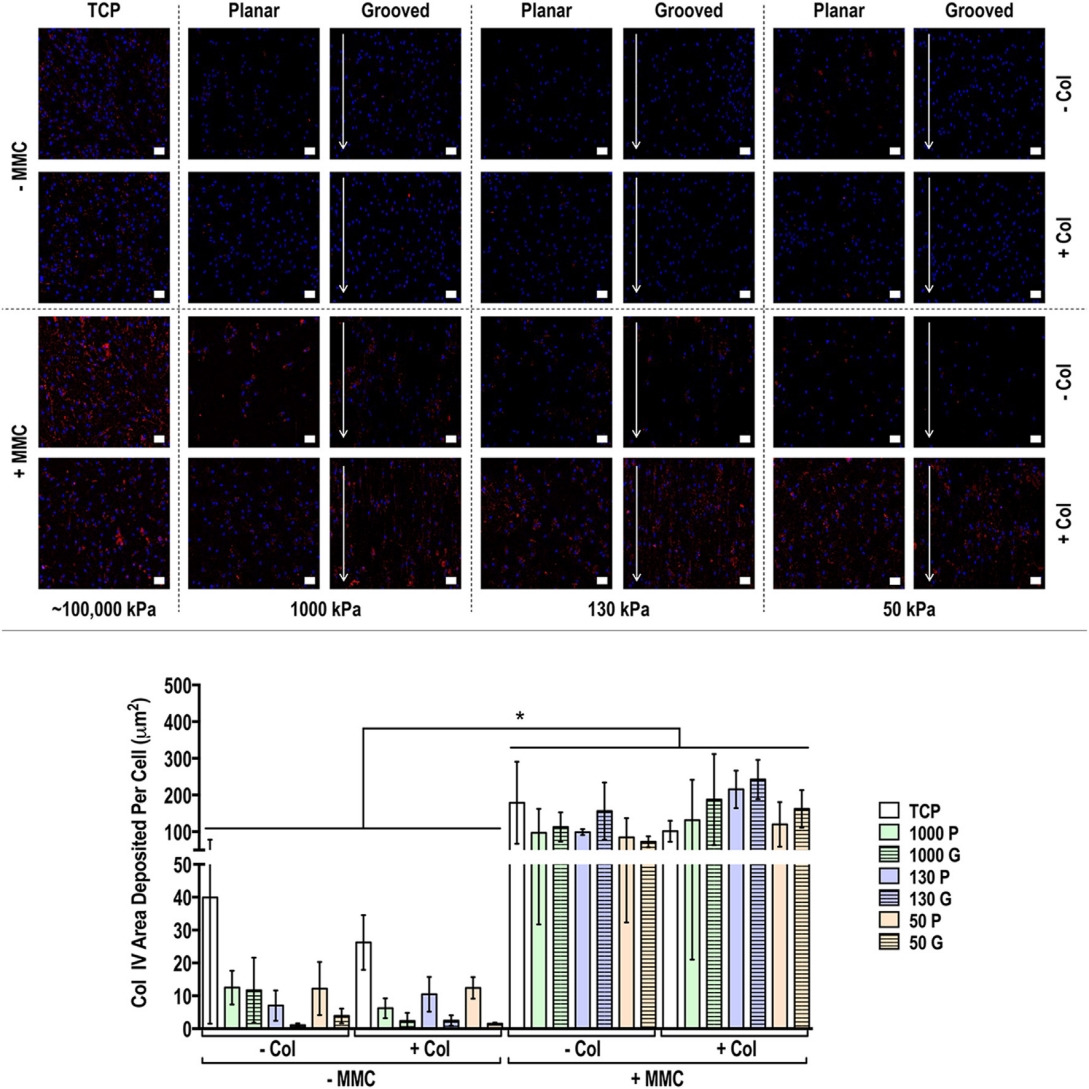
Human tenocyte deposited collagen type IV matrix and quantification of collagen type IV matrix area deposited per cell at day 14 on tissue culture plastic (TCP) without and with collagen type I coating (− Col, + Col) and macromolecular crowding (− MMC, + MMC) and on substrates of varying stiffness (1,000 kPa, 130 kPa, 50 kPa), surface topography [planar (P), grooved (G)], collagen type I coating (− Col, + Col) and macromolecular crowding (− MMC, + MMC). Collagen type IV is represented in red. DAPI is presented in blue. Scale bar = 50 μm. ∗ Indicates statistically significant difference (*p*<0.05) between without and with collagen type I coating and between without and with MMC, # indicates statistical difference (*p*<0.05) between TCP and PDMS substrates and + indicates statistical difference (*p*<0.05) between planar and grooved topography. The most striking result was that MMC significantly increased (*p*<0.05) collagen type IV deposition in a globular fashion, independently of the substrate stiffness, surface topography and collagen type I coating.

**Fig. 6:**
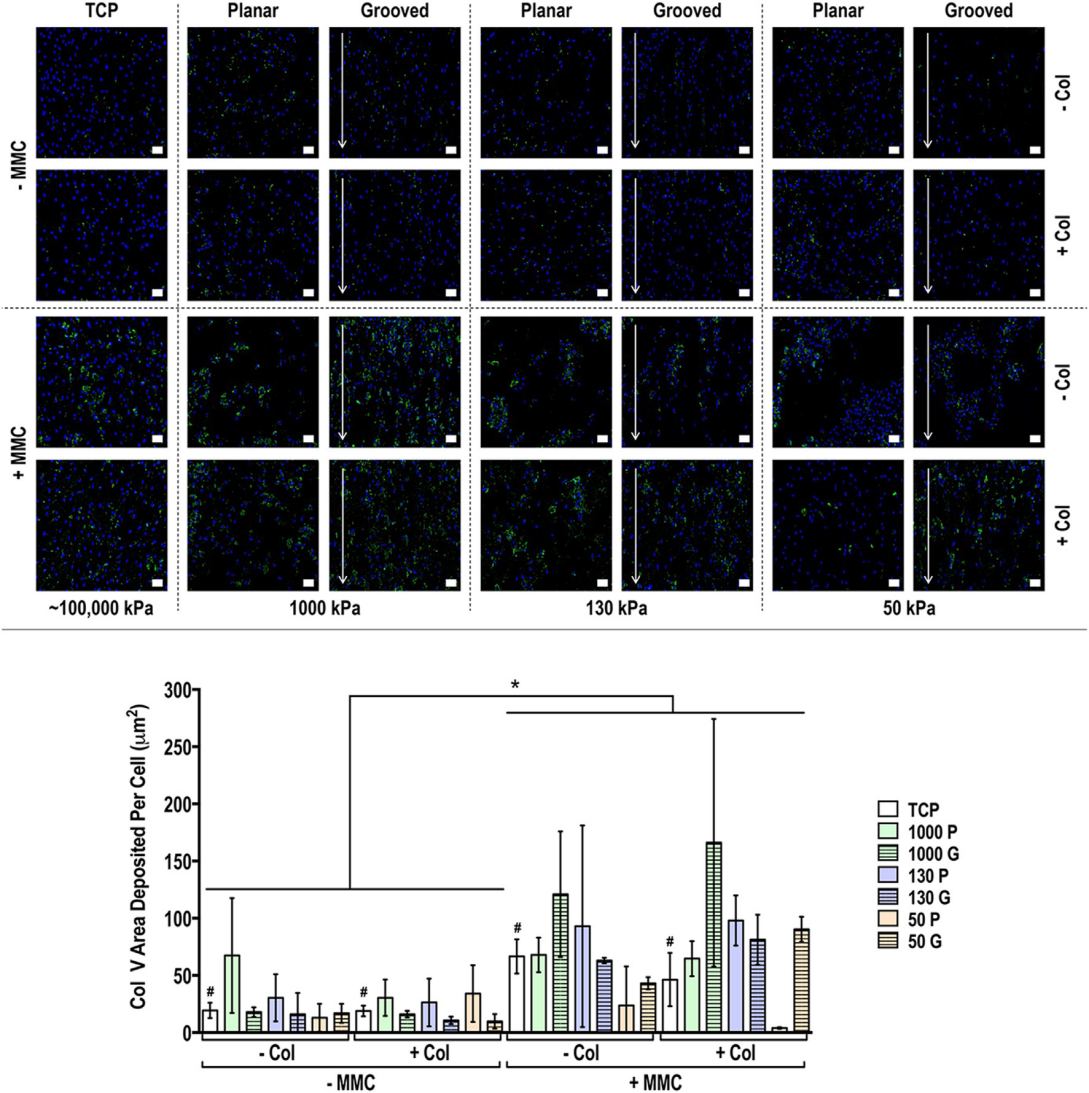
Human tenocyte deposited collagen type V matrix and quantification of collagen type V matrix area deposited per cell at day 14 on tissue culture plastic (TCP) without and with collagen type I coating (− Col, + Col) and macromolecular crowding (− MMC, + MMC) and on substrates of varying stiffness (1,000 kPa, 130 kPa, 50 kPa), surface topography [planar (P), grooved (G)], collagen type I coating (− Col, + Col) and macromolecular crowding (− MMC, + MMC). Collagen type V is presented in green. DAPI is presented in blue. Scale bar = 50 μm. ∗ Indicates statistically significant difference (*p*<0.05) between without and with collagen type I coating and between without and with MMC, # indicates statistical difference (*p*<0.05) between TCP and PDMS substrates and + indicates statistical difference (*p*<0.05) between planar and grooved topography. The most striking result was that MMC significantly increased (*p*<0.05) collagen type V deposition in a globular fashion, independently of the substrate stiffness, surface topography and collagen type I coating.

**Fig. 7:**
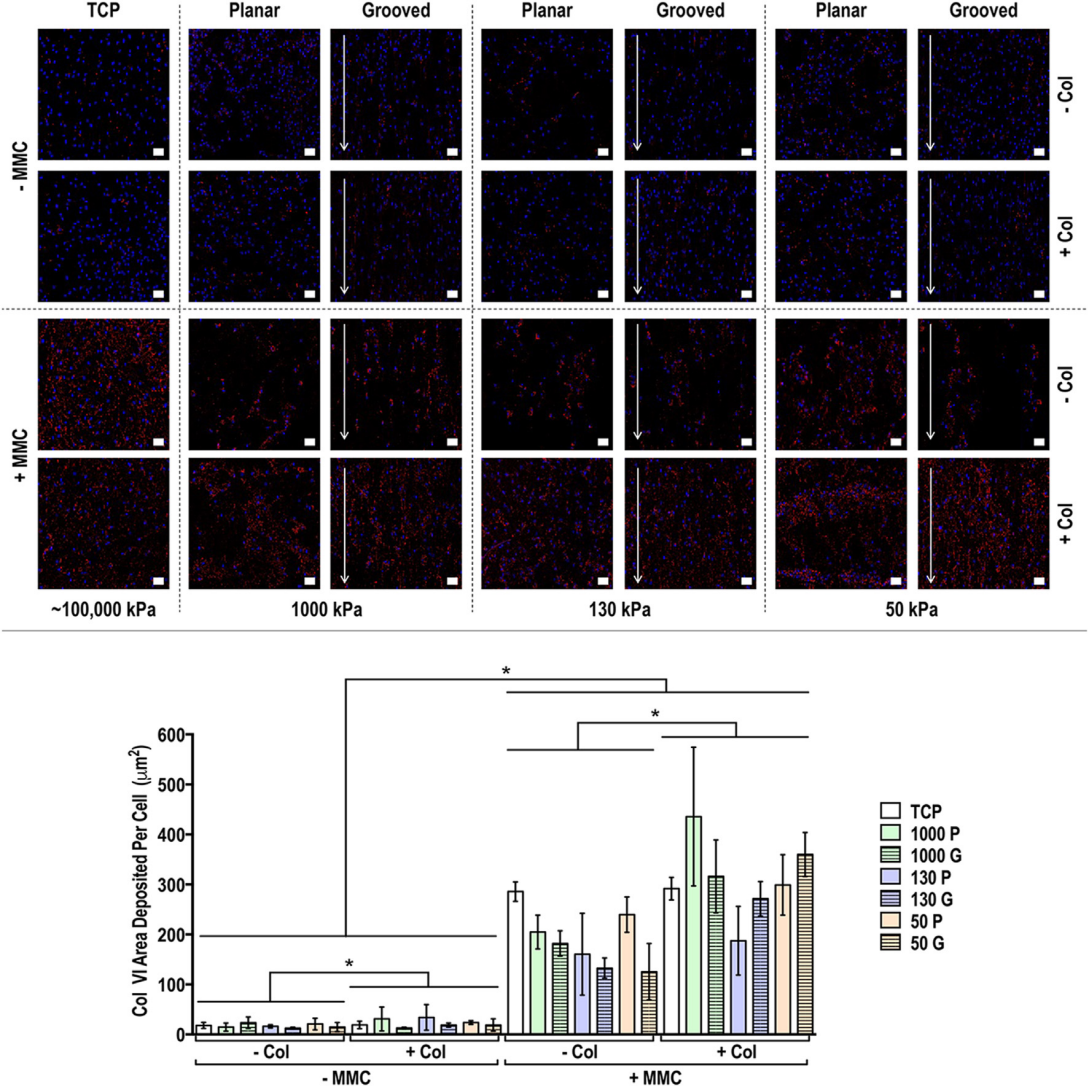
Human tenocyte deposited collagen type VI matrix and quantification of collagen type VI matrix area deposited per cell at day 14 on tissue culture plastic (TCP) without and with collagen type I coating (− Col, + Col) and macromolecular crowding (− MMC, + MMC) and on substrates of varying stiffness (1,000 kPa, 130 kPa, 50 kPa), surface topography [planar (P), grooved (G)], collagen type I coating (− Col, + Col) and macromolecular crowding (− MMC, + MMC). Collagen type VI is represented in red. DAPI is represented in blue. Scale bar = 50 μm. ∗ Indicates statistically significant difference (*p*<0.05) between without and with collagen type I coating and between without and with MMC, # indicates statistical difference (*p*<0.05) between TCP and PDMS substrates and + indicates statistical difference (*p*<0.05) between planar and grooved topography. The most striking result was that MMC significantly increased (*p*<0.05) collagen type VI deposition in a globular fashion, independently of the substrate stiffness, surface topography and collagen type I coating.

**Fig. 8:**
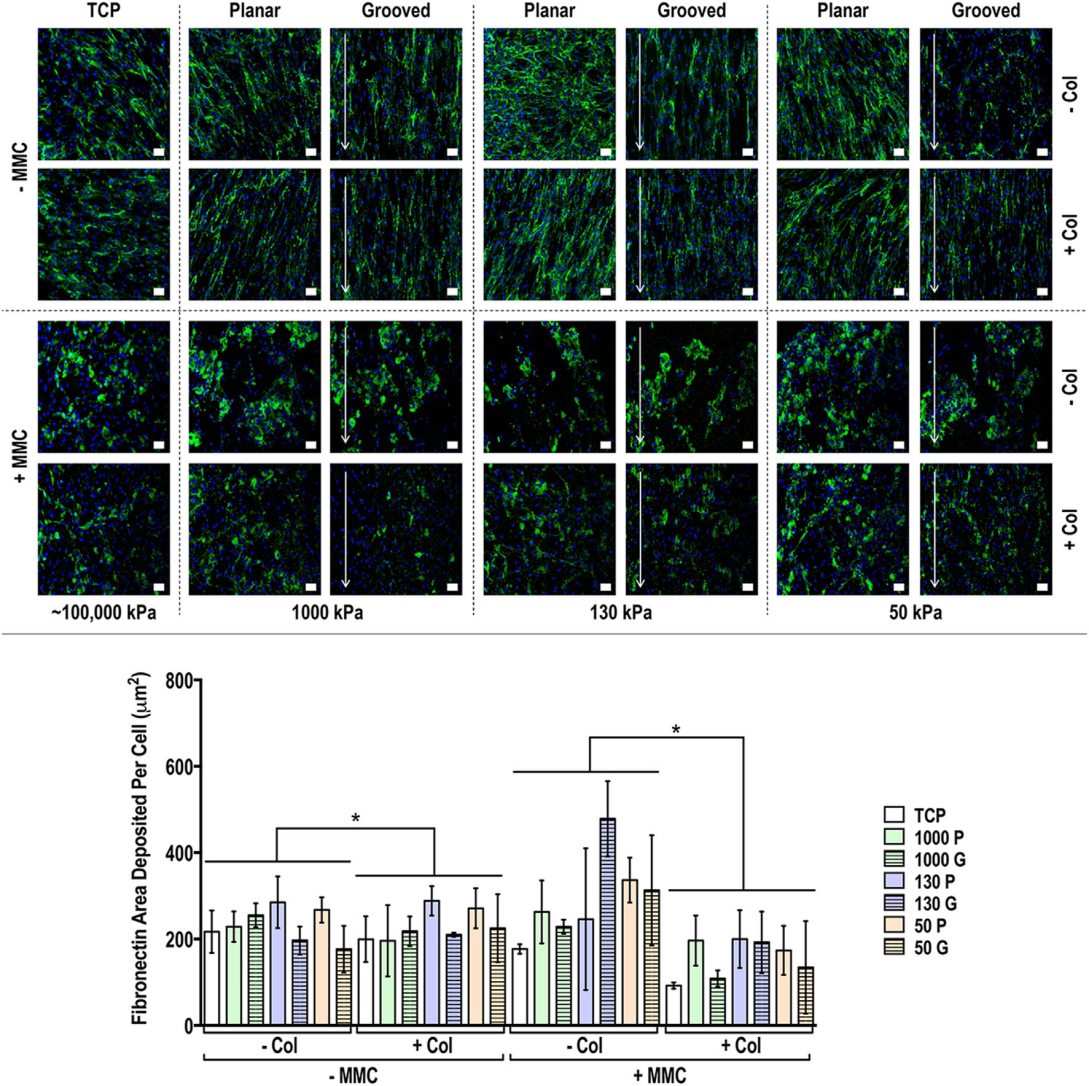
Human tenocyte deposited fibronectin matrix and quantification of fibronectin matrix area deposited per cell at day 14 on tissue culture plastic (TCP) without and with collagen type I coating (− Col, + Col) and macromolecular crowding (− MMC, + MMC) and on substrates of varying stiffness (1,000 kPa, 130 kPa, 50 kPa), surface topography [planar (P), grooved (G)], collagen type I coating (− Col, + Col) and macromolecular crowding (− MMC, + MMC). Fibronectin is represented in green. DAPI is represented in blue. Scale bar = 50 μm. ∗ Indicates statistically significant difference (*p*<0.05) between without and with collagen type I coating and between without and with MMC, # indicates statistical difference (*p*<0.05) between TCP and PDMS substrates and + indicates statistical difference (*p*<0.05) between planar and grooved topography. In the absence of MMC, anisotropic substrates induced bidirectional fibronectin deposition, independently of substrate rigidity and collagen type I coating, while in the presence of MMC, fibronectin was deposited in a globular manner, independently of surface topography, substrate rigidity and collagen type I coating.

Immunocytochemistry and quantification of deposited matrix area analyses of collagen types I, III, IV, V and VI and fibronectin at day 3 (Supplementary Figs. S13-S18), 7 (Supplementary Figs. S19-S24) and 14 (Fig. 3, Fig. 4, Fig. 5, Fig. 6, Fig. 7, Fig. 8) revealed that although collagen type I coating induced some differences at day 3 and day 7, its contribution at day 14 was minimal [in general, only increased (*p*<0.05) collagen type VI and decreased (*p*<0.05) fibronectin area deposited per cell]. At day 3, MMC increased (*p*<0.05) collagen types I, III, IV and VI, did not affect (*p*>0.05) collagen type V and decreased (*p*<0.05) fibronectin deposition; at day 7, MMC increased (*p*<0.05) collagen types I, III, IV, V and VI and decreased (*p*<0.05) fibronectin deposition; and at day 14, MMC increased (*p*<0.05) collagen types I, III, IV, V and VI and did not affect (*p*>0.05) fibronectin deposition. With respect to deposited ECM morphology (Supplementary Figs. S25–S30), in the absence of MMC on the grooved substrates, the deposited ECM had an aligned, parallel to the groove orientation, morphology, while in the presence of MMC on the grooved substrates, the deposited ECM had a globular morphology. Neither the rigidity nor the collagen coating affected deposited ECM morphology.

### 3.5 Gene analysis

**Fig. 9:**
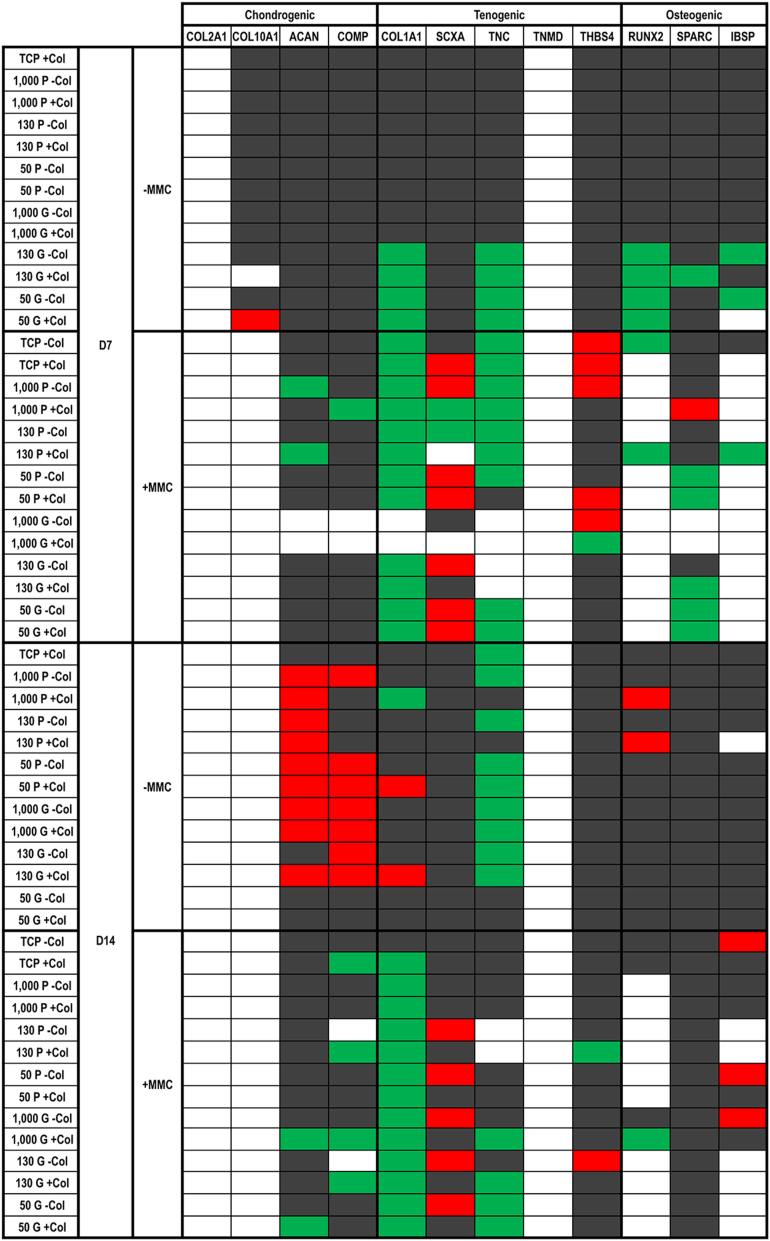
TaqMan® array showing human tenocyte expression of chondrogenic, tenogenic and osteogenic genes on tissue culture plastic (TCP) without and with collagen type I coating (− Col, + Col) and macromolecular crowding (− MMC, + MMC) and on substrates of varying stiffness (1,000 kPa, 130 kPa, 50 kPa), surface topography [planar (P), grooved (G)], collagen type I coating (− Col, + Col) and macromolecular crowding (− MMC, + MMC) at day 7 and day 14. Data are expressed in relation to TCP without collagen type I coating (− Col) and without macromolecular crowding (− MMC) at a given timepoint. The most important observations are that in the absence of MMC, by day 14, the 50 kPa grooved substrate (without/with collagen type I coating) did not affect the expression of chondrogenic, tenogenic and osteogenic markers and, in the presence of MMC, the 130 kPa grooved substrate without collagen type I upregulated SCXA and THBS4 and only downregulated COL1A1. White background, not detectable; grey background, unchanged; green background, downregulated two-fold; red background, upregulated two-fold.

Gene expression analysis (Fig. 9) at day 7 in the absence of MMC revealed that cells on all planar substrates maintained the expression of all chondrogenic, tenogenic and osteogenic markers, independently of the absence or presence of collagen type I coating, while on the grooved substrates, the 1,000 kPa substrate maintained the expression of all chondrogenic, tenogenic and osteogenic markers, independently of the absence or presence of collagen type I coating; and the 130 and 50 kPa substrates downregulated the expression of two tenogenic (COL1A1, TNC) and two osteogenic (RUNX2 and SPARC or IBSP) markers (the COL1A1, TNC and RUNX2, independently of the absence or presence of collagen type I coating; the IBSP in the absence of collagen type I coating). In the presence of MMC, among the planar substrates, the 50 kPa with collagen type I coating upregulated the most (SCXA, THBS4) and downregulated the least (COL1A1) tenogenic markers and downregulated the most (SPARC) osteogenic markers; and among the grooved substrates, the 130 kPa without collagen type I coating upregulated SCXA and only downregulated COL1A1.

Gene expression analysis (Fig. 9) at day 14 in the absence of MMC revealed that among the planar surfaces, only the TCP with collagen type I coating downregulated one tenogenic (TNC) marker; and among the grooved substrates, the 50 kPa substrate maintained the expression of all chondrogenic, tenogenic and osteogenic markers, independently of the absence or presence of collagen type I coating. In the presence of MMC, among the planar surfaces, the 130 kPa without collagen coating upregulated SCXA and only downregulated COL1A1 and among the grooved substrates, again the 130 kPa substrate without collagen coating upregulated SCXA and THBS4 and only downregulated COL1A1.

## 4 Discussion

*In vivo*, a continuous dynamic reciprocity between cells and ECM [34] orchestrates a sequence of spatiotemporal biological, biochemical and biophysical signalling cascades [35] that collectively regulate cellular phenotype and function. Following the nature's paradigm, contemporary regenerative medicine also employs combination approaches to control cell fate during the development of tissue engineered medicines. In the quest of the appropriate combinations of scalable and potent in controlling cell fate *in vitro* microenvironment modulators, herein, we ventured to assess the potential of material and ECM-induced signals (i.e. substrate rigidity, without/with anisotropic surface topography, without/with collagen coating and without/with MMC) to maintain human tenocyte phenotype in culture. We recognise that serum is often associated with phenotype losses [36] and *trans*-differentiation [37] in tenocyte cultures. Considering though the absence of commercially available serum-free and the complexity of published low [38] or no [39] serum tenocyte phenotype maintaining media, serum containing media were used herein, which constitute (for simplicity as opposed to efficiency) the state-of-the-art in the field. We also recognise that serum containing fibronectin may have influenced cellular and ECM morphology, considering that fibronectin localisation and fibrillisation are serum dependent [40]. With respect to cellular morphology, it has been shown that at >3% serum, fibronectin depletion has no effect on the rates of cellular spreading, while at <3% serum, fibronectin depletion delays, without prohibiting, cellular spreading [41]. With respect to ECM, it has been shown that serum fibronectin is incorporated into the same structures in the ECM as the cell-derived fibronectin [42] and the initial (within 60 min) globular appearance is replaced by a fibrillar pattern within 2–8 h [43]. Considering that all groups were subjected to the same serum, same serum concentration and for the same period of time and that our first readout time point was at day 4 (way after the time frame, i.e. min to h, that serum fibronectin may have an effect), we consider proportional and/or minimal, respectively, effect of serum fibronectin on our observations.

### 4.1 Scaffold mechanical and surface characterisation

PDMS substrates with storage modulus from 1,000 to 4 kPa were fabricated. This range of stiffness encompasses the majority of tissues in the body, including brain (0.6–2.7 kPa [44,45]), skin (10 kPa–161 MPa [[46], [47], [48], [49]]) tendon (400 kPa–1.2 GPa [[50], [51], [52]]), cartilage (400–950 kPa [[53], [54], [55], [56]]) and bone (40 kPa–24 GPa [7,[57], [58], [59]]). Brightfield microscopy confirmed an overall clean pattern transfer from the silicon template to the surface of the PDMS substrates and AFM provided a comprehensive analysis of surface parameters, albeit across a much smaller area. Substrates of 4 kPa storage modulus did not retain the topographical features of the silicon wafer and were not further used, while all other substrates exhibited uniform groove depth, line and width, all essential for groove-related cellular alignment [[60], [61], [62], [63], [64]]. After collagen type I coating, the groove width and the line width of the stiff (1,000 kPa) substrate were not affected, while the groove width and line width of the softer (130 and 50 kPa) substrates were increased and decreased, respectively. We attribute this deformation to collagen dehydration during AFM analysis [65] that resulted in shrinkage of line width and enlargement of groove width, via pulling the ridges together, of the soft substrates (130 and 50 kPa), while the rigid substrate (1,000 kPa) was able to maintain its topographical features. This dehydration during AFM analysis, as opposed to a permanent deformation, theory is supported by the fact that subsequent cell morphometric analysis revealed that all grooved substrates with collagen type I coating were able to induce cellular alignment in the direction of the grooves, albeit with variable efficiency (see Section 4.2).

### 4.2 Cell morphometric analysis

Rhodamine phalloidin and DAPI staining revealed that in general the cells attached and grew on all substrates. It is worth noting that on the 50 kPa substrates, cell-free areas and cell clusters were observed (collagen coating reduced these inconsistences), which is in agreement to previous publications, where soft substrates promoted cell-cell contact, while stiff substrates induced cell spreading and migration [66,67]. Quantitative cytoskeleton orientation analysis showed that cells on all substrates aligned in the direction of the grooves, as it has been observed previously for various cell types [16,64,68,69], considering that cells, via contact guidance, preferentially follow the groove edge as opposed to crossing the gap [60,[69], [70], [71], [72]]. It is also important to note that cells aligned less strictly to the direction of the grooves when they were cultured on the 50 kPa substrates, which can be attributed to their lowest groove depth. To substantiate this, one should consider that a depth-dependant cell alignment and elongation has been observed previously [16,73] and was attributed to the formation of enhanced actin filaments and focal adhesions [74]. The influence of rigidity also cannot be excluded, as previous studies have shown higher elongation and lower orientation of cardiomyocytes on polystyrene (2 GPa) than polyurethane (4 MPa) substrates of the same topography (the difference in chemistry may also have contributed to the observed difference in cellular morphometry) [73]. However, considering that cells preferentially migrate from soft to rigid substrates (durotaxis) [75], we believe that both these variables (groove depth and substrate rigidity) play an important role in cellular morphology and motility. Of interest is also to note that cells lost their fidelity to the direction of the grooves as a function of time in culture. This could be possibly attributed to the progressively increased ECM deposition that covered the grooves, as also was observed with the MMC groups. We have previously shown that protein adsorption on implanted imprinted substrates does not induce anisotropic neotissue formation [16,68], which fits well with this theory.

Quantification of nuclei orientation, area and aspect ratio also made evident nuclei parallel alignment to the grooves, although to a less extent on the softest substrates, which can be again attributed to the influence of groove depth/substrate rigidity effect. Nuclei alignment was observed with two major peaks found at 70° and 110° compared to the singular 90° peak observed with cellular alignment. This indicates that nuclei were not as aligned as the cytoskeleton, which may be attributed to the reduced, in comparison to cytoskeleton, nuclei pliability to physical cues [69]. Nuclei area was smaller and aspect ratio was higher on cells cultured on PDMS than on TCP, which can be attributed to their considerable difference in stiffness, as cells have been shown to spread more on stiffer matrices [[75], [76], [77]]. Cells cultured on grooved substrates exhibited smaller nuclear area and higher aspect ratio, while collagen type I coating resulted in larger area and smaller aspect ratio, which is in agreement with previous publications, where enhanced cytoskeletal spreading on collagen type I coated substrates was observed [78]. MMC treatment had a similar effect on the collagen type I coating, resulting in increased nuclei area as a function of time on culture, which was not surprising, considering that MMC enhances ECM deposition [29]. Following FAK analysis, FAK levels were found to be higher on TCP than PDMS substrates and when substrates were coated with collagen type I, while they were lower in the presence of MMC. Focal adhesion size has been shown to correlate with cell spreading [79], which in turn is related to nuclear area [80]; thus, this increase in FAK and nuclear area in cells on TCP could be due to increased cell area and the establishment of larger focal adhesions. Also, FAK is involved in the mechano-sensing of collagen-coated surfaces, while different kinases are involved in the mechano-sensing of substrates coated with different proteins, such as fibronectin [81,82]. As fibronectin provides a template for collagen type I and collagen type III assembly [83,84] and MMC leads to significant increases in the amount of surface adherent fibronectin [85], this may be the reason for reduced FAK in MMC groups. An alternative theory could be based on detachment, as FAK deactivation is associated with detachment-induced apoptosis [86]. Considering that MMC significantly increases ECM deposition, the forces between media and cell-ECM layer are a lot stronger than the forces between substrate and cell-ECM layer, resulting in cell-ECM layer detachment. Finally, rigidity cannot be excluded and may be the driving force in this occasion, as increased rigidity has been shown to lead to increased focal adhesion number and total FAK activation [79,87,88]. In our case, PDMS had lower FAK levels than TCP, collagen-coated substrates had lower FAK levels than non-collagen- coated substrates and MMC groups had lower FAK levels than non-MMC groups; all these observations validate the notion of rigidity-dependent FAK expression.

### 4.3 Basic cellular function analysis

Cell number, metabolic activity and viability analyses confirmed that all substrates supported cell growth. Cell number and metabolic activity were significantly higher when cells were grown on TCP than PDMS substrates, which is in accordance with previous publications indicating higher cell number on stiff, as opposed to on soft, substrates [89]. Given that the metabolic activity of cells on PDMS substrates was normalised to the TCP, the lower values are attributed to the lower cell number. The investigation of a range of cell types on diverse surface geometries and chemistries has yielded both positive [[90], [91], [92]] and neutral [[93], [94], [95]] results regarding cell number, suggesting that there may be various factors at play in whether topography affects cell number. Collagen type I coating increased both cell number and metabolic activity, which has been well documented previously [96,97]. Some differences were also observed in cell number and metabolic activity when MMC was used. Considering that carrageenan, the MMC used herein, (1) has been used previously in human tenocyte [29], human bone marrow stem cell [30], human dermal fibroblast [98], human chondrocyte [99] and human corneal fibroblast [33] cultures without any negative effect and (ii) the Live/Dead® assay did not show a substantial number of dead cells, we attribute the reduction in cell number and metabolic activity, when observed, to the low rigidity of the PDMS substrates, as cells do not attach as tightly on soft substrates [67,100].

### 4.4 Protein analysis

Overall, all the tested ECM proteins were detected on all the PDMS substrates as early as day 3, suggesting physiological tenocyte function. Specifically, starting with the collagen family [101], collagen type I is the predominant ECM protein found in soft connective tissues and plays key role in their structure and function [102,103]. Collagen type III is the second most abundant ECM protein, is often associated with collagen type I fibrillogenesis, is of lower mechanical resilience than collagen type I and high collagen type III to collagen type I ratio is associated with impaired healing process, ageing and pathogenesis [[104], [105], [106], [107], [108], [109], [110], [111]]. Collagen type IV, a basement membrane collagen [112], is a normal constituent for tendon [103,113] and skin [114,115], and plays an important role in wound healing [116]. Collagen type V serves a crucial regulatory role in fibrillogenesis [117,118]. Collagen type VI is a non-fibrillar collagen that has been found in most tissues [119], is ubiquitously distributed in tendon [120], provides structural and mechanical integrity [121,122] and also acts as a key regulator of matrix signals [[123], [124], [125]]. Fibronectin is an ECM glycoprotein involved in both cell adhesion and migration [126], is present on the surface of collagen, acts as a template for the oriented deposition of collagen and is upregulated to facilitate wound healing [[127], [128], [129]].

The most striking result from this set of experiments is that at all time points and in particular at day 14, MMC, following the principles of excluded volume effect [[130], [131], [132]], substantially increased collagen types I, III, IV, V and VI deposition, as has been shown before with various cell types [29,30,33,133,134], including tenocytes [21,22,135]. It is worth noting that at early time points, collagen type I was detected even at the groups without MMC, as the antibody used was not specific to human collagen type I and was also able to detect the bovine collagen type I that had been used to coat the substrates. At day 14 though, the detected collagen was due to MMC, as almost no collagen type I was detected in the without MMC groups. We believe that by day 14, the coated collagen type I had been processed by the cells and/or degraded by the matrix metalloproteinases of the serum and therefore had minimum effect. With respect to the influence of substrate rigidity and surface topography to ECM synthesis and deposition, although some differences were observed, they were not consistent across all molecules, rigidities and absence/presence of surface topography, collagen type I coating and MMC. We feel that this is normal, as we were not able to identify literature suggesting otherwise (only one paper has shown increased, albeit marginal, sulphated glycosaminoglycans synthesis in human corneal stromal cells that were grown on anisotropic, as opposed to planar, substrates [136]).

Regarding the morphology of ECM deposited proteins, in the absence of MMC they had a more aligned structure, while they were more globular when cells were cultured with MMC. As demonstrated herein, FAK was significantly reduced under MMC conditions, which could account for the lack of alignment in MMC cultures. Another possibility is that the cells were unable to efficiently organise the vast amounts of deposited ECM into aligned fibrils. Potentially, crowders that enhance ECM deposition, albeit in lower rates, such as Ficoll™[31], may be more suitable for ECM alignment in such low dimensionality grooves. To substantiate this one should consider that Ficoll™ has already been shown to induce aligned ECM deposition [137], possibly due to its elongated conformation in physiological media [130]. Alternatively, a larger groove dimensionality (e.g. 10 μm wide and 3 μm deep [138], 9 μm wide and 1–2 μm deep [139]) should be used that has been shown to induce cellular and deposited ECM alignment in the direction of the orientation of the grooves. Anisotropic electrospinning can also be used, as the cells can penetrate within the three-dimensional architecture, which can induce anisotropic alignment of both seeded cells and deposited ECM [140,141], even under the same MMC conditions used herein [142]. Of course, mechanical stimulation can also be used, which has been shown to induce bidirectional cell and deposited ECM orientation, even in the presence of MMC [22].

### 4.5 Gene analysis

Gene expression analysis was performed at days 7 and 14 (as sufficient quantities of mRNA at day 3 could not be obtained) for tenogenic, chondrogenic and osteogenic markers to assess whether the cues investigated aided in the phenotype maintenance of human tenocytes. It is also worth noting that COL2A1 (the major ECM component of cartilage [143] and indicative marker of tenocyte *trans*-differentiation to fibro-chondrocyte [14,15]) and TNMD (a mechanosensitive, type II transmembrane glycoprotein that is highly expressed in developing and mature tendons and ligaments [144,145]) were below the detectable limit of the assay. In our opinion, this observation is not related to phenotype, as previous studies have also failed to detect TNMD [146] and were successful in detecting COL2A1 [147] in tenocyte cultures. To further substantiate our claim, one should consider that SCXA, a transcriptional activator of TNMD in tenocytes [148], was detectable; TNMD is considered a late marker of tendon formation [149] and COL2A1 expression is in general increased at late (>28 days) stages of chondrogenic differentiation [[150], [151], [152], [153], [154]]. At day 14, the longest time point assessed, cells cultured without MMC (without/with collagen type I coating) on the softest (50 kPa) and grooved substrates maintained most closely their native phenotype. For the other groups, upregulation of ACAN (a large proteoglycan that plays a key role in articular cartilage ECM assembly, endows it with the ability to withstand loads and is involved in chondrocyte phenotype maintenance and chondrogenic differentiation of stem cells [[155], [156], [157]]) expression was observed. COMP (a member of the thrombospondin gene family that is involved in human limb development, chondrocyte phenotype maintenance and chondrogenic induction of stem cells and is upregulated in osteoarthritis [[158], [159], [160], [161]]) expression was also upregulated. On the other hand, TNC (a matricellular protein that is involved in cellular adaptation to mechanical loads, provides elasticity to tissues subjected to heavy mechanical loads, helps in the establishment and maintenance of fibrocartilage regions of tendons, decreases adhesions and is frequently used as marker for tenogenic phenotype maintenance and induction across different species, especially when mechanical stimulation is utilised [[162], [163], [164], [165]]) expression was downregulated. Collectively, the observed upregulation of ACAN and COMP gene expression and downregulation of TNC gene expression indicate loss of native phenotype. This is in accordance with previous publications suggesting that for tenogenic phenotype maintenance in long-term cultures, grooved and soft substrates are required [16]. At day 14 in the presence of MMC, the 130 kPa grooved substrate without collagen coating was the most promising, as it upregulated SCXA and THBS4 and only downregulated COL1A1. SCXA is a transcription factor critical for the normal development of tendon tissues and tenogenic differentiation of stem cells [166,167] and THBS4 is a glycoprotein that plays a key role in normal organisation of tendon ECM [168,169]. The collagen downregulation can be explained via the cell negative feedback loop phenomena during which accumulation of a protein induces cells to suppress the expression of the corresponding gene [[170], [171], [172]] and has been also observed previously under MMC and oxygen tension conditions in human tenocyte cultures [135].

## 5 Summary

Considering that cells in their native tissue context are subjected simultaneously to many and different stimuli that together determine their function, we hypothesised that to effectively control cell fate *in vitro*, it is also essential to recruit multiple and tissue-specific *in vitro* microenvironment modulators. To validate our hypothesis, we selected human tenocytes, a cell population notorious for readily losing its phenotype during *ex vivo* culture. Among the various bioinspired *in vitro* cell fate regulators, we selected material- (e.g. surface topography, substrate stiffness) and ECM- (e.g. collagen type I coating, MMC) based approaches, due to their relative simplicity, potency and scalability potential. As cellular phenotype readouts, we selected an array of basic function, morphometric, protein synthesis and gene expression assays. Overall, cell viability was not affected by any of the variables assessed. In the absence of MMC, the 50, 130 and 1,000 kPa grooved substrates (with/without collagen type I coating) clearly induced physiological cell and ECM alignment in the direction of the grooves. However, at day 14, the 130 and the 1,000 kPa grooved substrates (with/without collagen type I coating) upregulated ACAN and/or COMP expression, indicative of chondrogenic *trans*-differentiation, while the 50 kPa grooved substrate (with/without collagen type I coating) did not alter gene expression. Considering that gene analysis was conducted using as control the TCP of the same time point, our data suggest that the extra work (i.e. development of softer TCP and grooved substrates) is not really necessary. On the other hand, MMC clearly significantly enhanced and accelerated physiological ECM deposition. However, the deposited ECM was not aligned in the direction of the grooves, as either the appropriate groove dimensionality was not used or carrageenan, the crowder used, deposited ECM at faster rates than cells could have processed in an organised fashion. Nonetheless, under MMC conditions at day 14, the 130 kPa grooved substrate without collagen type I coating upregulated SCXA and THBS4 and only downregulated COL1A1, possibly due to negative feedback loop effect that was initiated by the enhanced deposited ECM.

Considering that MMC coupled with low rigidity and grooved substrates maintained basic cellular functions; substantially increased and accelerated physiological ECM deposition (albeit, not in an anisotropic orientation, due to the incorrect selection of surface topography dimensionality); and upregulated/maintained the most tenogenic genes and downregulated/maintained the most *trans*-differentiation genes, we feel that appropriately selected combination approaches hold promise for the accelerated development of functional and clinically relevant tissue engineering medicines.

## 6 Limitations

Herein, we ventured to assess the influence of various *in vitro* microenvironment modulators (e.g. surface topography, substrate stiffness, collagen coating, MMC) on human tenocyte response. Despite the substantial work conducted that, in our opinion, has advanced knowledge in the field of tendon tissue engineering, we feel obliged to also point out certain limitations that may inform future studies. First, all data were obtained from cells from a single donor/tissue and without taking into account the tenocyte subtypes [173]; as such, further validation should be conducted using cells from multiple donors, considering gender, age, tissue and tenocyte subtype variations. Second, as we did not have access to the tissue from which the cells were extracted from, all data were correlated to cells on TCP; thus, subsequent studies should correlate obtained data to the tissue from which the cells were obtained from. Third, all cultures herein were conducted in the presence of FBS, which is known to induce phenotype losses; thus, future studies should be directed towards development of tendon-specific serum-/animal component-free media formulations (or at least, allogenic serum media formulations). Fourth, herein a single (architecture and dimensionality) topography, MMC agent and ECM molecule coating and only three substrate rigidities were assessed; therefore, using the power of experimental design and lab-on-a-chip technologies, future studies should investigate multiple variances of the aforementioned conditions.

## 7 Conclusions

As cells *in vivo* are subjected simultaneously to manifold signals, combination approaches are continuously gaining pace to also control cell fate *in vitro*. Herein, we assessed the combined potential of scalable *in vitro* microenvironment modulators (surface topography, substrate rigidity, collagen type I coating and MMC) in controlling cell fate. Our data suggest that MMC coupled with low rigidity substrates of anisotropic surface topography positively regulated human tenocyte phenotype. This work further corroborates the notion for combined stimuli tissue engineering and regenerative medicine.

## Author contribution

D.I.Z. had the overall responsibility of the work. D.I.Z. and C.N.M.R. designed the study. D.I.Z. and E.P. revised the figures. C.N.M.R. performed the PDMS substrate fabrication; atomic force, brightfield and fluorescence microscopy analyses; optical profilometry analysis; cell viability and metabolic activity assays; immunocytochemistry and ImageJ analyses. C.N.M.R. and N.S. performed the cell culture experiments. C.N.M.R., P.R. and D.G. performed gene expression experiments and measurements. C.N.M.R. and P.R. performed focal adhesion kinase measurements and analysis. C.N.M.R. processed all of the experimental data, performed the statistical analysis and designed the figures. M.J.B. was involved in the fabrication of substrates with different rigidity. A.O’R. performed the silicon wafer fabrication. C.N.M.R. wrote the first draft of the manuscript and D.I.Z. edited and finalised manuscript and figures. All authors discussed and approved the final version of the manuscript.

## Data availability

The raw data required to reproduce these findings are available on request from the corresponding author. The processed data required to reproduce these findings are available on request from the corresponding author.

## Declaration of competing interest

The authors declare that they have no known competing financial interests or personal relationships that could have appeared to influence the work reported in this paper.
