# Supplementary material for: A combined physicochemical approach towards human tenocyte phenotype maintenance
Source: Mater Today Bio. 2021 Sep 10;12:100130. doi: 10.1016/j.mtbio.2021.100130 (PMC8488312; doi:10.1016/j.mtbio.2021.100130)

Supporting Information

Title

A combined physicochemical approach towards human tenocyte phenotype maintenance

Authors

Christina N. M. Ryan (1, 2), Eugenia Pugliese (1, 2), Naledi Shologu (1, 2), Diana Gaspar (1, 2), Peadar Rooney (2), Md Nahidul Islam (2, 3, 4), Alan O'Riordan (5), Manus J. Biggs (2), Matthew D. Griffin (2, 3), Dimitrios I. Zeugolis* (1, 2, 6)

Affiliations

(1) Regenerative, Modular & Developmental Engineering Laboratory (REMODEL), Biomedical Sciences Building, National University of Ireland Galway (NUI Galway), Galway, Ireland

(2) Science Foundation Ireland (SFI) Centre for Research in Medical Devices (CÚRAM), Biomedical Sciences Building, National University of Ireland Galway (NUI Galway), Galway, Ireland

(3) Regenerative Medicine Institute (REMEDI), School of Medicine, Biomedical Sciences Building, National University of Ireland Galway (NUI Galway), Galway, Ireland

(4) Discipline of Biochemistry, School of Natural Sciences, National University of Ireland Galway (NUI Galway), Galway, Ireland

(5) Tyndall National Institute, University College Cork (UCC), Cork, Ireland

(6) Regenerative, Modular & Developmental Engineering Laboratory (REMODEL), Charles Institute of Dermatology, Conway Institute of Biomolecular & Biomedical Research and School of Mechanical & Materials Engineering, University College Dublin (UCD), Dublin, Ireland

* Corresponding Author: Dimitrios I. Zeugolis, REMODEL, NUI Galway & UCD. Telephone: +353 17 16 18 84; Email: dimitrios.zevgolis@ucd.ie

Supplementary Table S1: Primary and secondary antibodies used for immunocytochemistry analysis.

Protein Target	Primary Antibody	Company	Dilution	Secondary Antibody	Company	Dilution
Collagen I	Mouse (ab90395)	Abcam (UK)	1 to 200	Goat anti-mouse AlexaFluor® 555 (A-21422)	ThermoFisher Scientific (Ireland)	1 to 500
Collagen III	Rabbit (ab7778)	Abcam (UK)	1 to 200	Goat anti-rabbit AlexaFluor® 488 (A11034)	Life Technologies (UK)	1 to 500
Collagen IV	Rabbit (ab6586)	Abcam (UK)	1 to 200	Goat anti-rabbit AlexaFluor® 488 (A11034)	Life Technologies (UK)	1 to 500
Collagen V	Rabbit (ab7046)	Abcam (UK)	1 to 200	Donkey anti-rabbit AlexaFluor® 555 (ab150074)	Abcam (UK)	1 to 500

Collagen VI	Rabbit (ab6588)	Abcam (UK)	1 to 200	Goat anti-rabbit AlexaFluor® 488 (A11034)	Life Technologies (UK)	1 to 500
Fibronectin	Rabbit (ab2413)	Abcam (UK)	1 to 200	Donkey anti-rabbit AlexaFluor® 555 (ab150074)	Abcam (UK)	1 to 500

Supplementary Table S2: Primers used in RT-qPCR TaqMan® RealTime ready Custom Panel.

	Gene Name	Gene Symbol	Forward Primer (5' to 3')	Reverse Primer (5' to 3')
Tenogenic	Collagen I	COL1A1	AGGTGAAGCAGGCAAACCT	CTCGCCAGGGAAACCTCT
	Scleraxis homolog A	SCXA	CCCAAACAGATCTGCACCTT	TCTTTCTGTCGCGGTCCTT
	Tenascin C	TNC	CCTTGCTGTAGAGGTCGTCA	CCAACCTCAGACACGGCTA
	Tenomodulin	TNMD	TGTATTGGATCAATCCCACTCTAA	TCGTTGGCAGGAAAGTGAA
	Thrombospondin 4	THBS4	CTACCGCTGGTTCCTACAGC	GAGCCTTCATAAAATCGTACCC
Osteogenic	Runt-related transcription factor	RUNX2	GCCTAGGCGCATTTCAGAT	CTGAGAGTGGAAGGCCAGAG
	Osteonectin	SPARC	TTGATGATGGTGCAGAGGAA	CTTGCCGTGTTTGCAGTG
	Bone sialoprotein	IBSP	CAGGGCAGTAGTGACTCATCC	TCGATTCTTCATTGTTTTCTCCT
Chondrogenic	Collagen II	COL2A1	CTGGTCCTCAAGGCAAAGTT	GAGGTCCAGGACGACCATC
	Collagen X	COL10A1	CAGTTCTTCATTCCCTACACCA	AGGACTTCCGTAGCCTGGTT
	Aggrecan	ACAN	GAACGACAGGACCATCGAA	AAAGTTGTCAGGCTGGTTGG
	Cartilage oligomeric protein	COMP	GGAGATCGTGCAGACAATGA	GTCATCCGTGACCGTGTTC

Housekeeping	Glyceraldehyde-3-phosphate dehydrogenase	GAPDH	AGCCACATCGCTCAGACAC	GCCCAATACGACCAAATCC

Supplementary Table S3: DMA data of PDMS substrates with different Sylgard® ratios. * From this point onwards, experimental groups were referred to by stiffness as 1,000 kPa (976.8 ± 252.7 kPa), 130 kPa (128.7 ± 1.8 kPa), 50 kPa (52.7 ± 5.6 kPa) and 4 kPa (4.0 ± 0.2).

**Sylgard® 184 to Sylgard® 527 Ratio**	**Storage modulus (kPa)**	**Loss modulus (kPa)**	**Phase angle**
1 to 0	976.8 ± 252.7*	184.1 ± 40.6	10.9 ± 2.1
1 to 1	128.7 ± 1.8*	15.7 ± 6.1	7.0 ± 2.7
1 to 5	52.7 ± 5.6*	6.8 ± 1.2	7.5 ± 1.3
0 to 1	4.0 ± 0.2*	1.5 ± 0.2	21.0 ± 2.6

Supplementary Table S4: AFM analysis of PDMS substrates as a function of rigidity, collagen type I coating and anisotropic surface topography. Surface roughness was not affected by any of the tested variables. Groove depth was similar ($p > 0.05$) among the non-collagen type I coated substrates and when the substrates were coated with collagen type I, the 50 kPa substrate exhibited the lowest ($p < 0.05$) groove depth. In the absence of collagen coating, the 1,000 kPa substrate exhibited the highest ($p < 0.05$) groove width and in the presence of collagen coating, the 50 kPa substrate exhibited the highest ($p < 0.05$) groove width. In the absence of collagen coating, the 130 kPa substrate exhibited the highest ($p < 0.05$) line width and in the presence of collagen coating, no statistically significant differences were observed between the groups.

**PDMS substrate stiffness**	**Collagen type I coating (mg/ml)**	**Surface roughness (nm)**	**Groove depth (nm)**	**Groove width (nm)**	**Line width (nm)**
1,000 kPa	0	19 ± 23	1,987 ± 88	2,114 ± 435	1,439 ± 130
	0.5	17 ± 21	2,022 ± 102	2,180 ± 115	1,347 ± 156
130 kPa	0	9 ± 7	1,938 ± 283	1,746 ± 152	1,917 ± 231
	0.5	29 ± 21	1,947 ± 87	2,319 ± 138	1,367 ± 77
50 kPa	0	12 ± 7	1,855 ± 186	1,697 ± 112	1,787 ± 202
	0.5	12 ± 6	1,468 ± 219	2,835 ± 1,113	1,276 ± 138

Supplementary Figure S1: Cytoskeleton orientation at day 3 on tissue culture plastic (TCP) without and with collagen type I coating (- Col, + Col) and macromolecular crowding (- MMC, + MMC) and on substrates of varying stiffness (1,000 kPa, 130 kPa, 50 kPa), surface topography [planar (P), grooved (G)], collagen type I coating (- Col, + Col) and macromolecular crowding (- MMC, + MMC). The cells' cytoskeleton aligned parallel to the orientation of the groves, independently of the collagen type I coating, MMM and substrate rigidity.

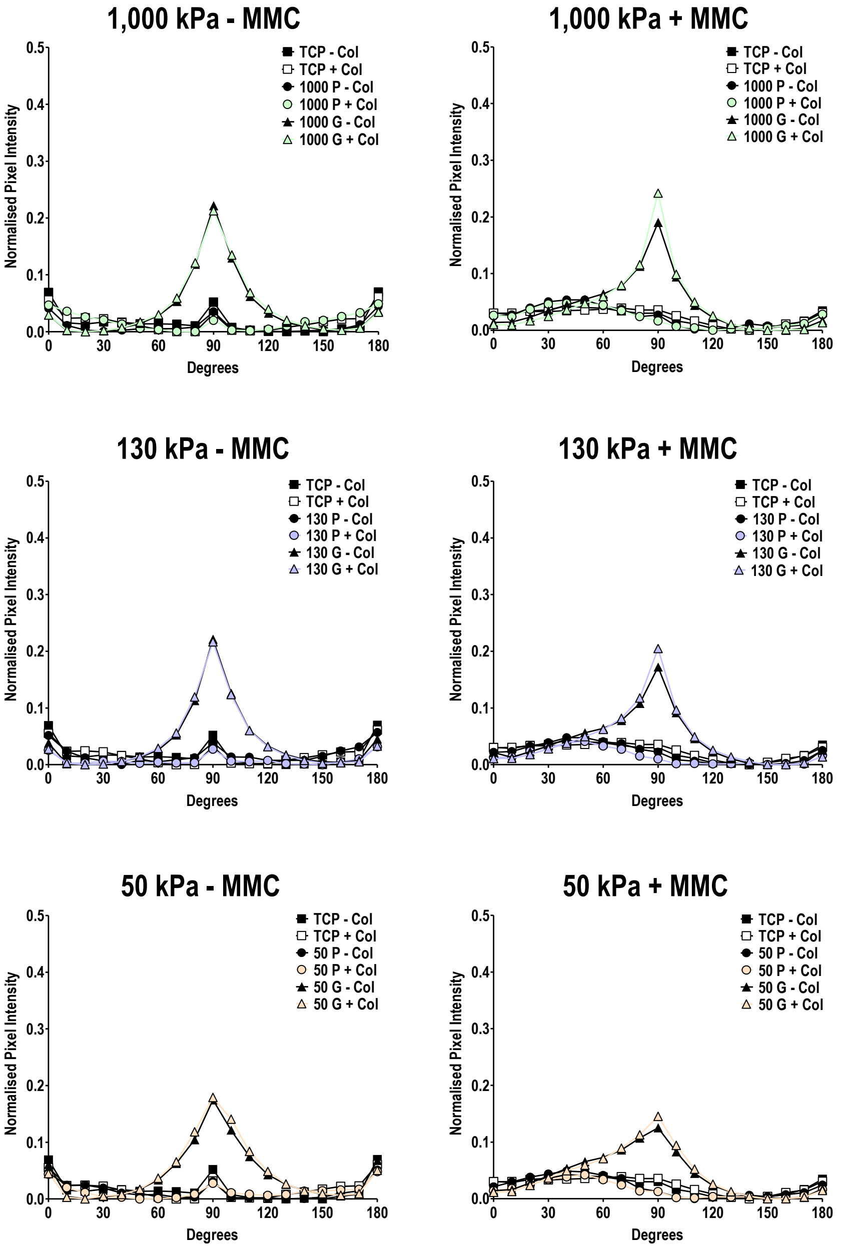

Supplementary Figure S2: Cytoskeleton orientation at day 7 on tissue culture plastic (TCP) without and with collagen type I coating (- Col, + Col) and macromolecular crowding (- MMC, + MMC) and on substrates of varying stiffness (1,000 kPa, 130 kPa, 50 kPa), surface topography [planar (P), grooved (G)], collagen type I coating (- Col, + Col) and macromolecular crowding (- MMC, + MMC). The cells' cytoskeleton aligned parallel to the orientation of the groves, independently of the collagen type I coating, MMM and substrate rigidity.

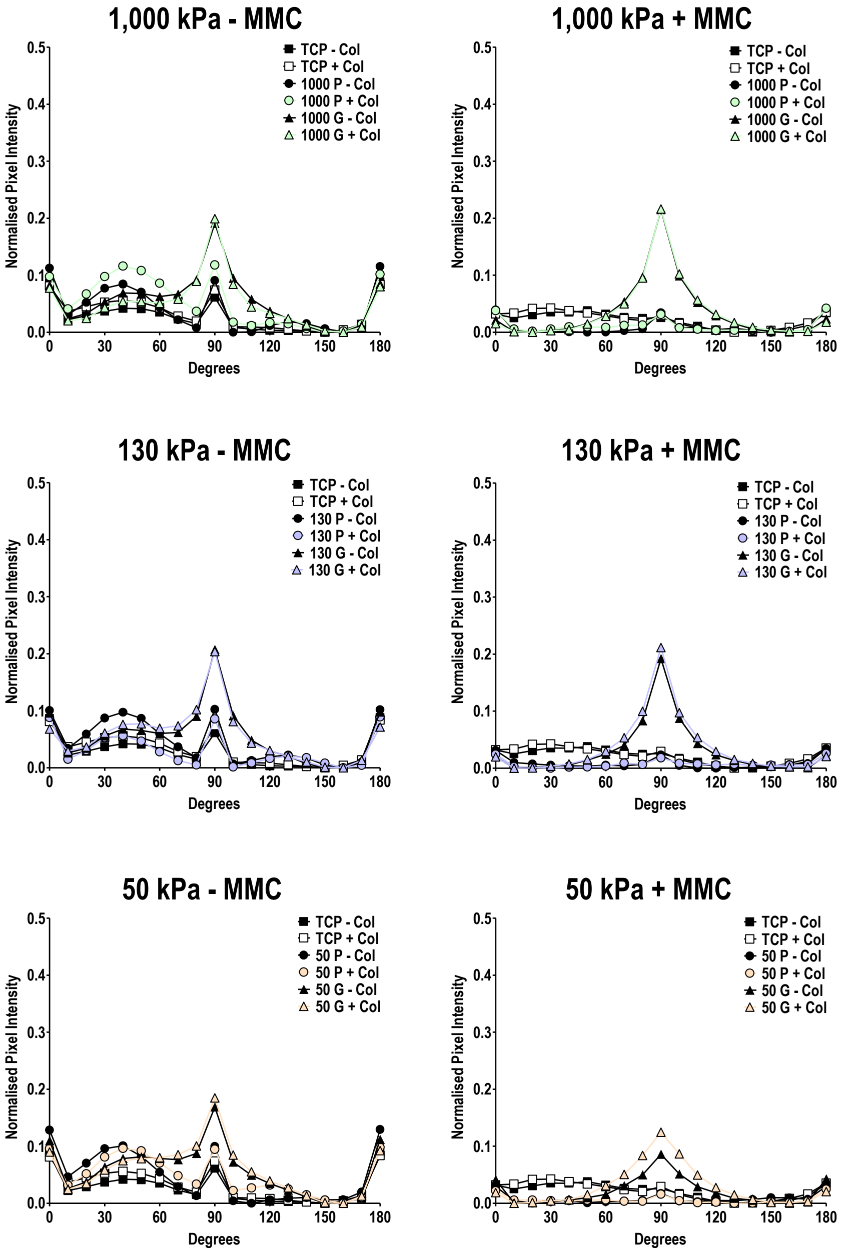

Supplementary Figure S3: Cytoskeleton orientation at day 14 on tissue culture plastic (TCP) without and with collagen type I coating (- Col, + Col) and macromolecular crowding (- MMC, + MMC) and on substrates of varying stiffness (1,000 kPa, 130 kPa, 50 kPa), surface topography [planar (P), grooved (G)], collagen type I coating (- Col, + Col) and macromolecular crowding (- MMC, + MMC). The cells' cytoskeleton aligned parallel to the orientation of the groves, independently of the collagen type I coating, MMM and substrate rigidity.

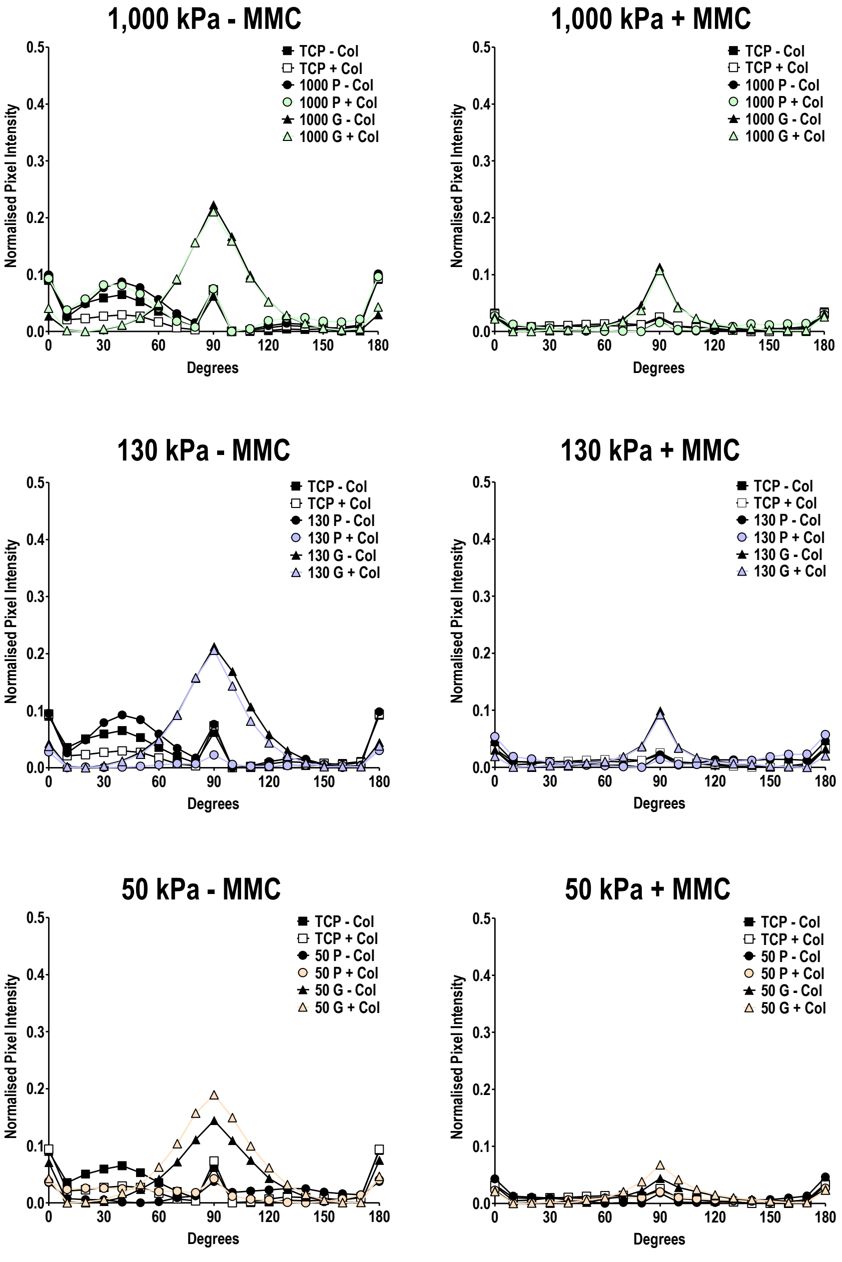

Supplementary Figure S4: Nuclear orientation at day 3 on tissue culture plastic (TCP) without and with collagen type I coating (- Col, + Col) and macromolecular crowding (- MMC, + MMC) and on substrates of varying stiffness (1,000 kPa, 130 kPa, 50 kPa), surface topography [planar (P), grooved (G)], collagen type I coating (- Col, + Col) and macromolecular crowding (- MMC, + MMC). The cells' nuclei aligned parallel to the orientation of the grooved surface topography of the PDMS substrates, independently of the collagen type I coating, MMM and substrate rigidity.

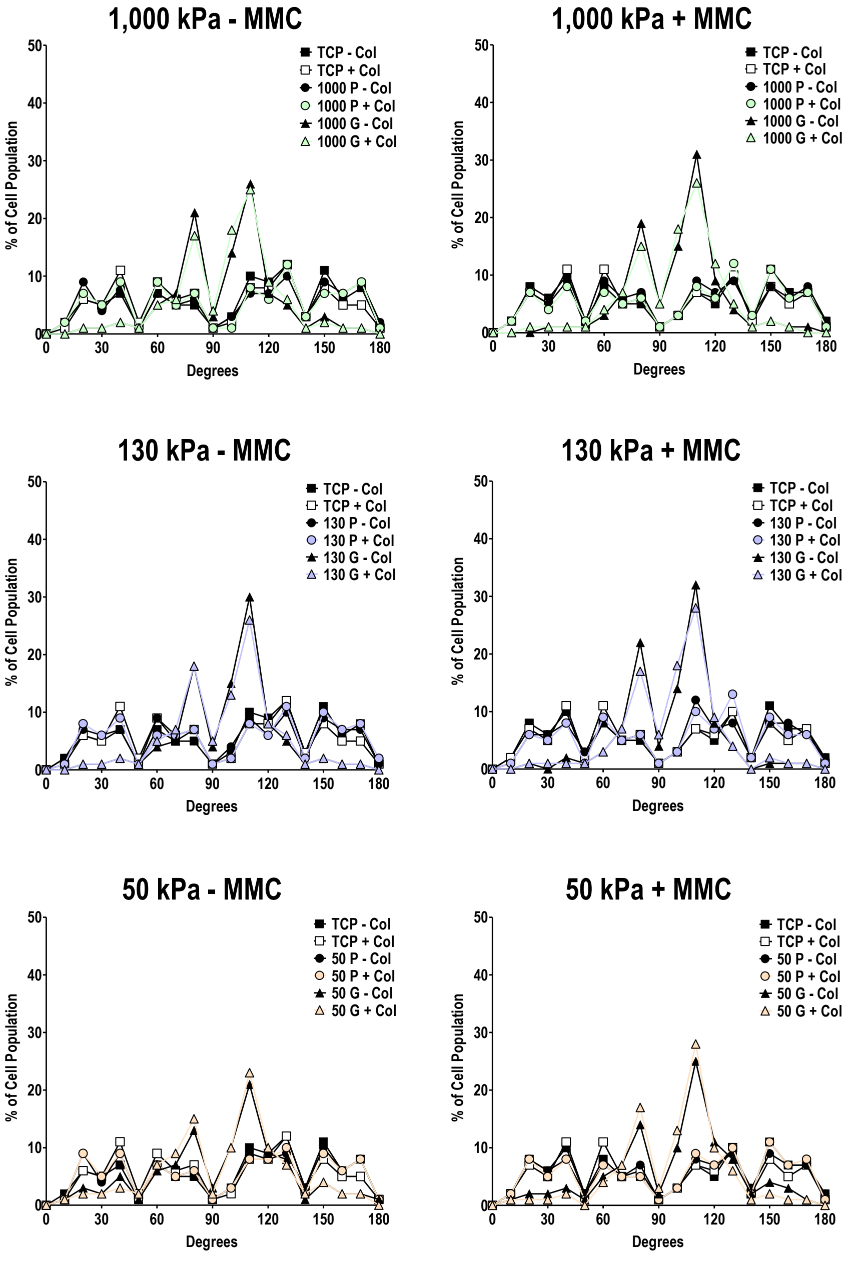

Supplementary Figure S5: Nuclear orientation at day 7 on tissue culture plastic (TCP) without and with collagen type I coating (- Col, + Col) and macromolecular crowding (- MMC, + MMC) and on substrates of varying stiffness (1,000 kPa, 130 kPa, 50 kPa), surface topography [planar (P), grooved (G)], collagen type I coating (- Col, + Col) and macromolecular crowding (- MMC, + MMC). The cells' nuclei aligned parallel to the orientation of the grooved surface topography of the PDMS substrates, independently of the collagen type I coating, MMM and substrate rigidity.

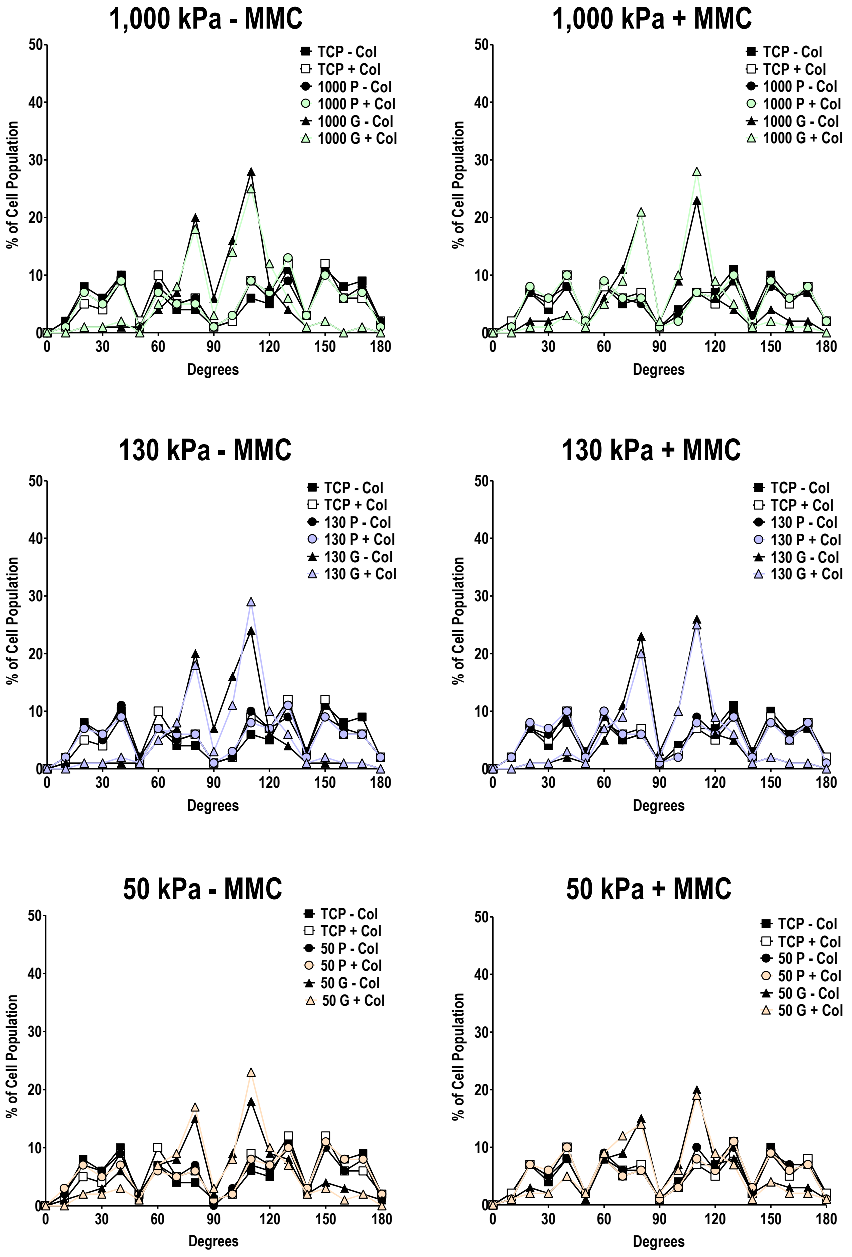

Supplementary Figure S6: Nuclear orientation at day 14 on tissue culture plastic (TCP) without and with collagen type I coating (- Col, + Col) and macromolecular crowding (- MMC, + MMC) and on substrates of varying stiffness (1,000 kPa, 130 kPa, 50 kPa), surface topography [planar (P), grooved (G)], collagen type I coating (- Col, + Col) and macromolecular crowding (- MMC, + MMC). The cells' nuclei aligned parallel to the orientation of the grooved surface topography of the PDMS substrates, independently of the collagen type I coating, MMM and substrate rigidity.

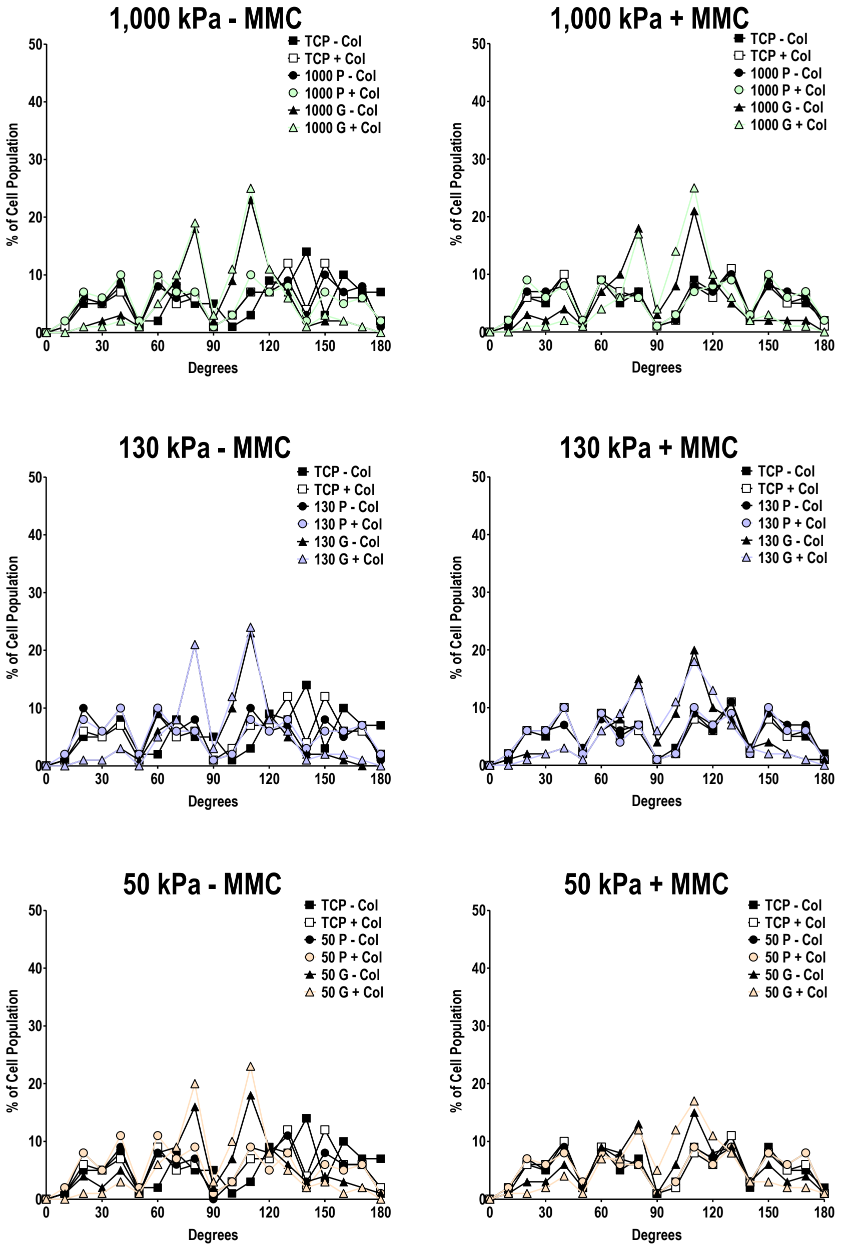

Supplementary Figure S7: Nuclear area of cells on tissue culture plastic (TCP) without and with collagen type I coating (- Col, + Col) and macromolecular crowding (- MMC, + MMC) and on substrates of varying stiffness (1,000 kPa, 130 kPa, 50 kPa), surface topography [planar (P), grooved (G)], collagen type I coating (- Col, + Col) and macromolecular crowding (- MMC, + MMC) at day 3, day 7 and day 14. * indicates statistically significant difference ($p < 0.05$) between without and with collagen type I coating and between without and with MMC, # indicates statistical difference ($p < 0.05$) between TCP and PDMS substrates and + indicates statistical difference ($p < 0.05$) between planar and grooved topography.

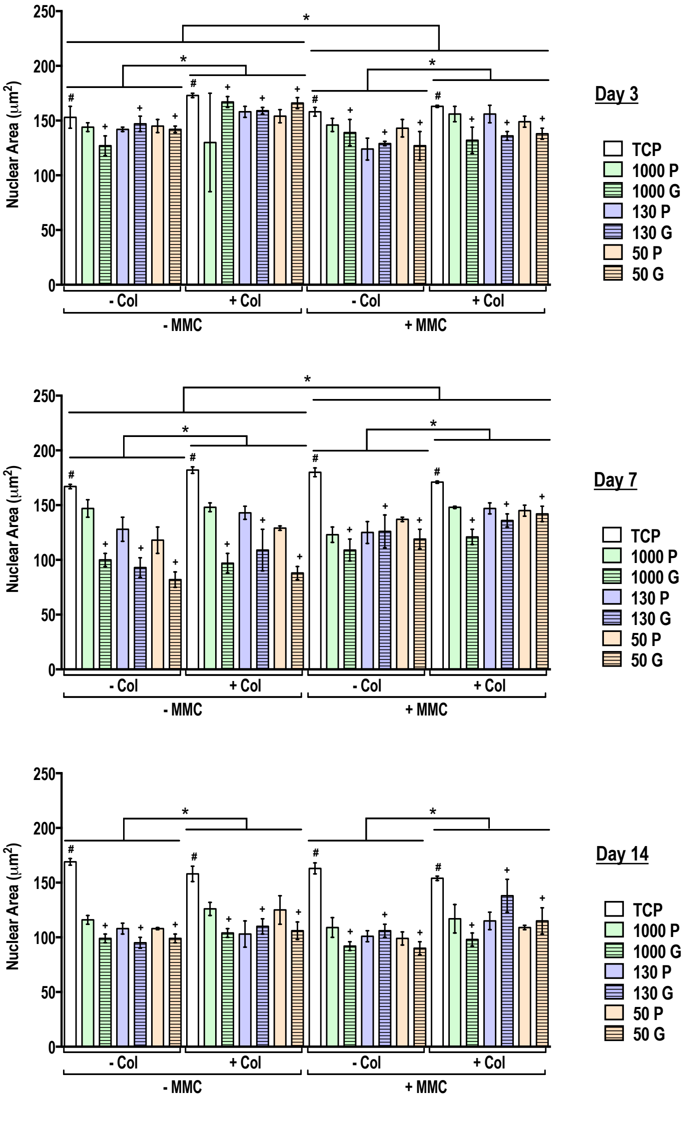

Supplementary Figure S8: Nuclear elongation of cells on tissue culture plastic (TCP) without and with collagen type I coating (- Col, + Col) and macromolecular crowding (- MMC, + MMC) and on substrates of varying stiffness (1,000 kPa, 130 kPa, 50 kPa), surface topography [planar (P), grooved (G)], collagen type I coating (- Col, + Col) and macromolecular crowding (- MMC, + MMC) at day 3, day 7 and day 14. * indicates statistically significant difference ($p < 0.05$) between without and with collagen type I coating and between without and with MMC, # indicates statistical difference ($p < 0.05$) between TCP and PDMS substrates and + indicates statistical difference ($p < 0.05$) between planar and grooved topography.

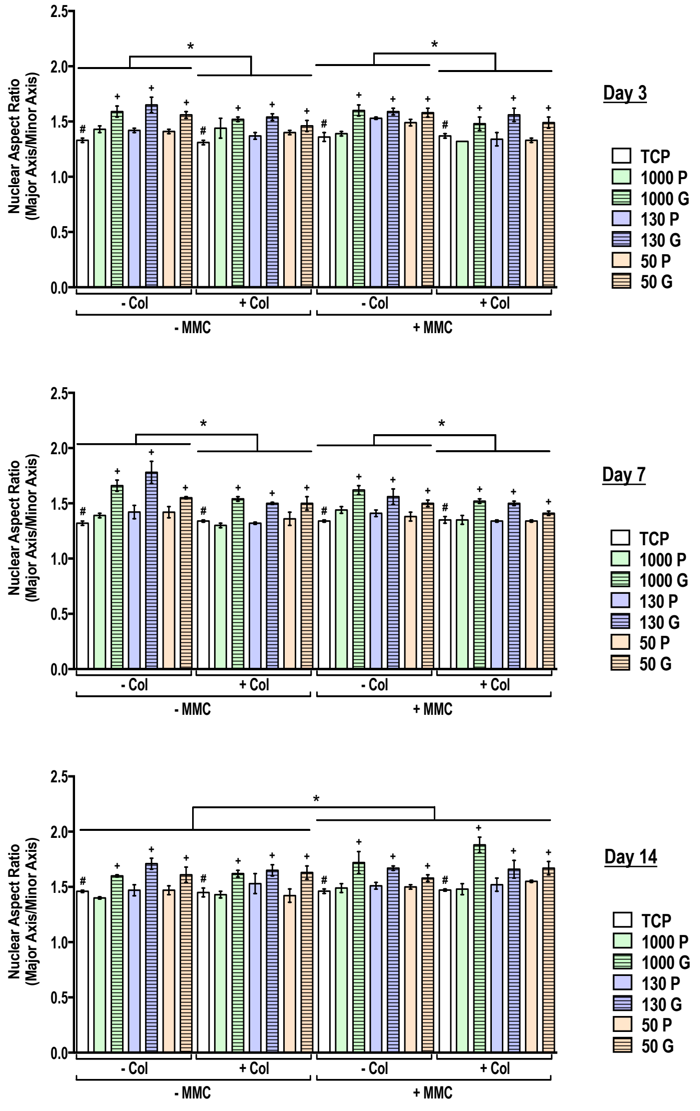

Supplementary Figure S9: % FAK of total protein from human tenocytes cultured on tissue culture plastic (TCP) without and with collagen type I coating (- Col, + Col) and macromolecular crowding (- MMC, + MMC) and on substrates of varying stiffness (1,000 kPa, 130 kPa, 50 kPa), surface topography [planar (P), grooved (G)], collagen type I coating (- Col, + Col) and macromolecular crowding (- MMC, + MMC) at day 3. ND indicates values obtained below the detection limits of ELISA. * indicates statistically significant difference ($p < 0.05$) between without and with collagen type I coating and between without and with MMC, # indicates statistical difference ($p < 0.05$) between TCP and PDMS substrates and + indicates statistical difference ($p < 0.05$) between planar and grooved topography.

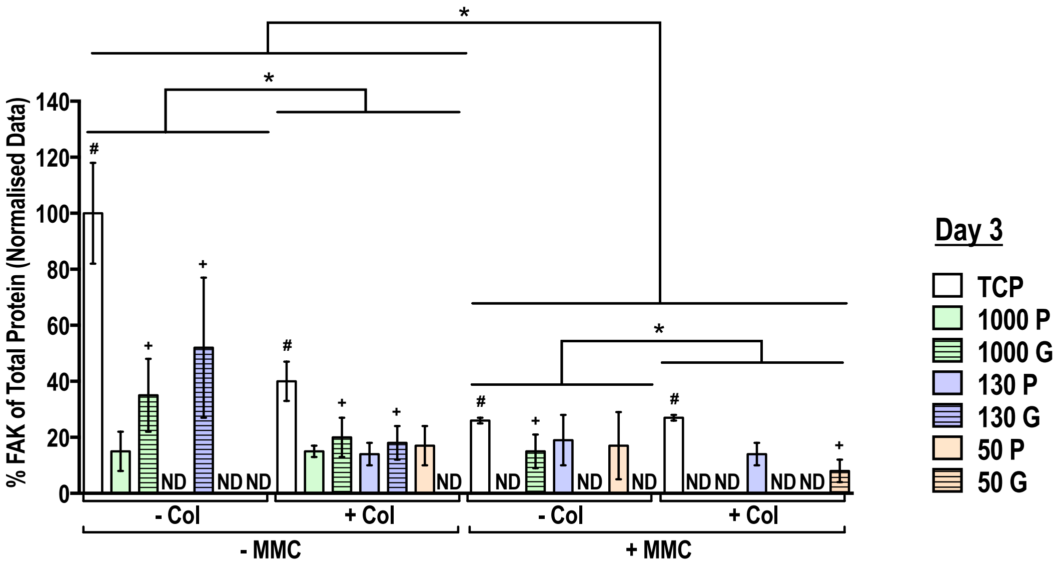

Supplementary Figure S10: Cell number on tissue culture plastic (TCP) without and with collagen type I coating (- Col, + Col) and macromolecular crowding (- MMC, + MMC) and on substrates of varying stiffness (1,000 kPa, 130 kPa, 50 kPa), surface topography [planar (P), grooved (G)], collagen type I coating (- Col, + Col) and macromolecular crowding (- MMC, + MMC) at day 3, day 7 and day 14. * indicates statistically significant difference ($p < 0.05$) between without and with collagen type I coating and between without and with MMC, # indicates statistical difference ($p < 0.05$) between TCP and PDMS substrates and + indicates statistical difference ($p < 0.05$) between planar and grooved topography.

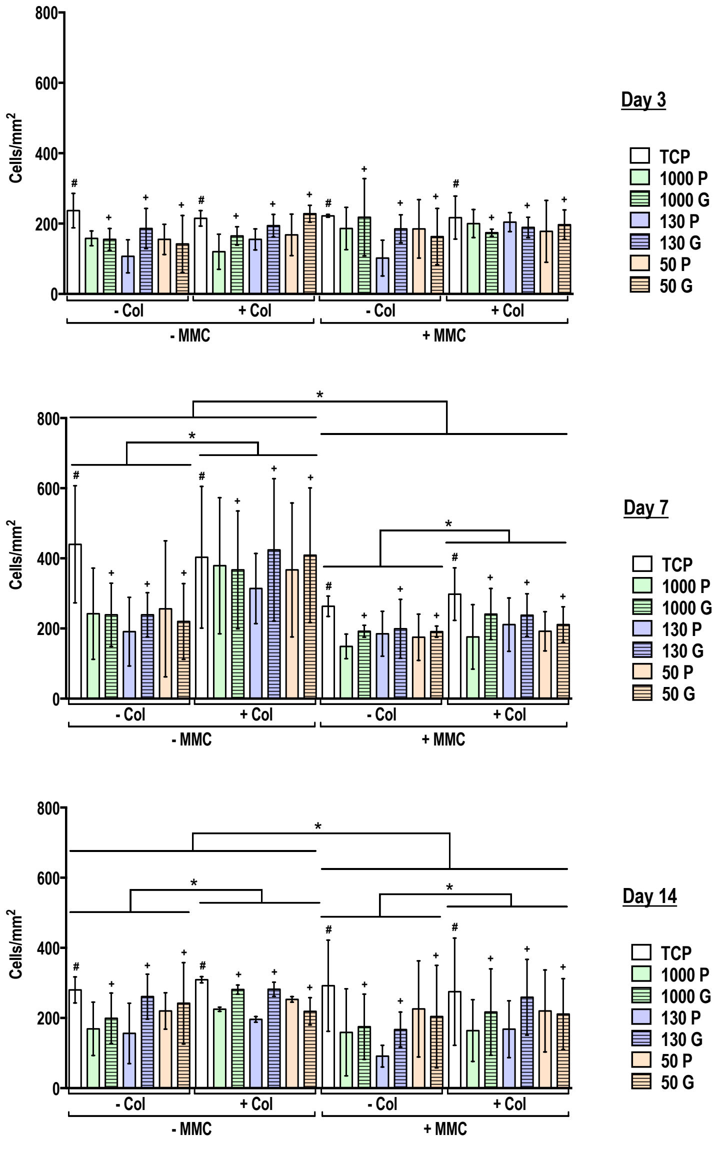

Supplementary Figure S11: Cell metabolic activity on tissue culture plastic (TCP) without and with collagen type I coating (- Col, + Col) and macromolecular crowding (- MMC, + MMC) and on substrates of varying stiffness (1,000 kPa, 130 kPa, 50 kPa), surface topography [planar (P), grooved (G)], collagen type I coating (- Col, + Col) and macromolecular crowding (- MMC, + MMC) at day 3, day 7 and day 14. * indicates statistically significant difference ($p < 0.05$) between without and with collagen type I coating and between without and with MMC, # indicates statistical difference ($p < 0.05$) between TCP and PDMS substrates and + indicates statistical difference ($p < 0.05$) between planar and grooved topography.

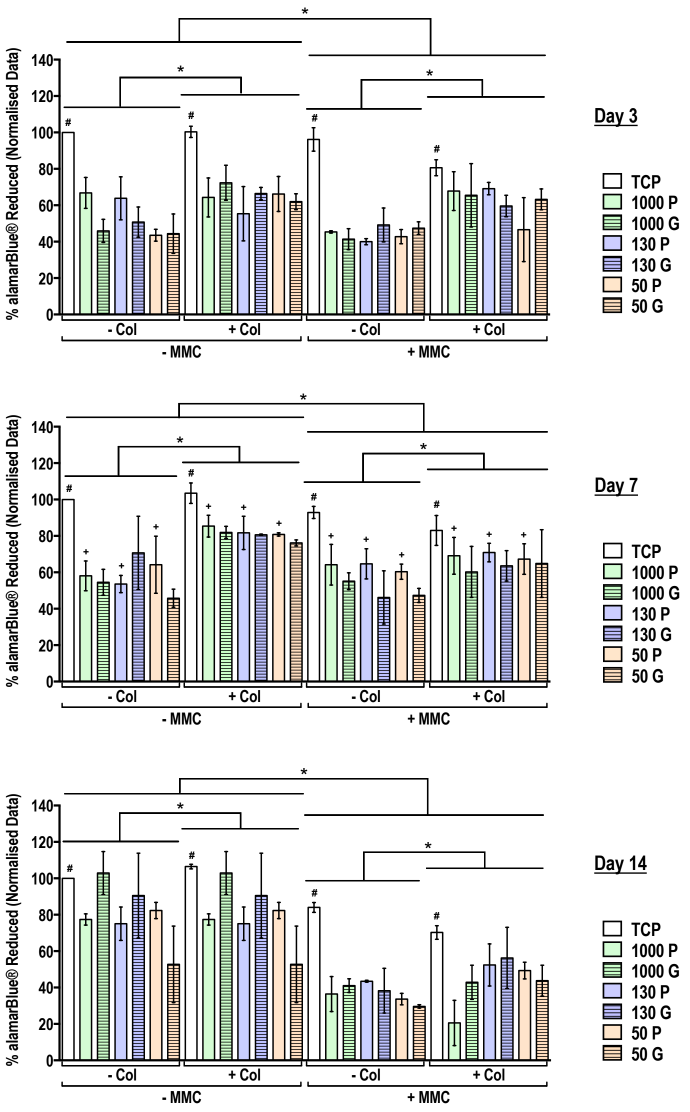

Supplementary Figure S12: Cell viability on tissue culture plastic (TCP) without and with collagen type I coating (- Col, + Col) and macromolecular crowding (- MMC, + MMC) and on substrates of varying stiffness (1,000 kPa, 130 kPa, 50 kPa), surface topography (planar, grooved), collagen type I coating (- Col, + Col) and macromolecular crowding (- MMC, + MMC) at day 3, day 7 and day 14, as shown using Live/Dead® assay. Live cells are represented in green and dead cells are represented in red. Cell viability was estimated at > 95 % for all conditions. Scale bar = 50 μm.

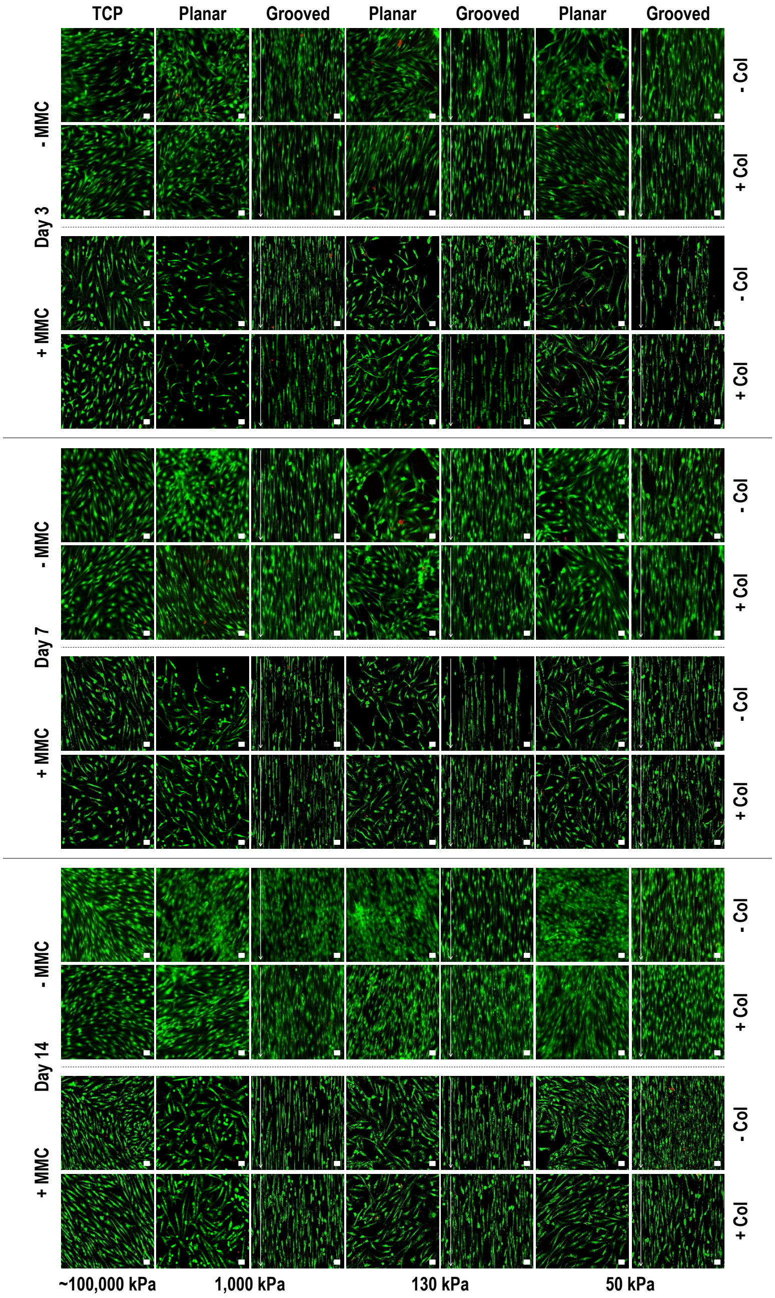

Supplementary Figure S13: Human tenocyte deposited collagen type I matrix and quantification of collagen type I matrix area deposited per cell at day 3 on tissue culture plastic (TCP) without and with collagen type I coating (- Col, + Col) and macromolecular crowding (- MMC, + MMC) and on substrates of varying stiffness (1,000 kPa, 130 kPa, 50 kPa), surface topography [planar (P), grooved (G)], collagen type I coating (- Col, + Col) and macromolecular crowding (- MMC, + MMC). Collagen type I is represented in orange. DAPI is represented in blue. Scale bar = 50 μm. * indicates statistically significant difference ($p < 0.05$) between without and with collagen type I coating and between without and with MMC, # indicates statistical difference ($p < 0.05$) between TCP and PDMS substrates and + indicates statistical difference ($p < 0.05$) between planar and grooved topography.

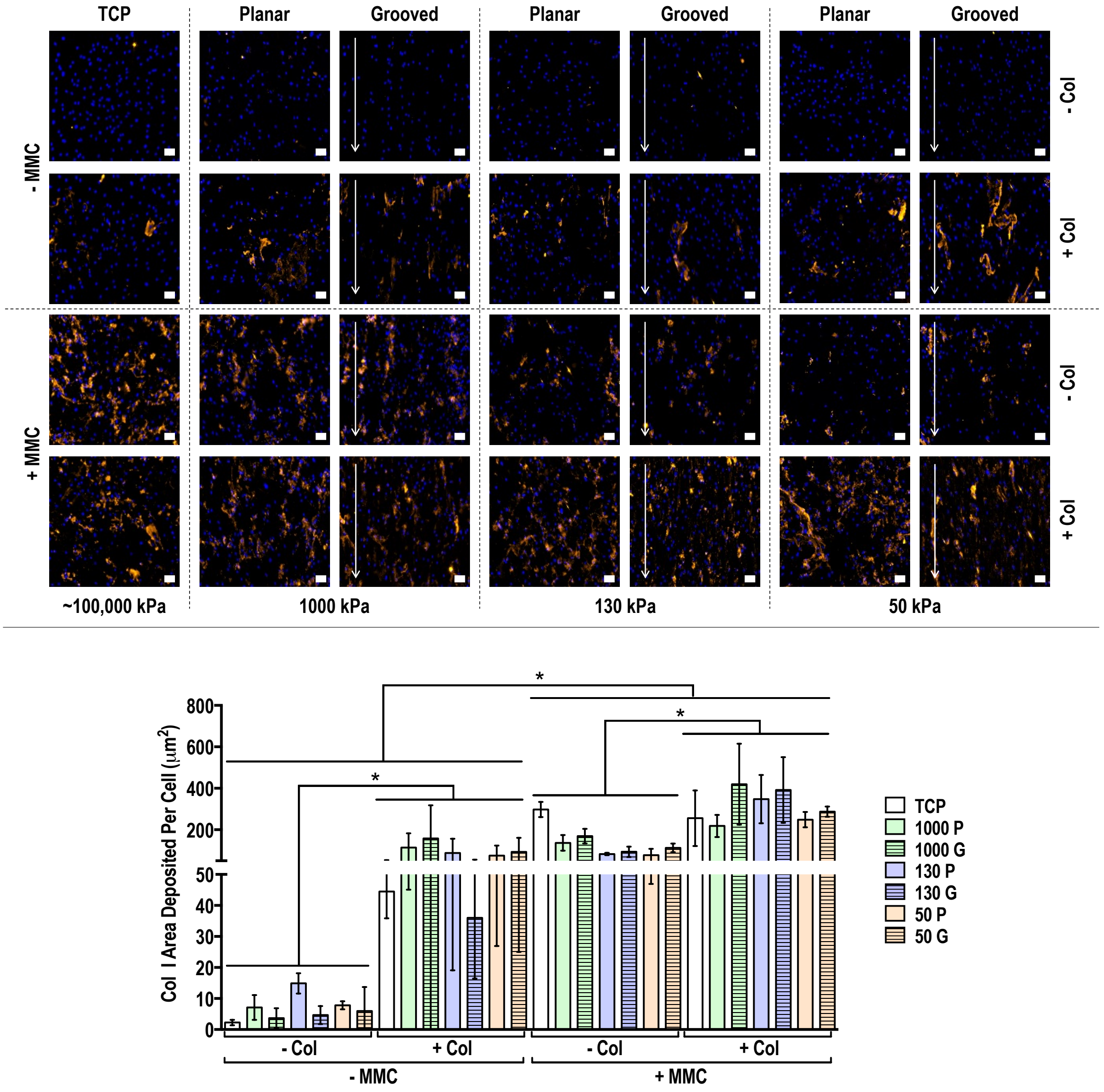
TCP
Planar
Grooved
Planar
Grooved
Planar
Grooved
- MMC
+ MMC
- Col
+ Col
- Col
+ Col
~100,000 kPa
1000 kPa
130 kPa
50 kPa
Col I Area Deposited Per Cell (μm²)
800
600
400
200
50
40
30
20
10
0
*
*
*
- Col
+ Col
- Col
+ Col
- MMC
+ MMC
TCP
1000 P
1000 G
130 P
130 G
50 P
50 G

Supplementary Figure S14: Human tenocyte deposited collagen type III matrix and quantification of collagen type III matrix area deposited per cell at day 3 on tissue culture plastic (TCP) without and with collagen type I coating (- Col, + Col) and macromolecular crowding (- MMC, + MMC) and on substrates of varying stiffness (1,000 kPa, 130 kPa, 50 kPa), surface topography [planar (P), grooved (G)], collagen type I coating (- Col, + Col) and macromolecular crowding (- MMC, + MMC). Collagen type III is represented in green. DAPI is represented in blue. Scale bar = 50 μm. * indicates statistically significant difference ($p < 0.05$) between without and with collagen type I coating and between without and with MMC, # indicates statistical difference ($p < 0.05$) between TCP and PDMS substrates and + indicates statistical difference ($p < 0.05$) between planar and grooved topography.

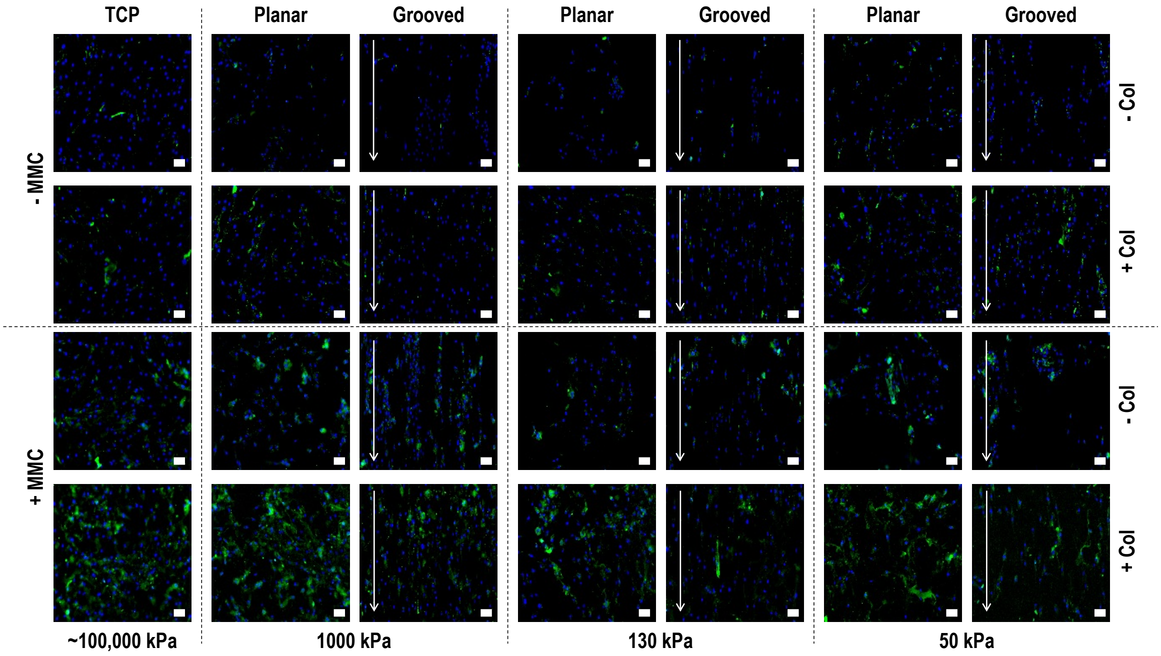

TCP
Planar
Grooved
Planar
Grooved
Planar
Grooved
- MMC
+ MMC
- Col
+ Col
- Col
+ Col
~100,000 kPa
1000 kPa
130 kPa
50 kPa

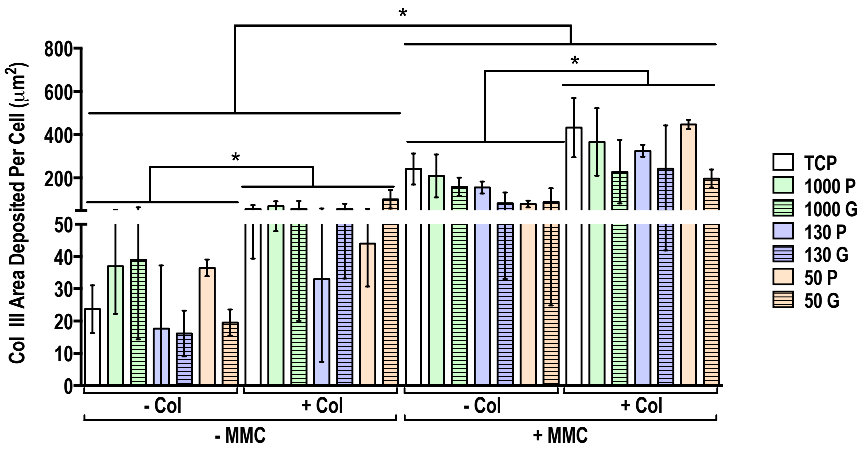

Col III Area Deposited Per Cell (μm²)
800
600
400
200
50
40
30
20
10
0
*
*
*
- Col
+ Col
- Col
+ Col
- MMC
+ MMC
TCP
1000 P
1000 G
130 P
130 G
50 P
50 G

Supplementary Figure S15: Human tenocyte deposited collagen type IV matrix and quantification of collagen type IV matrix area deposited per cell at day 3 on tissue culture plastic (TCP) without and with collagen type I coating (- Col, + Col) and macromolecular crowding (- MMC, + MMC) and on substrates of varying stiffness (1,000 kPa, 130 kPa, 50 kPa), surface topography [planar (P), grooved (G)], collagen type I coating (- Col, + Col) and macromolecular crowding (- MMC, + MMC). Collagen type IV is represented in red. DAPI is represented in blue. Scale bar = 50 μm. * indicates statistically significant difference (p < 0.05) between without and with collagen type I coating and between without and with MMC, # indicates statistical difference (p < 0.05) between TCP and PDMS substrates and + indicates statistical difference (p < 0.05) between planar and grooved topography.

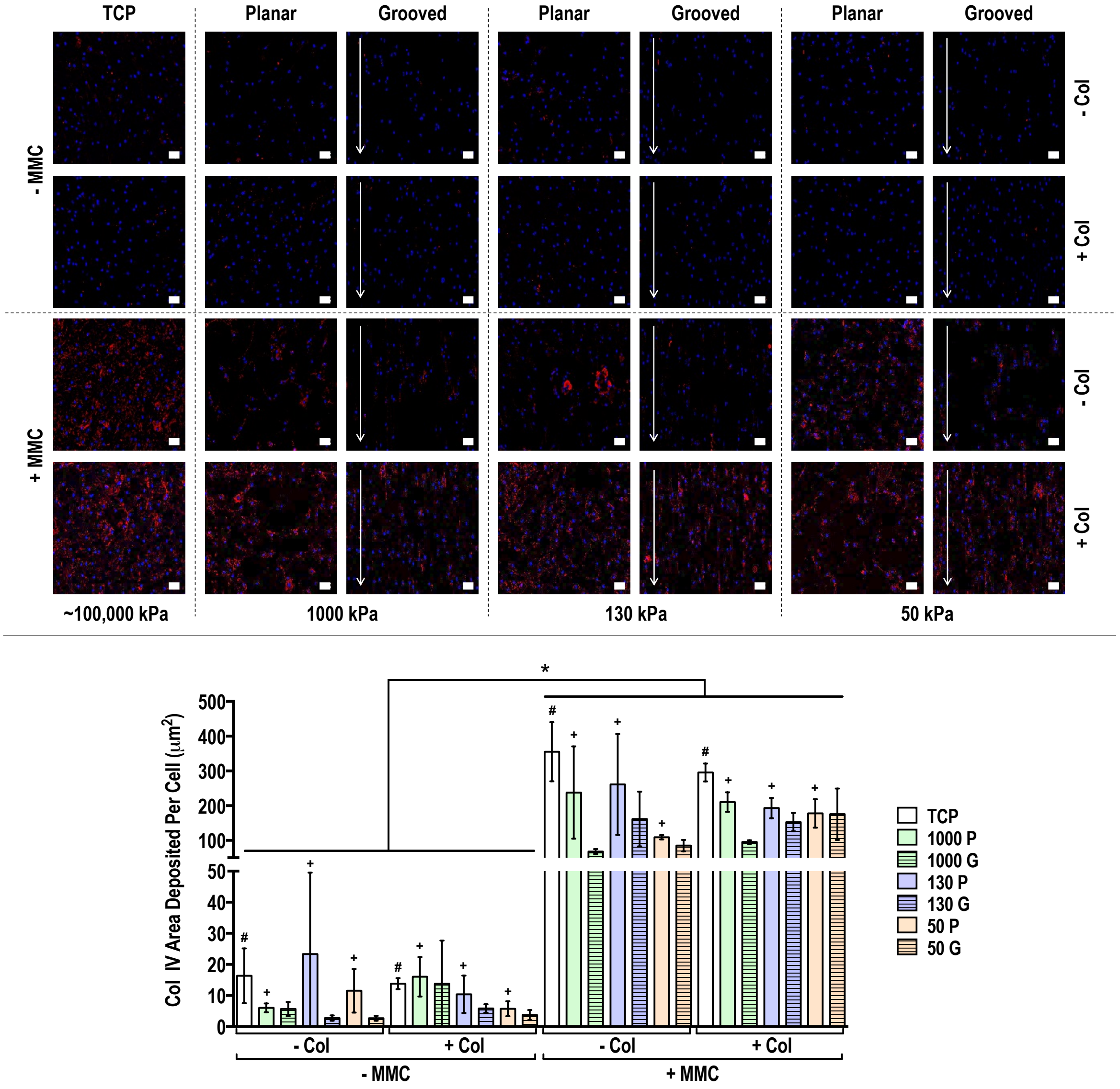
TCP
Planar
Grooved
Planar
Grooved
Planar
Grooved
- MMC
+ MMC
- Col
+ Col
- Col
+ Col
~100,000 kPa
1000 kPa
130 kPa
50 kPa
Col IV Area Deposited Per Cell (μm²)
500
400
300
200
100
50
40
30
20
10
0
*
#
+
- Col
+ Col
- Col
+ Col
- MMC
+ MMC
TCP
1000 P
1000 G
130 P
130 G
50 P
50 G

Supplementary Figure S16: Human tenocyte deposited collagen type V matrix and quantification of collagen type V matrix area deposited per cell at day 3 on tissue culture plastic (TCP) without and with collagen type I coating (- Col, + Col) and macromolecular crowding (- MMC, + MMC) and on substrates of varying stiffness (1,000 kPa, 130 kPa, 50 kPa), surface topography [planar (P), grooved (G)], collagen type I coating (- Col, + Col) and macromolecular crowding (- MMC, + MMC). Collagen type V is represented in green. DAPI is represented in blue. Scale bar = 50 μm. * indicates statistically significant difference (p < 0.05) between without and with collagen type I coating and between without and with MMC, # indicates statistical difference (p < 0.05) between TCP and PDMS substrates and + indicates statistical difference (p < 0.05) between planar and grooved topography.

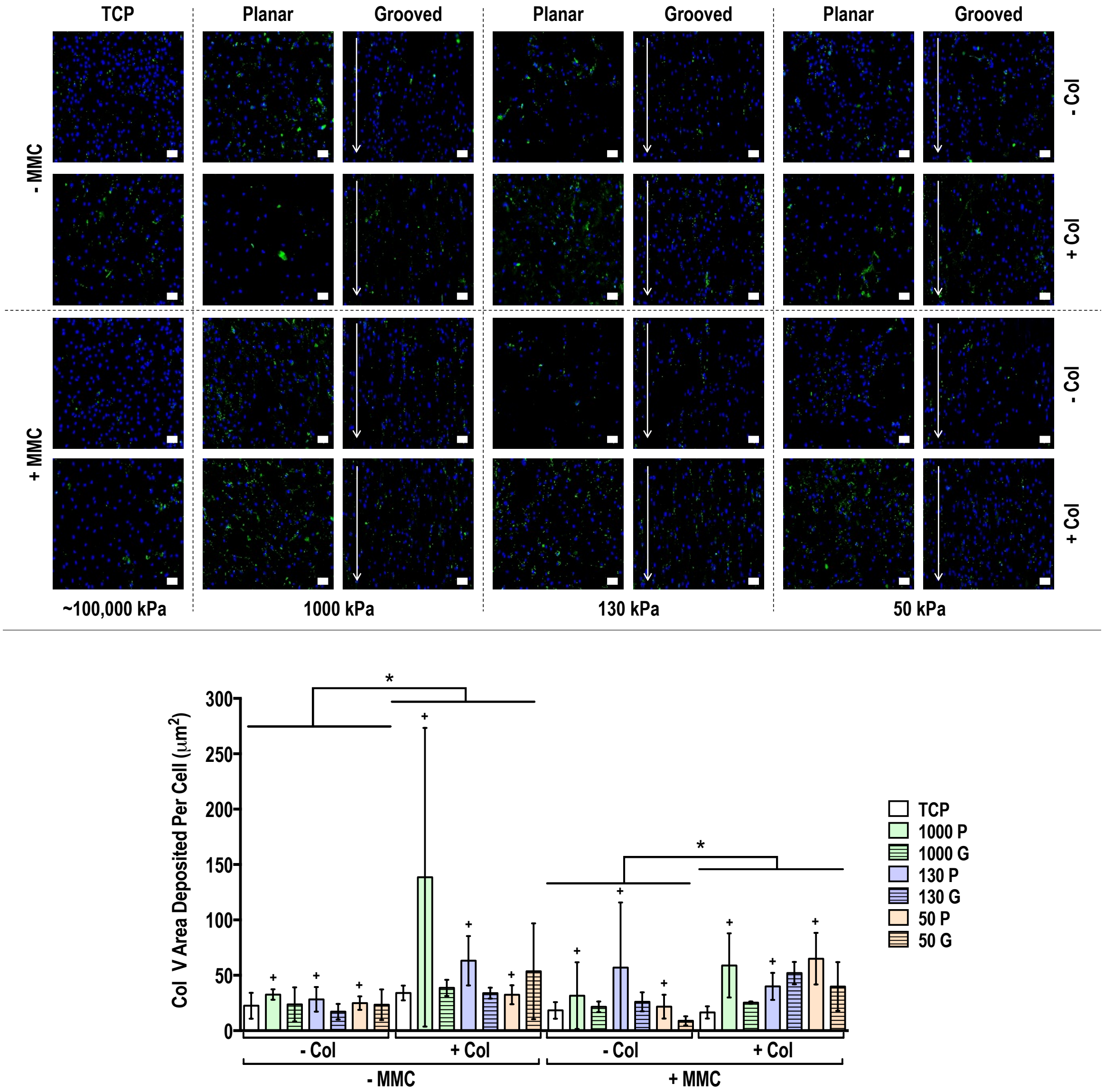
TCP
Planar
Grooved
Planar
Grooved
Planar
Grooved
- MMC
+ MMC
- Col
+ Col
- Col
+ Col
~100,000 kPa
1000 kPa
130 kPa
50 kPa
Col V Area Deposited Per Cell (μm²)
300
250
200
150
100
50
0
*
+
- Col
+ Col
- Col
+ Col
- MMC
+ MMC
TCP
1000 P
1000 G
130 P
130 G
50 P
50 G

Supplementary Figure S17: Human tenocyte deposited collagen type VI matrix and quantification of collagen type VI matrix area deposited per cell at day 3 on tissue culture plastic (TCP) without and with collagen type I coating (- Col, + Col) and macromolecular crowding (- MMC, + MMC) and on substrates of varying stiffness (1,000 kPa, 130 kPa, 50 kPa), surface topography [planar (P), grooved (G)], collagen type I coating (- Col, + Col) and macromolecular crowding (- MMC, + MMC). Collagen type VI is represented in red. DAPI is represented in blue. Scale bar = 50 μm. * indicates statistically significant difference (p < 0.05) between without and with collagen type I coating and between without and with MMC, # indicates statistical difference (p < 0.05) between TCP and PDMS substrates and + indicates statistical difference (p < 0.05) between planar and grooved topography.

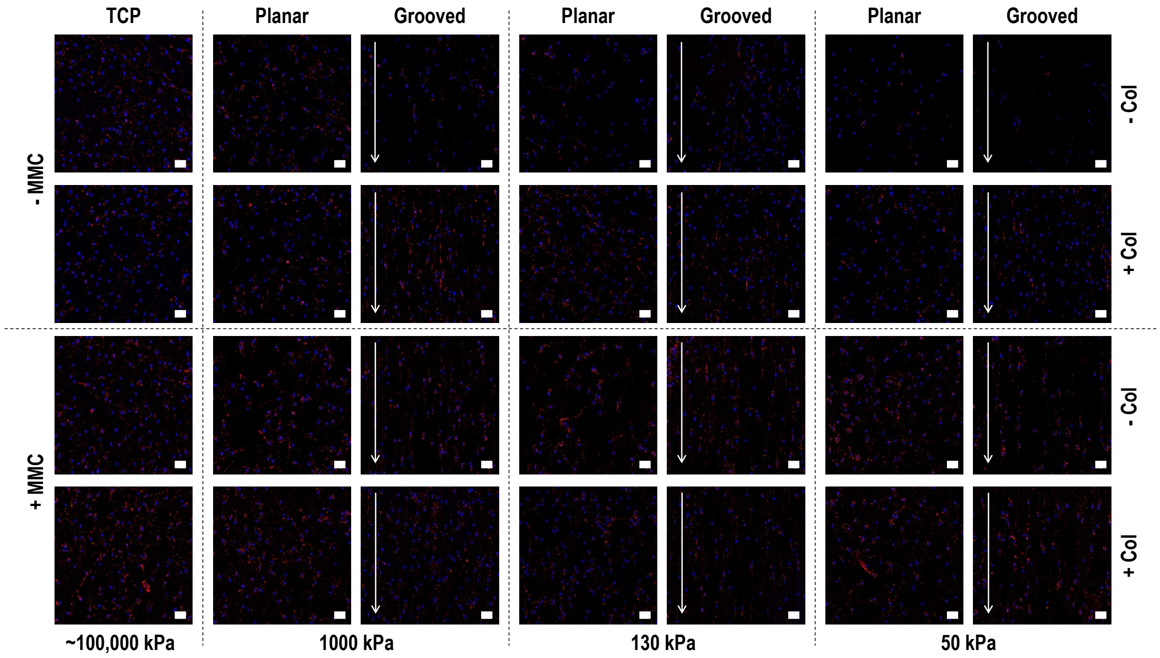

TCP
Planar
Grooved
Planar
Grooved
Planar
Grooved
- MMC
+ MMC
- Col
+ Col
- Col
+ Col
~100,000 kPa
1000 kPa
130 kPa
50 kPa

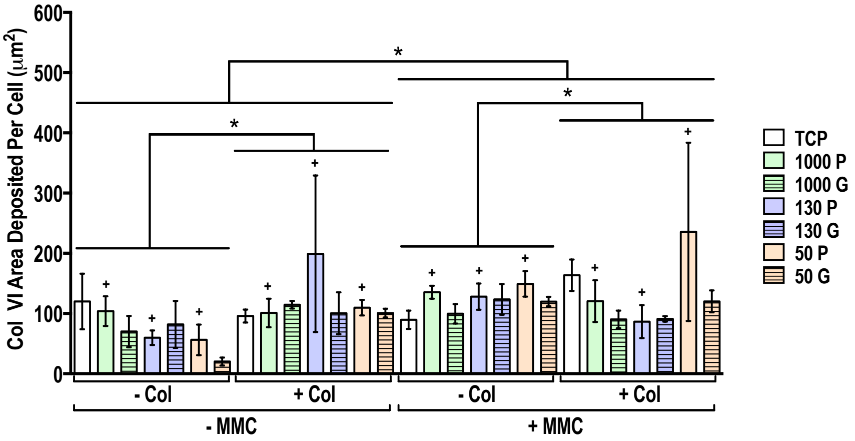

Col VI Area Deposited Per Cell (μm^2)
600
500
400
300
200
100
0
- Col
+ Col
- Col
+ Col
- MMC
+ MMC
TCP
1000 P
1000 G
130 P
130 G
50 P
50 G

Supplementary Figure S18: Human tenocyte deposited fibronectin matrix and quantification of fibronectin matrix area deposited per cell at day 3 on tissue culture plastic (TCP) without and with collagen type I coating (- Col, + Col) and macromolecular crowding (- MMC, + MMC) and on substrates of varying stiffness (1,000 kPa, 130 kPa, 50 kPa), surface topography [planar (P), grooved (G)], collagen type I coating (- Col, + Col) and macromolecular crowding (- MMC, + MMC). Fibronectin is represented in green. DAPI is represented in blue. Scale bar = 50 μm. * indicates statistically significant difference ($p < 0.05$) between without and with collagen type I coating and between without and with MMC, # indicates statistical difference ($p < 0.05$) between TCP and PDMS substrates and + indicates statistical difference ($p < 0.05$) between planar and grooved topography.

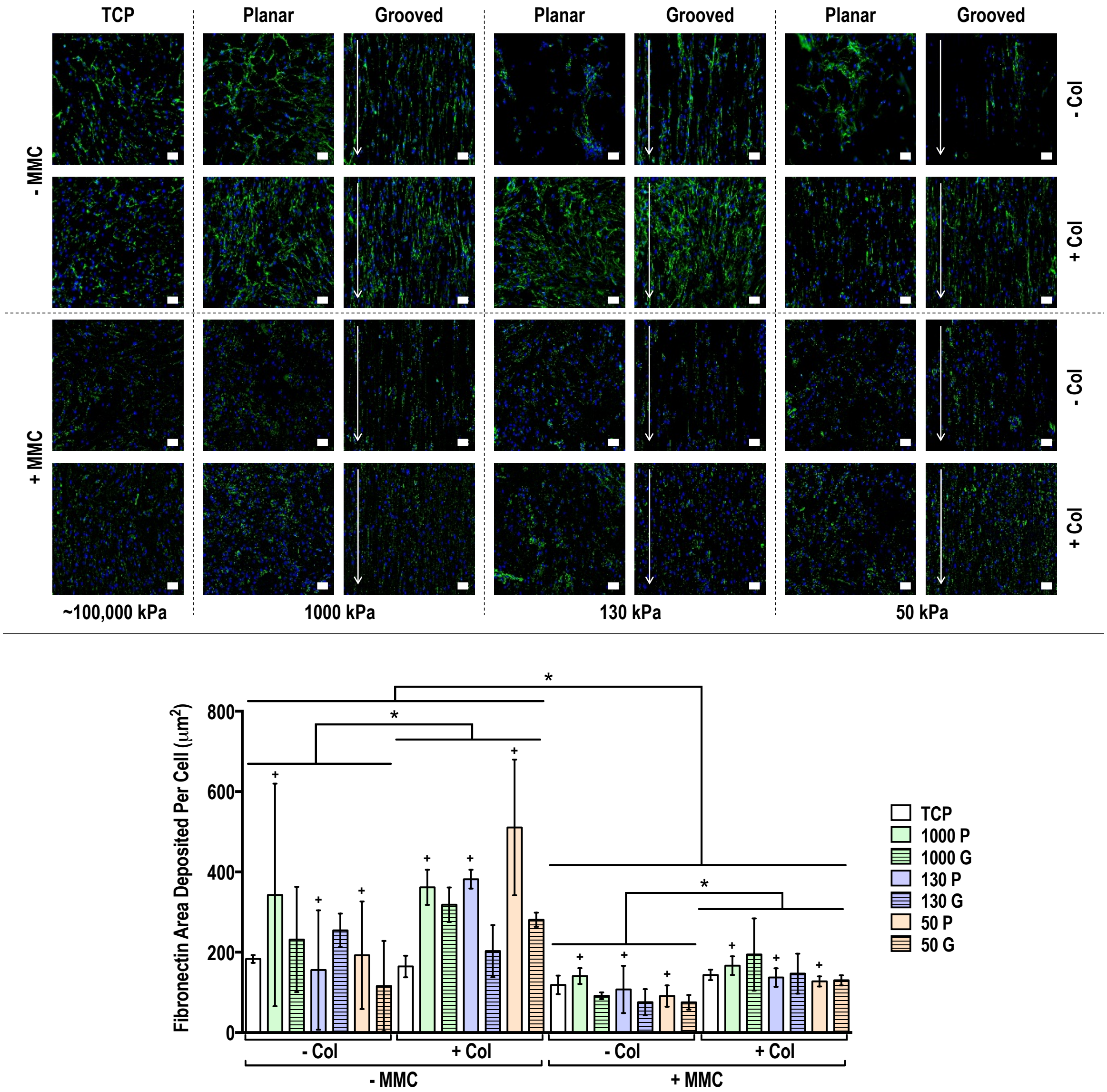
TCP
Planar
Grooved
Planar
Grooved
Planar
Grooved
- MMC
+ MMC
- Col
+ Col
- Col
+ Col
~100,000 kPa
1000 kPa
130 kPa
50 kPa
Fibronectin Area Deposited Per Cell (μm2)
800
600
400
200
0
*
*
*
- Col
+ Col
- Col
+ Col
- MMC
+ MMC
TCP
1000 P
1000 G
130 P
130 G
50 P
50 G

Supplementary Figure S19: Human tenocyte deposited collagen type I matrix and quantification of collagen type I matrix area deposited per cell at day 7 on tissue culture plastic (TCP) without and with collagen type I coating (- Col, + Col) and macromolecular crowding (- MMC, + MMC) and on substrates of varying stiffness (1,000 kPa, 130 kPa, 50 kPa), surface topography [planar (P), grooved (G)], collagen type I coating (- Col, + Col) and macromolecular crowding (- MMC, + MMC). Collagen type I is represented in orange. DAPI is represented in blue. Scale bar = 50 μm. * indicates statistically significant difference (p < 0.05) between without and with collagen type I coating and between without and with MMC, # indicates statistical difference (p < 0.05) between TCP and PDMS substrates and + indicates statistical difference (p < 0.05) between planar and grooved topography.

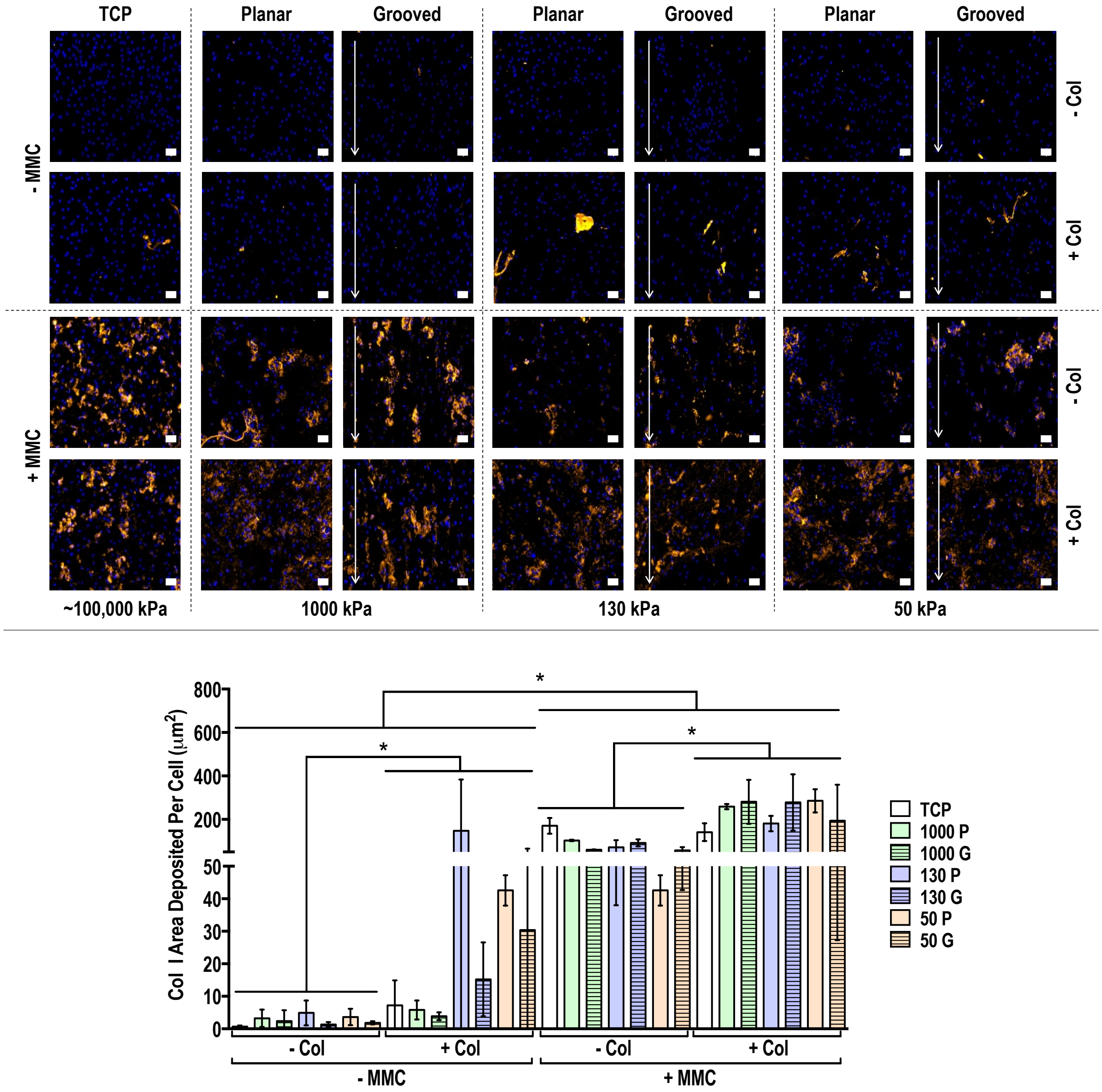
TCP
Planar
Grooved
Planar
Grooved
Planar
Grooved
- MMC
+ MMC
- Col
+ Col
- Col
+ Col
~100,000 kPa
1000 kPa
130 kPa
50 kPa
Col I Area Deposited Per Cell (μm²)
800
600
400
200
50
40
30
20
10
0
*
*
*
- Col
+ Col
- Col
+ Col
- MMC
+ MMC
TCP
1000 P
1000 G
130 P
130 G
50 P
50 G

Supplementary Figure S20: Human tenocyte deposited collagen type III matrix and quantification of collagen type III matrix area deposited per cell at day 7 on tissue culture plastic (TCP) without and with collagen type I coating (- Col, + Col) and macromolecular crowding (- MMC, + MMC) and on substrates of varying stiffness (1,000 kPa, 130 kPa, 50 kPa), surface topography [planar (P), grooved (G)], collagen type I coating (- Col, + Col) and macromolecular crowding (- MMC, + MMC). Collagen type III is represented in green. DAPI is represented in blue. Scale bar = 50 μm. * indicates statistically significant difference ($p < 0.05$) between without and with collagen type I coating and between without and with MMC, # indicates statistical difference ($p < 0.05$) between TCP and PDMS substrates and + indicates statistical difference ($p < 0.05$) between planar and grooved topography.

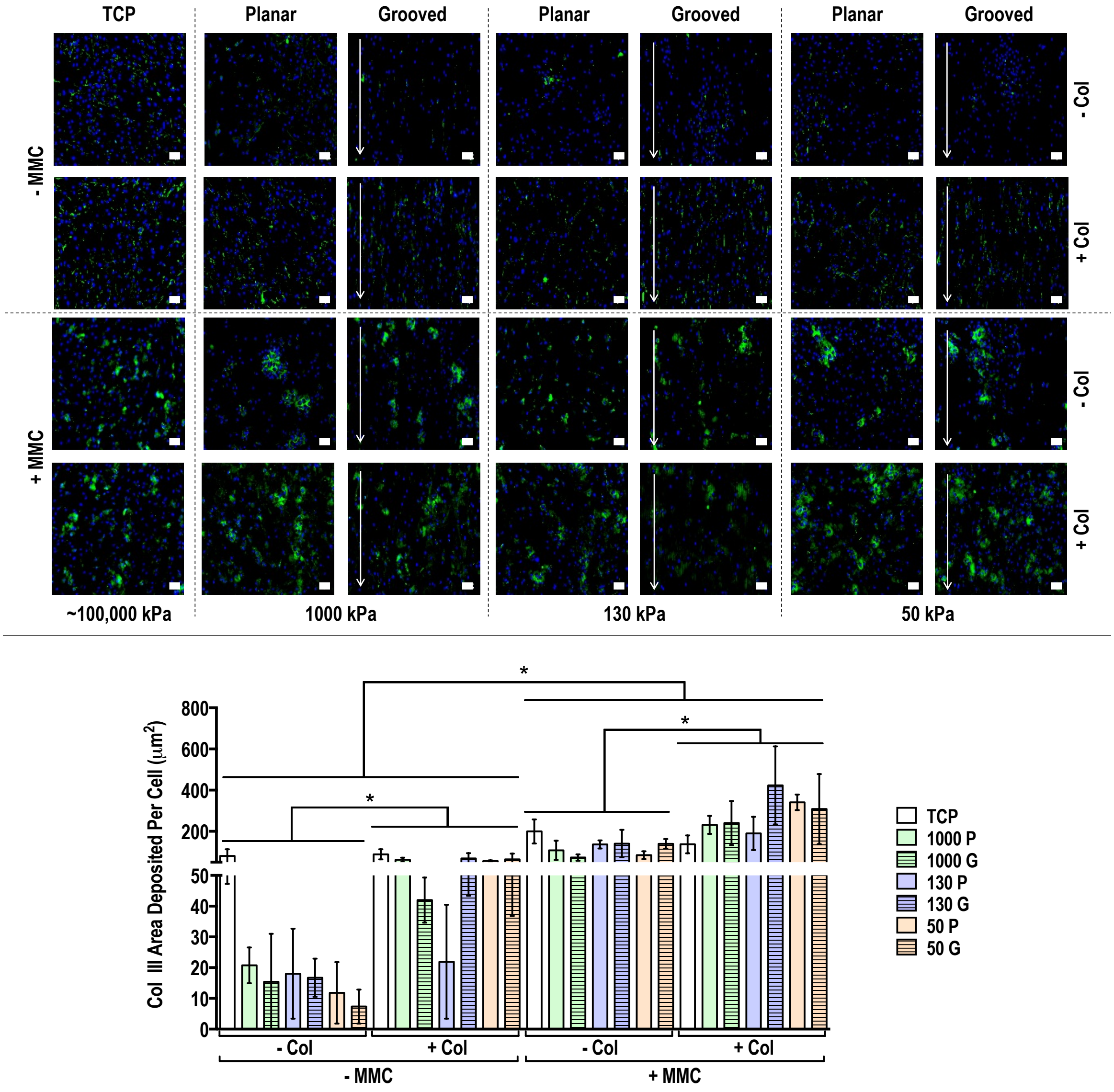
TCP
Planar
Grooved
Planar
Grooved
Planar
Grooved
- MMC
+ MMC
- Col
+ Col
- Col
+ Col
~100,000 kPa
1000 kPa
130 kPa
50 kPa
Col III Area Deposited Per Cell (μm²)
800
600
400
200
50
40
30
20
10
0
*
*
*
- Col
+ Col
- Col
+ Col
- MMC
+ MMC
TCP
1000 P
1000 G
130 P
130 G
50 P
50 G

Supplementary Figure S21: Human tenocyte deposited collagen type IV matrix and quantification of collagen type IV matrix area deposited per cell at day 7 on tissue culture plastic (TCP) without and with collagen type I coating (- Col, + Col) and macromolecular crowding (- MMC, + MMC) and on substrates of varying stiffness (1,000 kPa, 130 kPa, 50 kPa), surface topography [planar (P), grooved (G)], collagen type I coating (- Col, + Col) and macromolecular crowding (- MMC, + MMC). Collagen type IV is represented in red. DAPI is represented in blue. Scale bar = 50 μm. * indicates statistically significant difference ($p < 0.05$) between without and with collagen type I coating and between without and with MMC, # indicates statistical difference ($p < 0.05$) between TCP and PDMS substrates and + indicates statistical difference ($p < 0.05$) between planar and grooved topography.

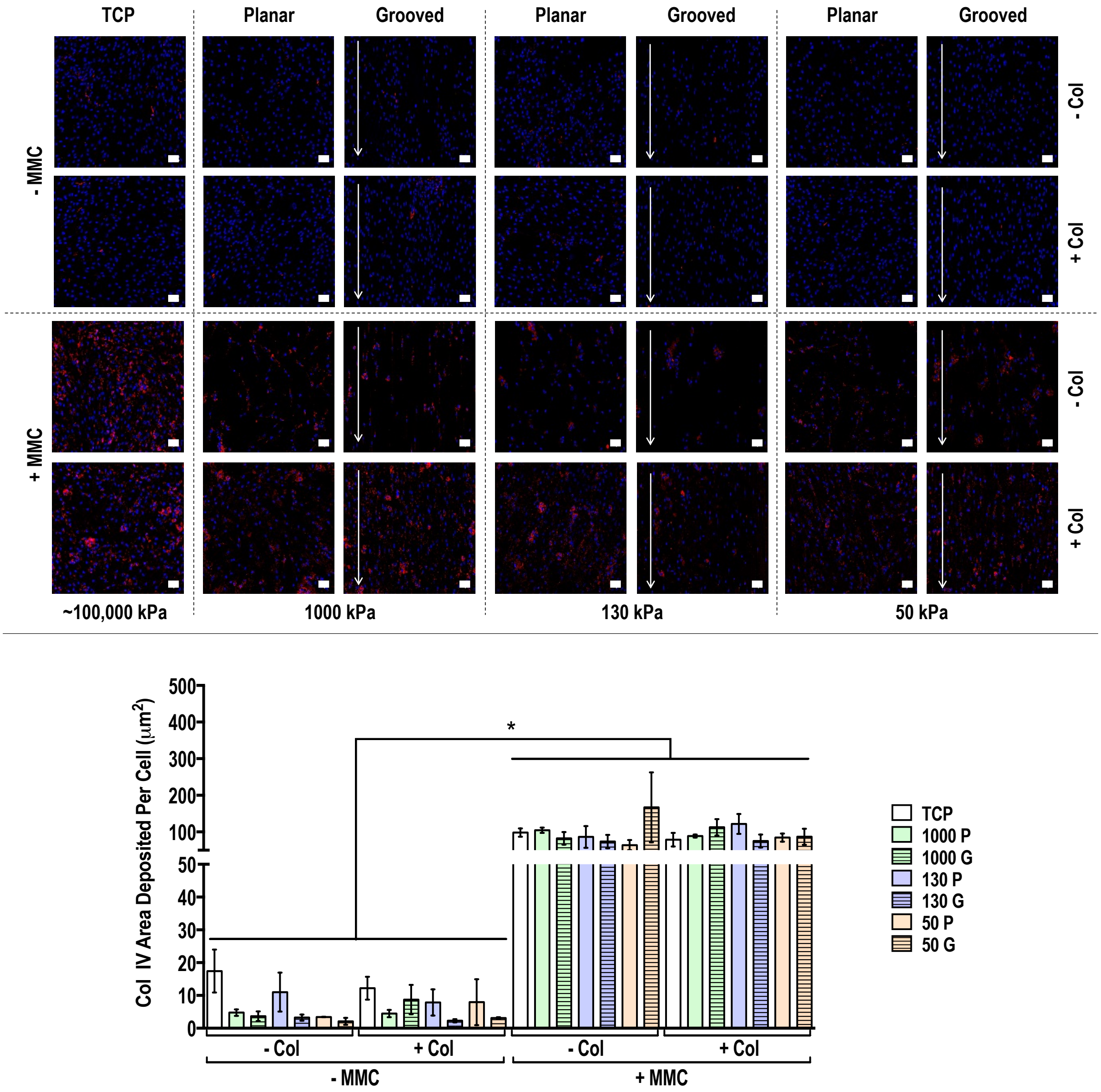

TCP
Planar
Grooved
Planar
Grooved
Planar
Grooved
- MMC
+ MMC
- Col
+ Col
- Col
+ Col
~100,000 kPa
1000 kPa
130 kPa
50 kPa
Col IV Area Deposited Per Cell (μm2)
500
400
300
200
100
50
40
30
20
10
0
*
TCP
1000 P
1000 G
130 P
130 G
50 P
50 G
- Col
+ Col
- Col
+ Col
- MMC
+ MMC

Supplementary Figure S22: Human tenocyte deposited collagen type V matrix and quantification of collagen type V matrix area deposited per cell at day 7 on tissue culture plastic (TCP) without and with collagen type I coating (- Col, + Col) and macromolecular crowding (- MMC, + MMC) and on substrates of varying stiffness (1,000 kPa, 130 kPa, 50 kPa), surface topography [planar (P), grooved (G)], collagen type I coating (- Col, + Col) and macromolecular crowding (- MMC, + MMC). Collagen type V is represented in green. DAPI is represented in blue. Scale bar = 50 μm. * indicates statistically significant difference ($p < 0.05$) between without and with collagen type I coating and between without and with MMC, # indicates statistical difference ($p < 0.05$) between TCP and PDMS substrates and + indicates statistical difference ($p < 0.05$) between planar and grooved topography.

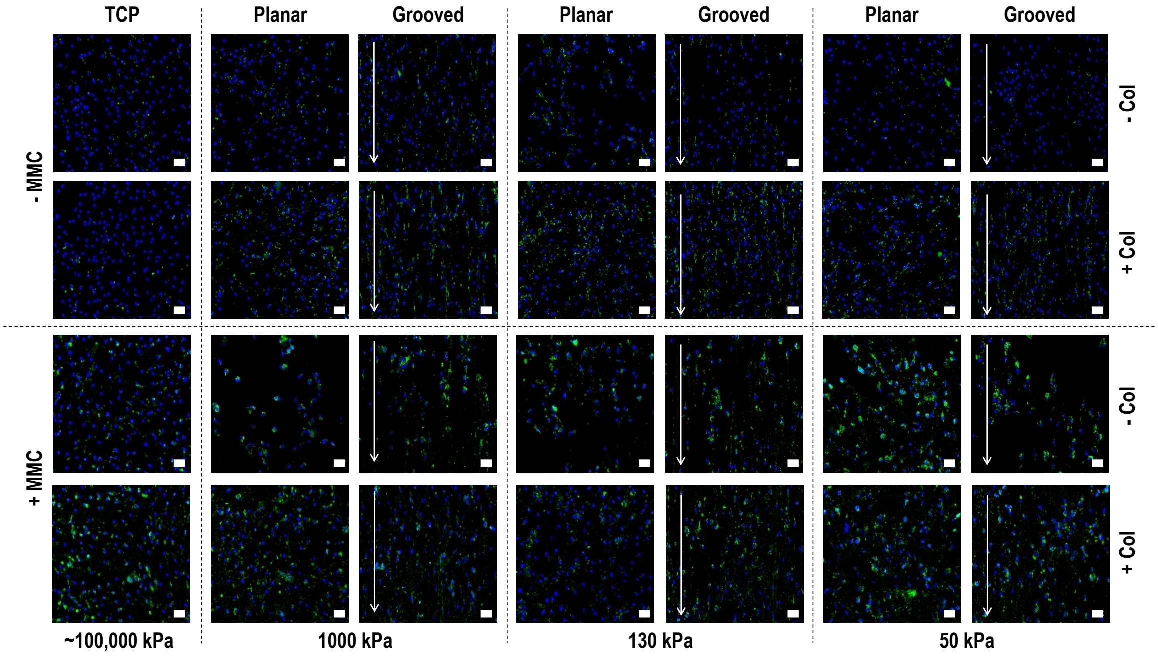
TCP
Planar
Grooved
Planar
Grooved
Planar
Grooved
- MMC
+ MMC
- Col
+ Col
- Col
+ Col
~100,000 kPa
1000 kPa
130 kPa
50 kPa

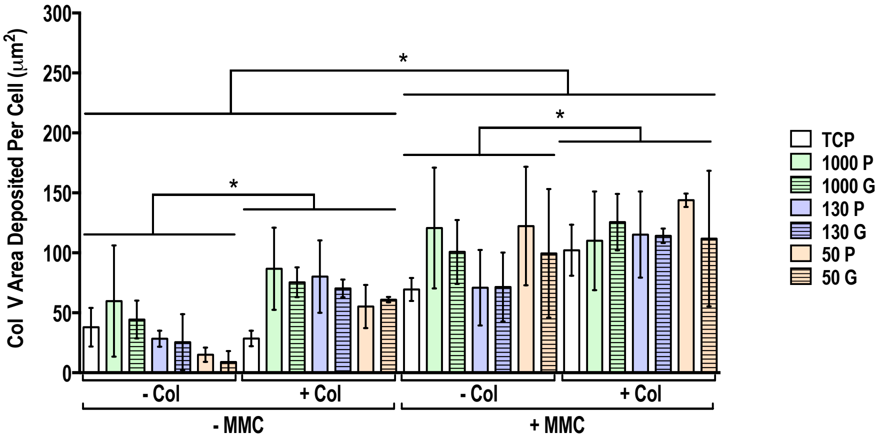
Col V Area Deposited Per Cell (μm²)
300
250
200
150
100
50
0
*
*
*
- Col
+ Col
- Col
+ Col
- MMC
+ MMC
TCP
1000 P
1000 G
130 P
130 G
50 P
50 G

Supplementary Figure S23: Human tenocyte deposited collagen type VI matrix and quantification of collagen type VI matrix area deposited per cell at day 7 on tissue culture plastic (TCP) without and with collagen type I coating (- Col, + Col) and macromolecular crowding (- MMC, + MMC) and on substrates of varying stiffness (1,000 kPa, 130 kPa, 50 kPa), surface topography [planar (P), grooved (G)], collagen type I coating (- Col, + Col) and macromolecular crowding (- MMC, + MMC). Collagen type VI is represented in red. DAPI is represented in blue. Scale bar = 50 μm. * indicates statistically significant difference ($p < 0.05$) between without and with collagen type I coating and between without and with MMC, # indicates statistical difference ($p < 0.05$) between TCP and PDMS substrates and + indicates statistical difference ($p < 0.05$) between planar and grooved topography.

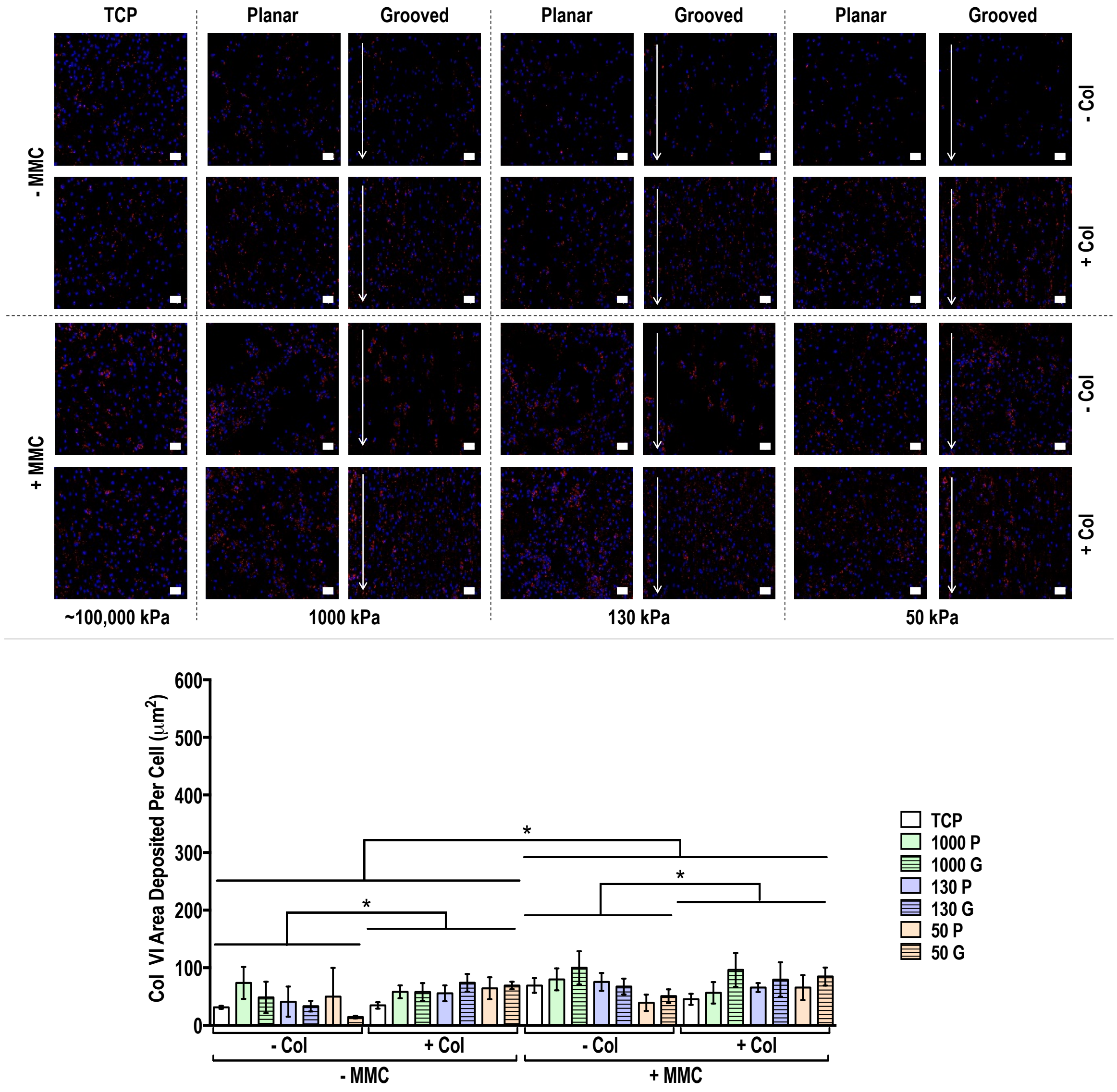

TCP
Planar
Grooved
Planar
Grooved
Planar
Grooved
- MMC
+ MMC
- Col
+ Col
- Col
+ Col
~100,000 kPa
1000 kPa
130 kPa
50 kPa
Col VI Area Deposited Per Cell (μm2)
600
500
400
300
200
100
0
*
*
*
- Col
+ Col
- Col
+ Col
- MMC
+ MMC
TCP
1000 P
1000 G
130 P
130 G
50 P
50 G

Supplementary Figure S24: Human tenocyte deposited fibronectin matrix and quantification of fibronectin matrix area deposited per cell at day 7 on tissue culture plastic (TCP) without and with collagen type I coating (- Col, + Col) and macromolecular crowding (- MMC, + MMC) and on substrates of varying stiffness (1,000 kPa, 130 kPa, 50 kPa), surface topography [planar (P), grooved (G)], collagen type I coating (- Col, + Col) and macromolecular crowding (- MMC, + MMC). Fibronectin is represented in green. DAPI is represented in blue. Scale bar = 50 μm. * indicates statistically significant difference ($p < 0.05$) between without and with collagen type I coating and between without and with MMC, # indicates statistical difference ($p < 0.05$) between TCP and PDMS substrates and + indicates statistical difference ($p < 0.05$) between planar and grooved topography.

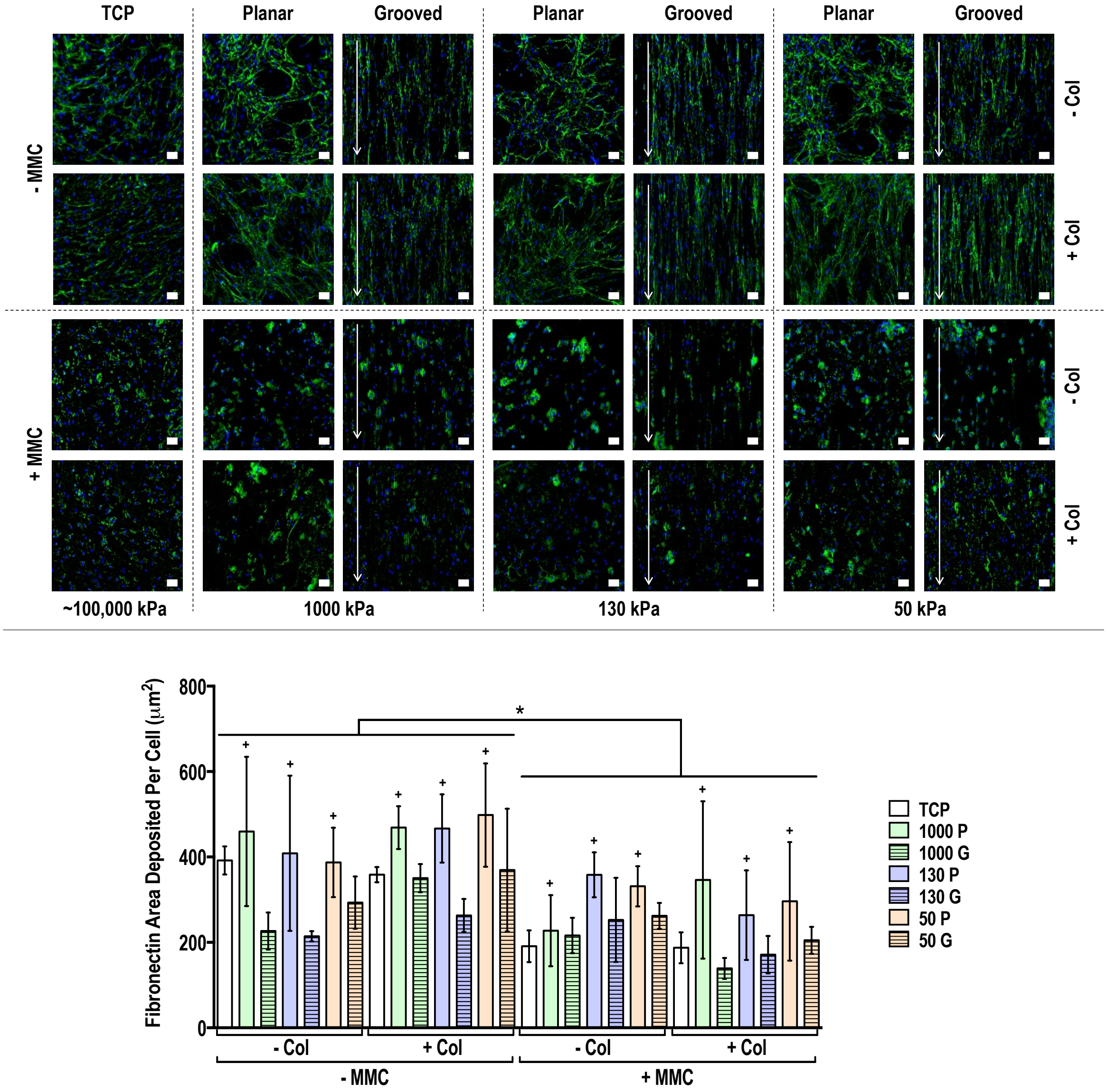
TCP
Planar
Grooved
Planar
Grooved
Planar
Grooved
- MMC
+ MMC
- Col
+ Col
- Col
+ Col
~100,000 kPa
1000 kPa
130 kPa
50 kPa
Fibronectin Area Deposited Per Cell (μm²)
800
600
400
200
0
*
+
- Col
+ Col
- Col
+ Col
- MMC
+ MMC
TCP
1000 P
1000 G
130 P
130 G
50 P
50 G

Supplementary Figure S25: Human tenocyte collagen type I matrix orientation at day 3, day 7 and day 14 on tissue culture plastic (TCP) without and with collagen type I coating (- Col, + Col) and macromolecular crowding (- MMC, + MMC) and on substrates of varying stiffness (1,000 kPa, 130 kPa, 50 kPa), surface topography [planar (P), grooved (G)], collagen type I coating (- Col, + Col) and macromolecular crowding (- MMC, + MMC). ND indicates conditions that did not produce sufficient matrix for analysis.

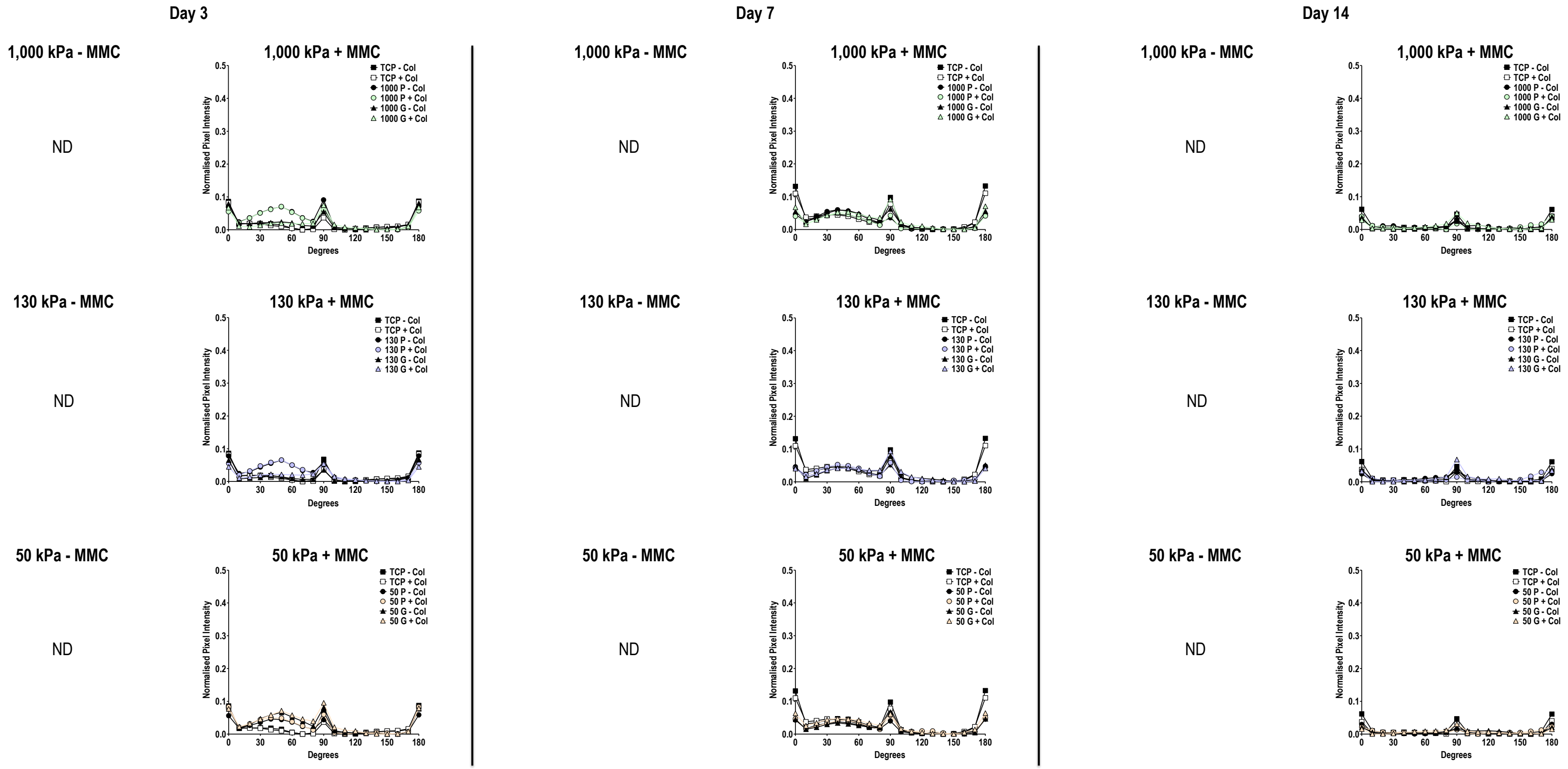

Supplementary Figure S26: Human tenocyte collagen type III matrix orientation at day 3, day 7 and day 14 on tissue culture plastic (TCP) without and with collagen type I coating (- Col, + Col) and macromolecular crowding (- MMC, + MMC) and on substrates of varying stiffness (1,000 kPa, 130 kPa, 50 kPa), surface topography [planar (P), grooved (G)], collagen type I coating (- Col, + Col) and macromolecular crowding (- MMC, + MMC).

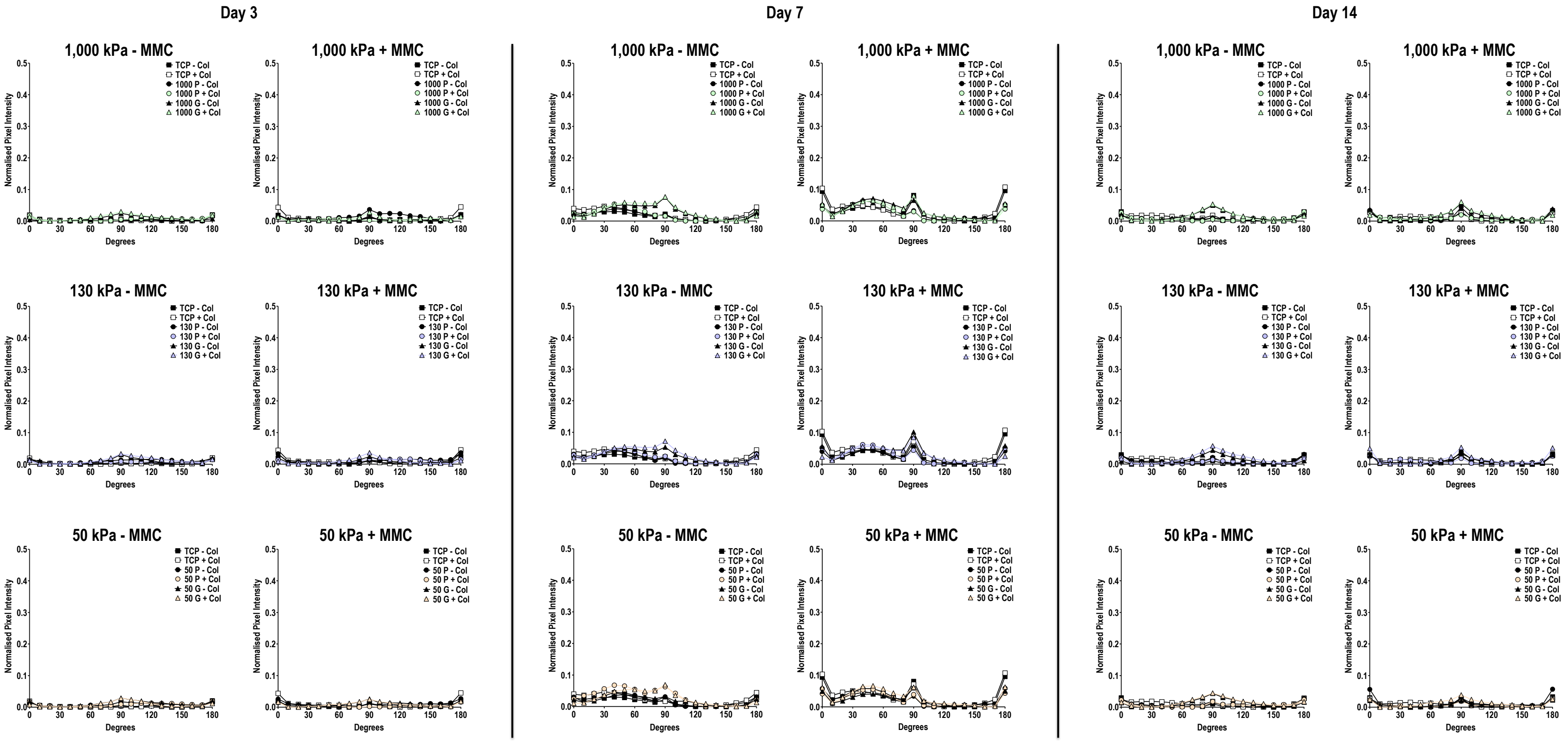

Supplementary Figure S27: Human tenocyte collagen type IV matrix orientation at day 3, day 7 and day 14 on tissue culture plastic (TCP) without and with collagen type I coating (- Col, + Col) and macromolecular crowding (- MMC, + MMC) and on substrates of varying stiffness (1,000 kPa, 130 kPa, 50 kPa), surface topography [planar (P), grooved (G)], collagen type I coating (- Col, + Col) and macromolecular crowding (- MMC, + MMC). ND indicates conditions that did not produce sufficient matrix for analysis.

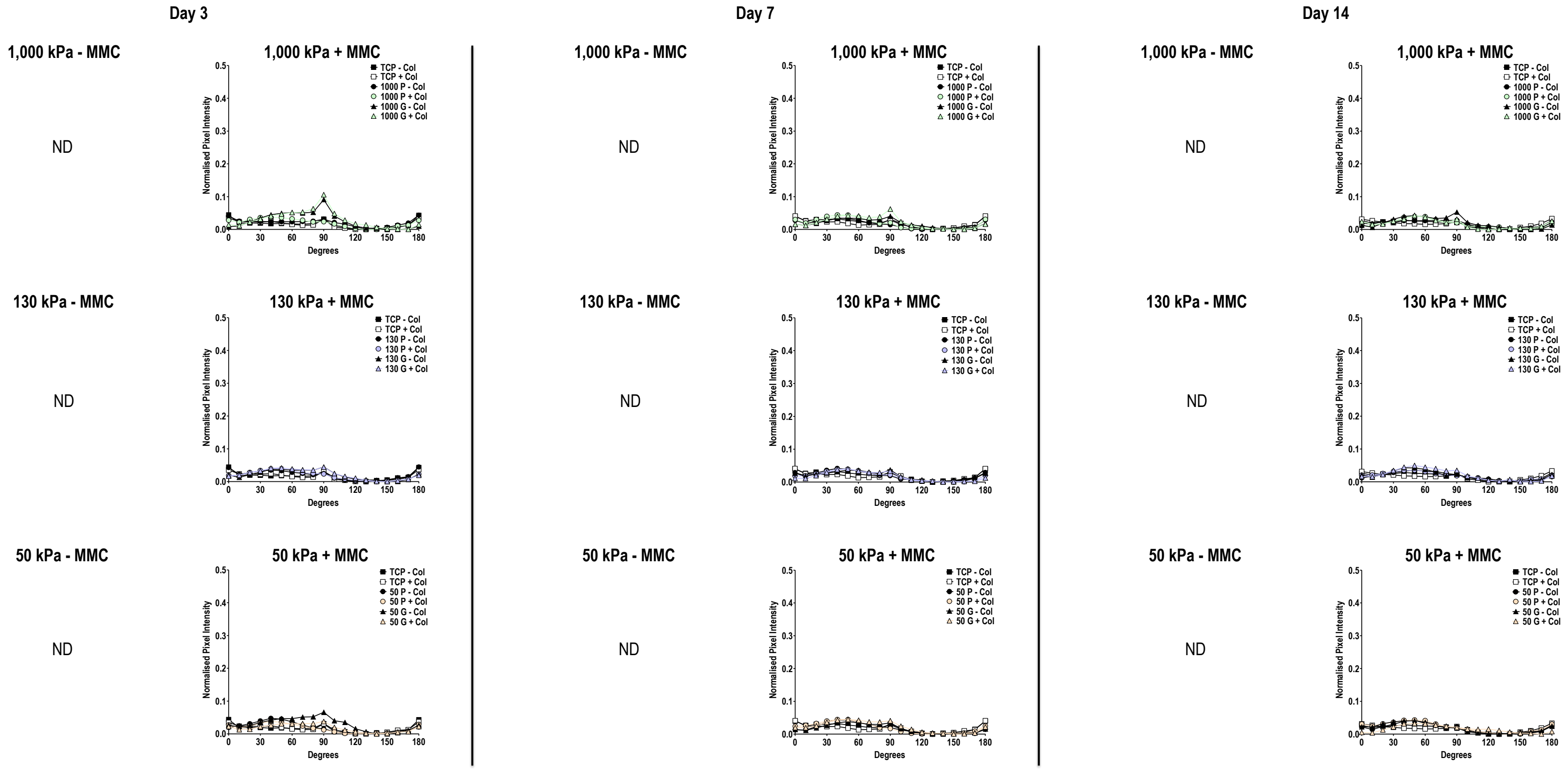

Supplementary Figure S28: Human tenocyte collagen type V matrix orientation at day 3, day 7 and day 14 on tissue culture plastic (TCP) without and with collagen type I coating (- Col, + Col) and macromolecular crowding (- MMC, + MMC) and on substrates of varying stiffness (1,000 kPa, 130 kPa, 50 kPa), surface topography [planar (P), grooved (G)], collagen type I coating (- Col, + Col) and macromolecular crowding (- MMC, + MMC).

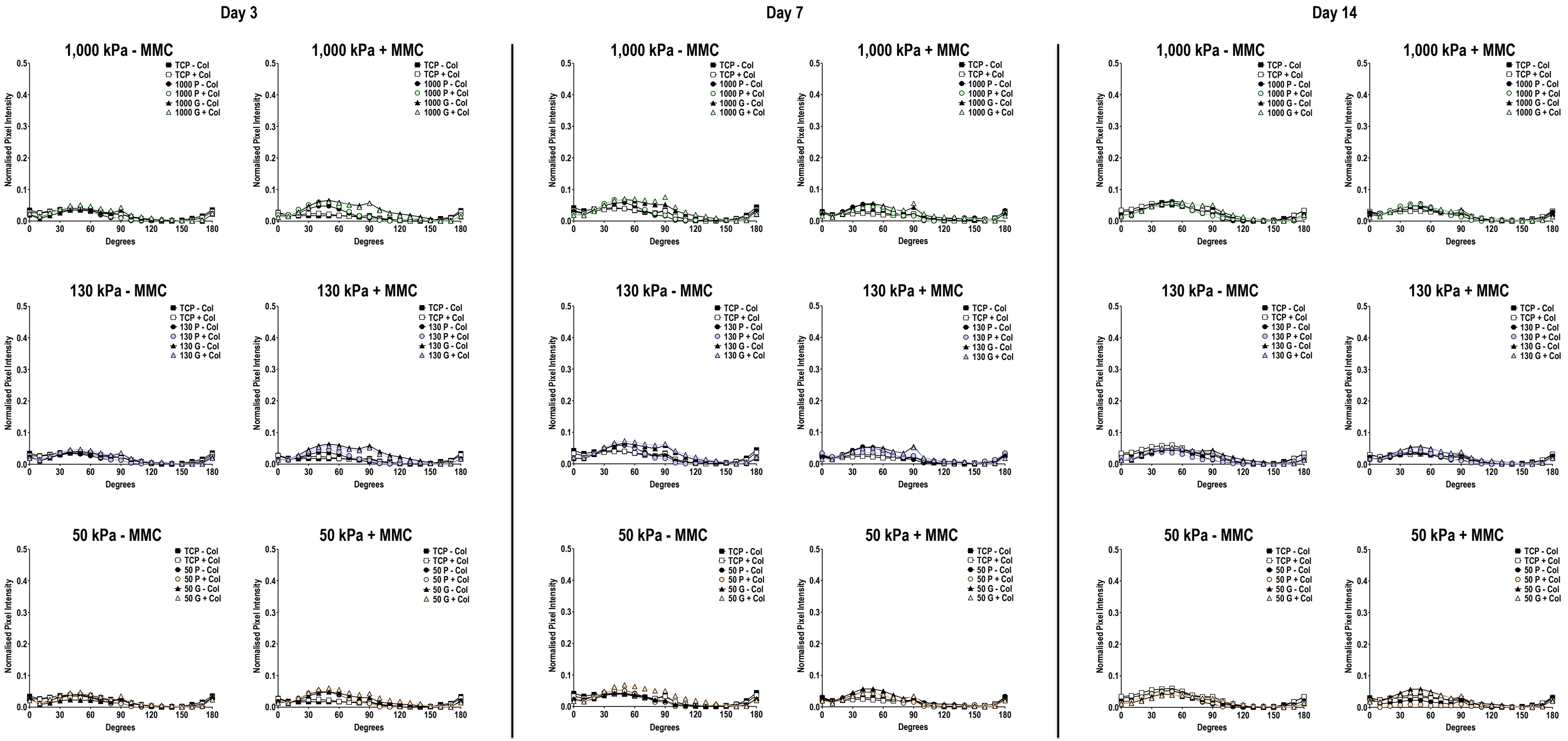

Supplementary Figure S29: Human tenocyte collagen type VI matrix orientation at day 3, day 7 and day 14 on tissue culture plastic (TCP) without and with collagen type I coating (- Col, + Col) and macromolecular crowding (- MMC, + MMC) and on substrates of varying stiffness (1,000 kPa, 130 kPa, 50 kPa), surface topography [planar (P), grooved (G)], collagen type I coating (- Col, + Col) and macromolecular crowding (- MMC, + MMC).

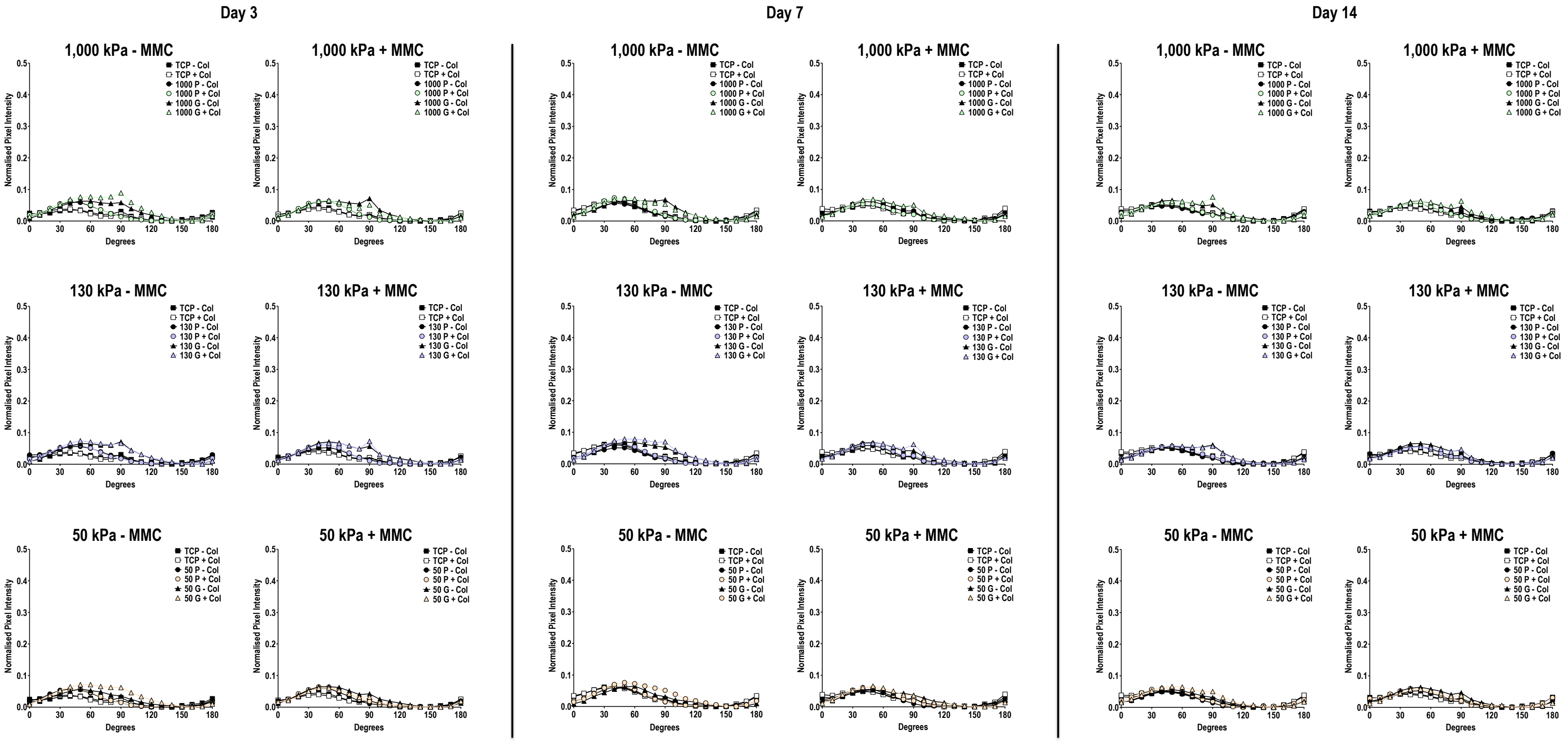

Supplementary Figure S30: Human tenocyte fibronectin matrix orientation at day 3, day 7 and day 14 on tissue culture plastic (TCP) without and with collagen type I coating (- Col, + Col) and macromolecular crowding (- MMC, + MMC) and on substrates of varying stiffness (1,000 kPa, 130 kPa, 50 kPa), surface topography [planar (P), grooved (G)], collagen type I coating (- Col, + Col) and macromolecular crowding (- MMC, + MMC).

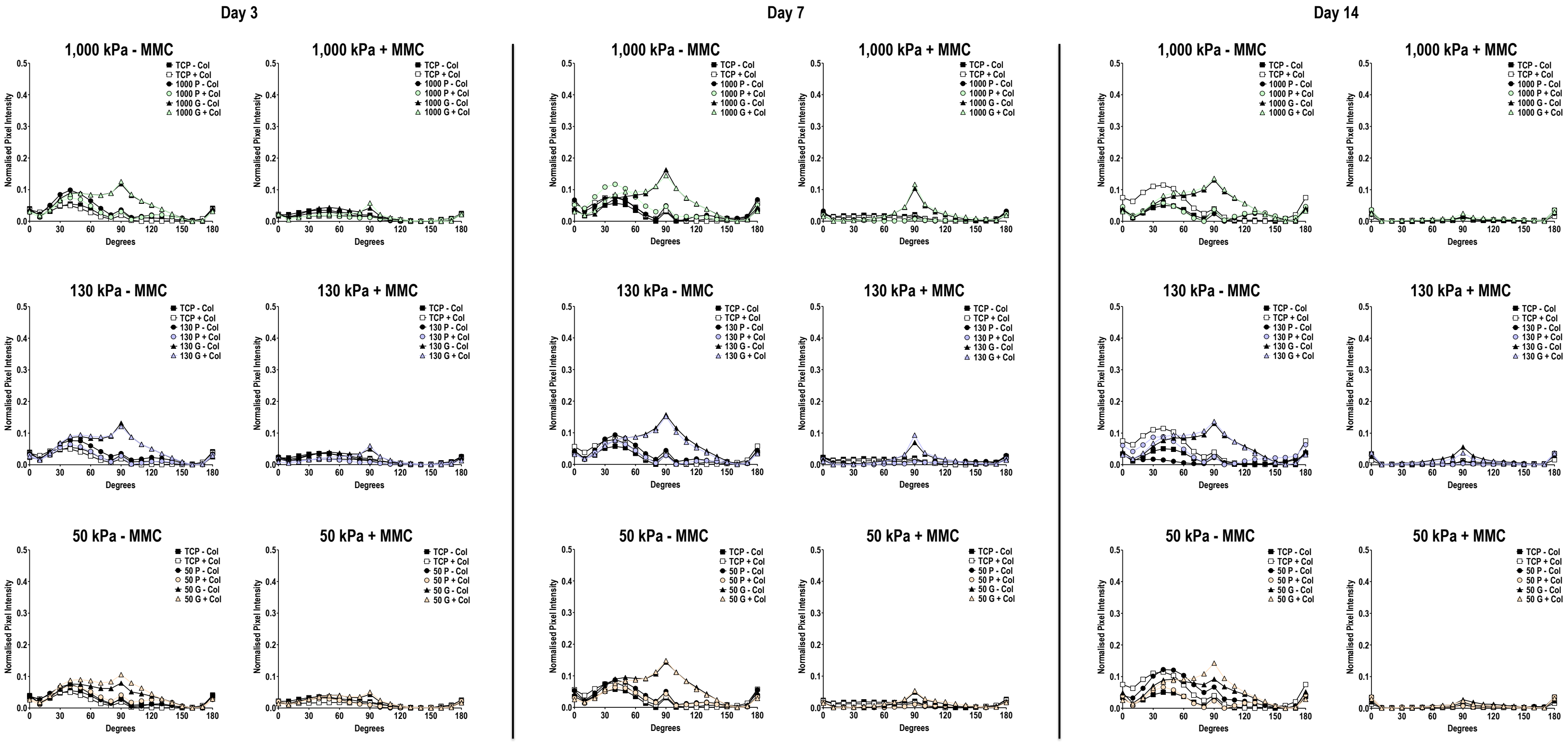

Supplement: Multimedia component 1 [file mmc1.pdf]
